# Whole-tree harvesting improves the ecosystem N, P and K cycling functions in secondary forests in the Qinling Mountains, China

**DOI:** 10.3389/fpls.2024.1394112

**Published:** 2024-12-20

**Authors:** Yue Pang, Jing Tian, Qiang Liu, Dexiang Wang

**Affiliations:** ^1^ College of Forestry, Hebei Agricultural University, Baoding, China; ^2^ College of Forestry, Northwest A&F University, Yangling, Shaanxi, China

**Keywords:** Nutrient cycling functions, whole-tree harvesting, secondary forests, ecosystem level, thinning intensities

## Abstract

Forest ecosystem nutrient cycling functions are the basis for the survival and development of organisms, and play an important role in maintaining the forest structural and functional stability. However, the response of forest nutrient cycling functions at the ecosystem level to whole-tree harvesting remains unclear. Herein, we calculated the ecosystem nitrogen (N), phosphorus (P), and potassium (K) absorption, utilization, retention, cycle, surplus, accumulation, productivity, turnover and return parameters and constructed N, P, and K cycling function indexes to identify the changes in ecosystem N, P, and K cycling functions in a secondary forest in the Qinling Mountains after 5 years of five different thinning intensities (0% (CK), 15%, 30%, 45%, and 60%). We showed that the ecosystem’s N, P, and K cycling parameters varied significantly and responded differently to thinning treatments. As the thinning intensity increased, the N, P, and K cycling function indexes increased by 5%~232%, 32%~195%, and 104%~233% compared with CK. Whole-tree harvesting promoted ecosystem N and P cycling functions through two pathways: (a) directly regulated litter biomass, indirectly affected soil nutrient characteristics, and then regulated ecosystem N and P cycling functions; (b) directly regulated plant productivity, indirectly affected plant and soil nutrient characteristics, and then regulated ecosystem N and P cycling functions. In contrast, whole-tree harvesting mainly indirectly affected the plant and soil nutrient characteristics by directly adjusting the plant productivity, and promoting the ecosystem K cycling function. Furthermore, N and P cycling functions were mainly regulated by understory plant productivity while tree and herb nutrient characteristics were key driving factors for K cycling functions. These findings indicated that whole-tree harvesting significantly improved the ecosystem N, P and K cycling functions, and reveals varied regulatory mechanisms, which may aid in formulating effective measures for sustainable forest ecosystem nutrient management.

## 1 Introduction

Nutrient utilization is critical for the survival and growth of organisms in forest ecosystems. The cycling and balance of essential nutrients such as nitrogen (N), phosphorus (P), and potassium (K) are key to maintaining the stability and long-term productivity of these ecosystems (Tamm, 2012; Qi et al., 2022). Nutrient cycling in forest ecosystems involves nutrient exchange, absorption, distribution, and return between ecosystem components and the environment (Gérard et al., 2008; Johnson and Turner, 2019; Legout et al., 2020). Previous research has largely focused on nutrient cycling characteristics in individual ecosystem components (Zhang et al., 2018a; Hou et al., 2021; Yan et al., 2019) and the development of parameters such as nutrient use efficiency and nutrient enrichment coefficients at both regional and global scales (Li, 1997; Turner and Lambert, 2015), which greatly advanced research on nutrient cycling mechanisms and deepened our theoretical understanding of nutrient cycling in forest ecosystems. However, few reports explore nutrient cycling functions from an ecosystem perspective. In nature, forest ecosystems are composed of different ecological components, and nutrient recycling processes are interconnected. Therefore, it is necessary to research forest ecosystem nutrient cycling functions, which is essential for understanding forest ecosystem structure and function and elucidating nutrient cycling mechanisms.

Ecosystem functions are the basis of stable and sustainable ecosystem development (Hector and Bagchi, 2007). The ecosystem function index is often used to quantify the ability of an ecosystem to provide and maintain ecosystem functions. Research based on this theoretical parameter has been reflected in many fields. For instance, Jia et al. (2022) explored associations between soil quality and ecosystem multifunctionality driven by fertilization management in the North China Plain. Yang et al. (2023) studied responses of soil microbial diversity, network complexity and multifunctionality to three land-use changes at spatial-temporal scales. Moi et al. (2022) focused on the analysis of biodiversity-multifunctionality relationships under human pressure in large Neotropical wetlands. As mentioned above, the ecosystem function index has been widely developed in the study of ecosystem multifunctional mixtures, providing substantial data for ecosystem function evaluation. However, the concept of this function index is rarely used to assess specific ecosystem nutrient cycling functions, and research in this area is crucial for forest health and sustainable development. Thus, constructing the forest ecosystem nutrient cycling function indices is essential for expanding our knowledge about forest ecosystem functions.

Forest ecosystem nutrient cycling functions are complex and are influenced by both biotic and abiotic factors; simultaneously, nutrient elements have heterogeneous cycling patterns due to their different roles in the ecosystem (Johnson and Lindberg, 2013; Vitousek, 2018). Forest thinning, a key forest management practice, modifies the community structure, directly affects the distribution of light, heat, water and other resources in the forest ecosystem, and indirectly influences resource competition, litter quality, and soil nutrient fertility within vegetation communities, potentially impacting nutrient cycling and balance (Zhang et al., 2018b; Li et al., 2020). For instance, some studies have shown that thinning removes a large number of branches and leaves, resulting in nutrient loss and decreased soil fertility (Chen et al., 2016; Garrett et al., 2021; Chen C. et al., 2023). In contrast, some other researchers have found that thinning may also reduce community nutrient competition pressure and accelerate nutrient recycling rates, thereby increasing the nutrient concentration of fresh leaves and litter and reducing the nutrient reabsorption rates (Ma et al., 2018; Qiu et al., 2020). However, relatively few studies have examined how forest nutrient cycling functions respond to whole-tree harvesting, even though whole-tree harvesting removes more aboveground tree parts (stems, needles, branches, twigs) than conventional stem-only harvesting, resulting in more significant changes in community structure and ecosystem nutrient loss (Yan et al., 2017; Pang et al., 2021b). Moreover, the regulatory mechanisms of forest thinning on nutrient cycling functions are not yet fully understood and require further investigation. Therefore, investigating the response rules of forest ecosystem nutrient cycling functions following whole-tree thinning and clarifying the regulatory pathways are crucial for revealing the nutrient cycling mechanism following thinning and can provide further insights into formulating a reasonable forest management system.

The Qinling Mountains are significant secondary forest regions located in the transitional zone between the subtropical and warm temperate zones of central China (Pang et al., 2022). So far, Qinling secondary forests cover 80% of the Qinling forest area under the protection of the “Natural Forest Protection Program”, making great contributions to the region’s vegetation reconstruction, soil and water conservation, carbon fixation and oxygen release (Yu et al., 2023). Nevertheless, these secondary forests have poor growth conditions, low ecological functions, and vulnerable stability. Whole-tree harvesting, a common forest management practice in the Qinling Mountains, is used to improve forest productivity and restore ecological functions. However, this practice can result in significant nutrient loss, raising concerns about its long-term effects on forest health and nutrient cycling functions. Numerous studies have evaluated the variation in the vegetation community structures, soil physical and chemical properties and microbial metabolic activity (Ren et al., 2018; Kang et al., 2022); but, the effects of whole-tree harvesting on nutrient cycling functions in these secondary forests at the ecosystem level are not well understood.

This study aims to: (1) analyze the nutrient cycling characteristics of the secondary forest ecosystem following whole-tree harvesting, and (2) construct nutrient cycling function indexes to explore the mechanisms driving changes in nutrient cycling. Thus, we measured the C, N, P, and K nutrient characteristics of trees, shrubs, herbs, litter, and soil at the plot level in secondary forests in the Qinling Mountains after 5 years of five thinning intensities (CK: 0%, T1: 15%, T2: 30%, T3: 45%, T4: 60% of the stand volume removed), and calculated the ecosystem N, P, and K absorption, utilization, retention, cycle, surplus, accumulation, productivity, turnover and return parameters. We further constructed the ecosystem N, P, and K cycling function indexes and analyzed the relationships among ecosystem nutrient cycling functions and forest stand characteristics, soil properties and ecosystem component nutrient characteristics. We hypothesized that (1) there may be significant differences in the ecosystem N, P, and K cycling characteristics due to the heterogeneous effects of nutrient elements; (2) whole-tree harvesting would have a negative impact on the ecosystem N, P, and K cycling functions because whole-tree harvesting causes substantial nutrient loss; (3) whole-tree harvesting may regulate nutrient cycling functions differently due to the significant disturbances it causes to vegetation, soil, and the overall environment.

## 2 Materials and methods

### 2.1 Site description and experimental design

The study was performed in the Qinling National Forest Ecosystem Research Station (33°18′-33°28′N, 108°21′-108°39′E), which is located in Ningshaan County, Shaanxi Province, China. The area covers 22.25 km^2^ and has a subtropical humid montane climate. The average annual temperature is 10.5°C, and the annual precipitation is 1000 mm, with an average frost-free period of 199 days and a growth period of 177 days (Pang et al., 2021b, 2022). The soil types in the sampling area are classified as Cambisols, Umbrisols, and Podzols according to the Food and Agriculture Organization (FAO) classification system. Across the site, the average soil layer thickness and humus matter thickness are 50 cm and 8 cm, respectively. The forests underwent rotational felling or firewood cutting during the 1960s and 1970s at this research station. After natural regeneration, secondary growth dominated the study area, and the dominant tree species are *Quercus aliena* var. *acutiserrata*, *Quercus variabilis*, *Pinus armandii*, *Betula albosinensis*, *Picea asperata*, and *Populus davidiana*. Additionally, shrubs including *Lonicera tragophylla*, *Cerasus stipulacea*, and *Symplocos paniculate*, as well as herbs including *Lysimachia christinae*, *Rubus parvifolius*, *Saussurea mutabilis*, and *Rubia cordifolia* occupy the understory space.

To guide the development of secondary forests towards a healthy stand structure, this study implemented a target tree operation system to transform the existing secondary forests based on the principles of near-natural forest management. Based on field surveys, we selected secondary stands with similar geographical and micro-topographic conditions. The whole-tree harvesting experiment with a complete randomized block design with 5 treatments and 4 replicates, making a total of 20 plots (20 m in length × 20 m in width), were established in the secondary forests in September 2013. At the same time, tree height and diameter at breast height (DBH ≥ 5 cm, measured at 1.3 m) were recorded for each plot. The treatments were 5 levels of thinning intensity: no thinning as a control with (CK), 15% removal of the stand volume (T1), 30% removal of the stand volume (T2), 45% removal of the stand volume (T3), 60% removal of the stand volume (T4). To avoid potential edge effects, a 5-m-wide buffer zone was established around each plot. All harvested materials, including leaves, branches, stems, bark, and twigs, were removed from the plots. We conducted the survey and sampling for this study in 2018, 5 years after the whole-tree harvesting treatment. Detailed setup information about the blocks and plots are presented in **Supplementary Figure S1**.

### 2.2 Plant, litter and soil sample collection and survey

Plant samples were collected in August 2018 during the peak growing season. For trees, the tree height and diameter at breast height (DBH ≥ 5 cm, 1.3 m) in each plot were measured again before sampling, and the trees were also classified and counted by species. Tree leaves, branches, stems, bark, and roots were then sampled by species through pruning, cutting, drilling, and digging, as detailed in our previous study (Pang et al., 2021a). We calculated the tree organ biomass based on the allometric equations developed for trees in or near the study area (**Supplementary Table S1**). According to the biomass ratios of different species organs, the different organ samples of the trees were mixed evenly. To evaluate the species diversity of shrub and herb, five shrub subplots (2 × 2 m) and five herb subplots (1 × 1 m) were established in each plot, and a whole-plant sampling technique was used to collect shrub and herb biomass. After dividing the shrubs into leaves, branches, and roots, and the herbs into aboveground and belowground parts, then mixed into a uniform sample.

At the plot level, we used a root auger (90 mm diameter) to collect 9 fine root point samples at depths of 0–20 cm, 20–40 cm and 40–60 cm in September and November 2018, and April and June 2019. According to the method described by Brassard et al. (2013), the fine roots were divided into living and dead groups, including three diameter classes (<0.5 mm, 0.5–1mm, and 1–2 mm). For litter sampling, six litter traps (90 cm in diameter) were fixed 1 m above the ground to collect tree litter three times in one year, and all the ground litter was collected in five 1 × 1 m subplots. Litter was categorized into leaves, branches, and miscellaneous material. Eventually, all subsamples of plant and litter were transported to the laboratory and oven-dried at 70 °C to constant weight.

The soil samples were collected from 9 replicate points along an “S” shape in each plot at 0–20 cm, 20–40 cm and 40–60 cm depths by using a 40 mm diameter stainless-steel auger and mixing the samples into one composite sample per plot. The soil samples were then immediately sieved through a mesh < 2 mm, and stones, plant roots, fauna, and debris were removed. Each soil sample was divided into three parts, the first part was used to determine the soil moisture content; the second part was airdried for physicochemical analyses; and the other portion was stored at 4°C for microbial biomass analyses. Soil bulk density samples were obtained randomly from three points per plot by volumetric rings (100 cm^3^).

### 2.3 Laboratory analyses

All the chemical indicators of plant, litter and soil samples were determined following a previously described method (Bao, 2000). The carbon (C) contents in plant, litter and soil (SOC) samples were analyzed using the K_2_Cr_2_O_7_ oxidation method, while total nitrogen (TN) and total phosphorus (TP) contents were analyzed by colorimetry with an automatic discontinuous elemental analyzer (Clever chem200+, Germany) after wet digestion. Plant, litter and soil total potassium (TK) and soil available potassium (AK) were measured through a flame photometry. Soil available nitrogen (AN) and available phosphorus (AP) were determined by alkaline hydrolysis diffusion and colorimetry respectively. Soil pH was measured in a 1:2.5 soil:water suspension using a glass-electrode meter. Soil moisture content was determined by oven-drying fresh soil at 105°C for 48 hours to a constant weight. Similarly, the volumetric ring soil was dried at 105 °C to constant weight, and the ratio of soil mass to total volume (g·cm^−3^) was calculated to obtain the soil bulk density (De Vos et al., 2005). Microbial biomass C (MBC), N (MBN) and P (MBP) were quantified with a total organic C analyzer (TOC-LCSN, Shimadzu Co., Japan), flow analyzer (AutoAnalyzer 3, Seal Analytical Ltd., UK) and spectrophotometer (UV-1900, Shimadzu Co., Japan) after fumigation (Kim et al., 2018).

### 2.4 Data calculation and analysis

#### 2.4.1 Biomass, productivity, nutrient stock, nutrient accumulation rate and nutrient return calculation

Referring to the National Standard of the People’s Republic of China “Long-term Positioning Observation Method of Forest Ecosystems” and “Technical Regulations for Continuous Inventory of Forest Resources”, the biomass and productivity of trees, shrubs and herbs, the existing biomass and annual biomass of litter, and the total biomass and productivity of the forest ecosystem were calculated respectively. Furthermore, combined with the C, N, P and K characteristics of plant, litter, and soil, the C, N, P and K stocks and accumulation rates across tree, shrub, and herb layers, the C, N, P and K return amounts of aboveground and belowground parts, the C, N, P and K stocks in the soil layer, and the C, N, P and K stocks, plant C, N, P and K accumulation rates, and plant C, N, P and K return amounts at the ecosystem level were obtained. Detailed methods used to quantify the above parameters are included in the SI Materials and Methods.

#### 2.4.2 Ecosystem nutrient cycling parameter calculation

The calculation of ecosystem nutrient cycling parameters refers to the method described by Liu (2009).

Nutrient flux parameters:


(1) TR=PR+RI−RO

Where *TR* is total N, P or K return amounts (kg·ha^-1^·year^-1^), *PR* is plant N, P or K return amounts (kg·ha^-1^·year^-1^, ecosystem plant N, P or K return amounts); *RI* is rainfall N, P or K input amounts (kg·ha^-1^·year^-1^): the rainfall nutrient input amounts in this research were determined by reviewing the literature on rainfall nutrient content that has been reported in this study area (Zhang, 2005; Zhao, 2015; Li, 2017), N: 5.44 kg·ha^-1^·year^-1^, P: 0.268 kg·ha^-1^·year^-1^, K: 11 kg·ha^-1^·year^-1^ respectively; *RO* is N, P or K runoff output amounts (kg·ha^-1^·year^-1^): Note that the runoff output in this study area is dominated by subsurface runoff, and the output and input up and down slopes are basically in balance (Zhang et al., 2000). Therefore, no runoff nutrient export is assumed in this study area.


(2) NB=CL+TR

Where *NB* is N, P or K absorption amounts (kg·ha^-1^·year^-1^, absorbed by the ecosystem plant layer in one year), *CL* is N, P or K retention amounts (kg·ha^-1^·year^-1^, ecosystem plant N, P or K accumulation rate).

Nutrient supply capacity parameters:


(3) SP=TR−NB

(4) $$SPR=\frac{TR-NB}{SW}\times 100$$

where *SP* is soil N, P or K surplus amount (kg·ha^-1^·year^-1^), *SPR* is soil N, P or K surplus percentage (%), *SW* is soil N, P or K stock (kg·ha^-1^·year^-1^); *SP* and *SPR* describe the ecosystem N, P or K supply capacity.


(5) $$NP=\frac{PP}{NB}$$

Where *NP* is N, P or K productivity (t·kg^-1^), *PP* is plant productivity(t·ha^-1^·year^-1^); *NP* represents the dry matter mass that can be produced per unit weight of N, P or K absorption.

Nutrient cycling parameters:


(6) $$ax=\frac{NB}{SW}$$

Where *ax* is N, P or K absorption coefficient; *ax* represents the N, P or K absorption capacity of plant roots from the soil N, P or K pool.


(7) $$ux=\frac{NB}{FS}$$

Where *ux* is N, P or K utilization coefficient, *FS* is total N, P or K stocks in the forest stand; *ux* represents the plant’s ability to maintain the N, P or K required for growth at the ecosystem level.


(8) $$cx=\frac{TR}{NB}$$

where *cx* is N, P or K cycle coefficient; the *cx* represents the cycle intensity of N, P or K in the ecosystem, and the larger the value, the faster the cycle rate.


(9) $$rx=\frac{1}{1-cx}$$

where *rx* is N, P or K recycling coefficient; the *rx* represents the average recycling numbers of a unit substance before leaving the system, which can better reflect the forest land soil N, P or K status under severe human interference.


(10) $$ncx=\frac{CL}{NB}$$

Where *ncx* is N, P or K retention coefficient; the *ncx* represents the plant’s ability to retain N, P or K, and the smaller the value, the higher the nutrient utilization efficiency.


(11) $$pt=\frac{PS}{TR}$$

Where *pt* is plant N, P or K turnover time coefficient, *PS* is plant N, P or K stock; *pt* represents the time required for the plant N, P or K pool to go through a cycle, and the larger the value, the more N, P or K the plant needs.


(12) $$pax=\frac{NB}{PS}$$

Where *pax* is plant N, P or K stock accumulation coefficient; the *pax* reflects the accumulation rate of plant N, P or K stock pools.

#### 2.4.3 Ecosystem nutrient cycling function index

The ecosystem function index was employed to systematically extract comprehensive information from nutrient flux, nutrient supply capacity, and nutrient cycling parameters.

First, the Z-scores of the ecosystem nutrient cycling parameters were calculated in each plot.


(13) $$Z_{ij}=\frac{x_{ij}-\mu_{j}}{\sigma_{j}}$$

Where *Z_ij_* represents the Z-score of the *j-th* N, P or K cycling parameter in plot *i*; *x_ij_* represents the value of the *j-th* N, P or K cycling parameter in plot *i*; *μ_j_* represents the mean value of the *j-th* N, P or K cycling parameter; *σ_j_* represents the *j-th* N, P or K cycling parameter standard deviation; where *i* ranges from 1-20 and *j* ranges from 1-12.

Then, the Z-scores of the nutrient cycling parameters in each plot were averaged, which is the ecosystem nutrient cycling function index (M) in each plot.


(14) $$M_{i}=\frac{\sum_{j=1}^{n}Z_{ij}}{n}$$

Where *M_i_* is the secondary forest ecosystem N, P, or K cycling function index of the *i-th* plot, n is the number of nutrient cycling parameters involved in the calculation, n=12.

#### 2.4.4 Basis for selection of factors affecting ecosystem nutrient cycling function

The forest ecosystem nutrient cycling functions are affected by complex and diverse biotic and abiotic factors. Referring to previous studies, 58 explanatory factors were initially screened. Among them, the forest stand characteristics of tree, shrub, herb, litter and fine root (<0.5mm, 0.5-1mm, 1-2mm, <2mm) productivity, shrub and herb diversity, and litter biomass can reflect the nutrient competition diversity and consumption level in the community. Soil pH, moisture content, and bulk density are key parameters that affect vegetation growth and development and soil nutrient mineralization, and have an important impact on ecosystem nutrient cycling. The nutrient pool characteristics of the soil (C, N, P, K, C:N, C:P, N:P, AN, AP, AK, MBC, MBN, MBP, MCN (MBC: MBN), MCP (MBC: MBP), MNP (MBN: MBP)), plant (C, N, P, K and their stoichiometric ratios of tree, shrub, and herb functional groups at the ecosystem level, see details in SI Materials and Methods) and litter (GC (litter C), GN (litter N), GP (litter P), GK (litter K), GC:N (GC: GN), GC:P (GC: GP), GN:P (GN: GP)) are all the ecosystem nutrient cycling components and promote the development of the ecosystem nutrient cycling function.

#### 2.4.5 Statistical analysis

All data were checked for normality and homogeneity of variance and transformed if necessary. A linear mixed-effect model was used to evaluate the statistical significance of ecosystem components (plant, litter and soil) and ecosystem biomass, productivity, C, N, P and K stocks, C, N, P and K accumulation rates, C, N, P and K return amounts, C, N, P and K cycling parameters, and N, P and K cycling functions under different thinning intensities. The models included thinning intensities, plant organ components, soil layers, nutrient element categories or their interaction terms as the fixed factors and block or block and plot as the random factors. For all models, the significance of fixed effects was assessed using Satterthwaite approximations for degrees of freedom. When fixed effects or interactions were significant, the least square means differences test was used for multiple comparisons. Pearson correlation and Boruta feature selection were performed to explore the correlation between ecosystem N, P, and K cycling function indexes and influencing factors, and to determine optimal predictors. Variation partitioning analysis (VPA) was employed to analyze the contribution of category driver factors to changes in ecosystem N, P and K cycling functions. Partial least squares path modeling (PLS-PM) was used to further identify the possible pathways that whole-tree harvesting regulates the ecosystem N, P and K cycling functions. All analyses were implemented using R for Windows version 4.1.1 statistical software (R Development Core Team, 2017).

## 3 Results

### 3.1 Biomass, productivity, nutrient stock, nutrient accumulation rate and nutrient return

The biomass and productivity of tree and ecosystem, and ground litter biomass were significantly reduced with increasing thinning intensity, whereas shrub and herb biomass and productivity showed the opposite trend (**Supplementary Figures S2–S9**, p < 0.05). Although litter miscellaneous productivity was significantly higher in T2 and T3 than in other thinning treatments (**Supplementary Figure S8A**, p < 0.05), total litter productivity did not respond significantly to the thinning treatments (**Supplementary Figure S8B**, p > 0.05).

Overall, thinning significantly reduced tree C, N, P and K stocks and accumulation rates, ground litter C, N, P and K stocks, soil C, N and P stocks, ecosystem C, N and P stocks and accumulation rates and ecosystem K accumulation rate, and significantly increased the ecosystem K stock and the stocks and accumulation rates of shrub and herb C and N, shrub P and herb K (**Supplementary Table S3** and **Supplementary Figures S10–S16**, p < 0.05). The shrub K and herb P stocks and accumulation rates were nonsignificant among the different thinning intensities (**Supplementary Table S3** and **Supplementary Figures S11G, H**, **S15G, H**, p > 0.05).

As shown in **Supplementary Figure S17**, the tree litter C concentration was highest in winter, while N, P, and K concentrations peaked during the spring-summer period while the return amounts of tree litter C, N, P, and K were significantly higher in autumn than in other seasons (p < 0.05). However, total aboveground litter C, N, P, and K return amounts did not respond significantly to thinning treatments (**Supplementary Figure S18**, p > 0.05).

Although the <0.5mm fine root in T2 and T4 exhibited significantly higher productivity, turnover rate, and C, N, P, and K return amounts than in other treatments (**Supplementary Figures S19**, **S20A–D**, p < 0.05), no significant differences were observed in total (<2mm) fine root C, N, P, and K return amounts across thinning intensities (**Supplementary Figures S20E-H**, p > 0.05).

Likewise, thinning did not significantly impact total plant C, N, P, and K return amounts at the ecosystem level (**Supplementary Figure S21**, p > 0.05).

### 3.2 Ecosystem nutrient cycling parameters and nutrient cycling function indexes among thinning treatments

**Figure 1:**
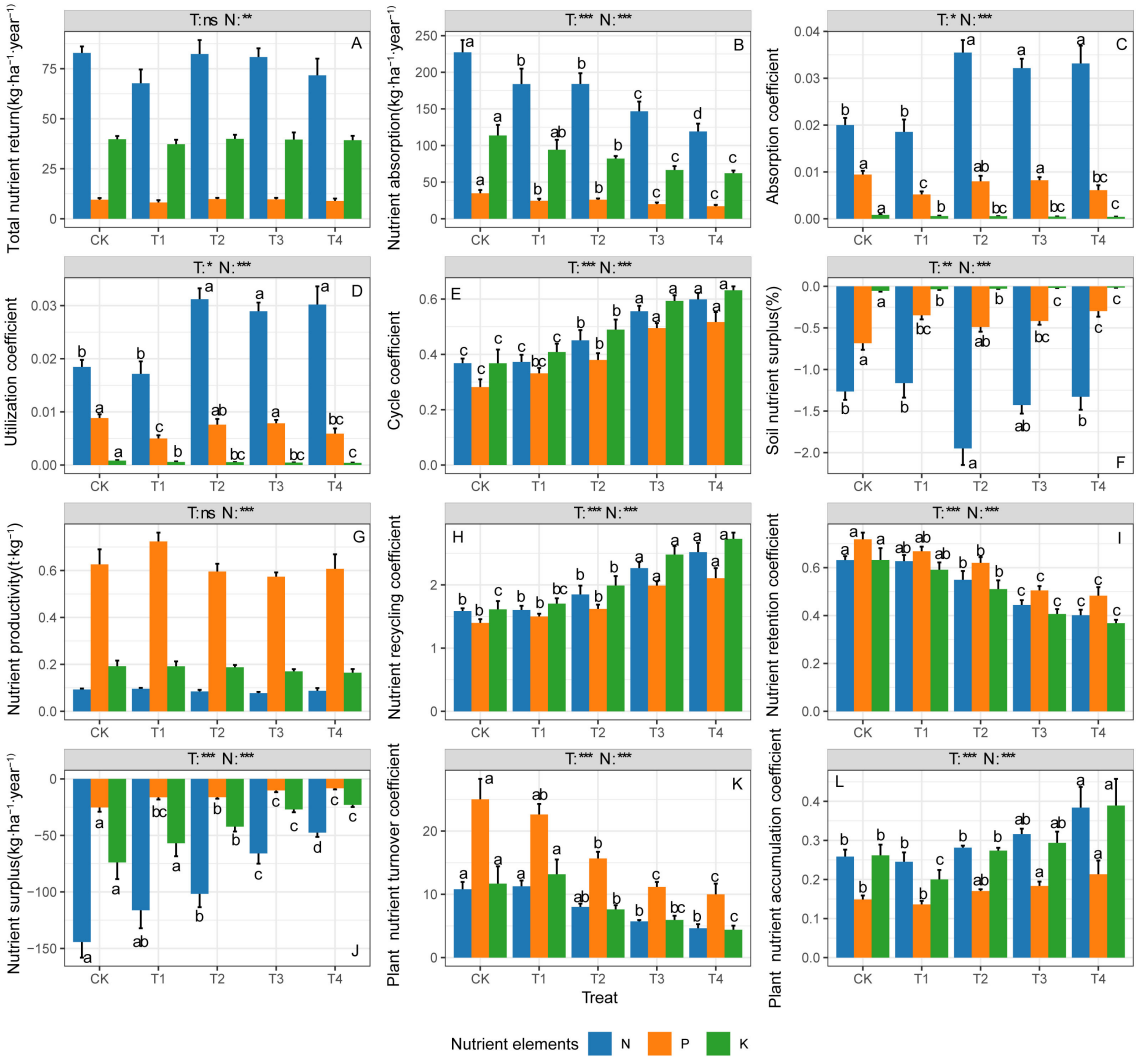
N, P and K cycling parameters in ecosystem under different thinning intensities. **(A)** Total nutrient return, **(B)** nutrient absorption, **(C)** absorption coefficient, **(D)** utilization coefficient, **(E)** cycle coefficient, **(F)** soil nutrient surplus (%), **(G)** nutrient productivity, **(H)** nutrient recycling coefficient, **(I)** nutrient retention coefficient, **(J)** nutrient surplus, (K) plant nutrient turnover coefficient, **(L)** plant nutrient accumulation coefficient. CK, T1, T2, T3, T4 represent 0%, 15%, 30%, 45% and 60% thinning intensity, respectively. T, treat; N, nutrient element. Different letters indicate significant differences between different thinning intensities. ns, non-significant, * p < 0.05, ** p < 0.01, *** p < 0.001.

Significant differences were observed among ecosystem N, P, and K cycling parameters, which showed varied responses to thinning treatments (**Figure 1**). The ecosystem N total return amounts, absorption amounts, absorption coefficient, and utilization coefficient were the highest (**Figures 1A–D**, p < 0.05), while the N surplus amounts and surplus rate were the lowest (**Figures 1F, J**, p < 0.05). The ecosystem P productivity, retention coefficient, and plant nutrient turnover time were at their highest levels (**Figures 1G, I, K**, p < 0.05). The ecosystem N and K cycle coefficient, recycling coefficient and plant nutrient accumulation coefficient were comparable and significantly higher than those parameters of P (**Figures 1E, H, L**, p < 0.05).

The ecosystem N, P, and K total return amounts and productivity had no significant responses to the thinning treatments (**Figures 1A, G**, p > 0.05). The N, P, and K absorption amounts, retention coefficients, and plant turnover nutrient time significantly decreased as thinning intensities increased (**Figures 1B, I, K**, p < 0.05), while the N, P and K cycle coefficient, recycling coefficient, surplus amounts, and plant nutrient accumulation coefficient significantly increased (**Figures 1E, H, J, L**, p < 0.05). Thinning significantly increased the ecosystem N absorption coefficient and utilization coefficient, and showed opposite effects on the P and K absorption coefficients and utilization coefficients (**Figures 1C, D**, p < 0.05). The N surplus rate in T2 was significantly lower than other treatments, while there was a significant increasing trend in ecosystem P and K surplus rates following thinning intensities (**Figure 1F**, p < 0.05).

**Figure 2:**
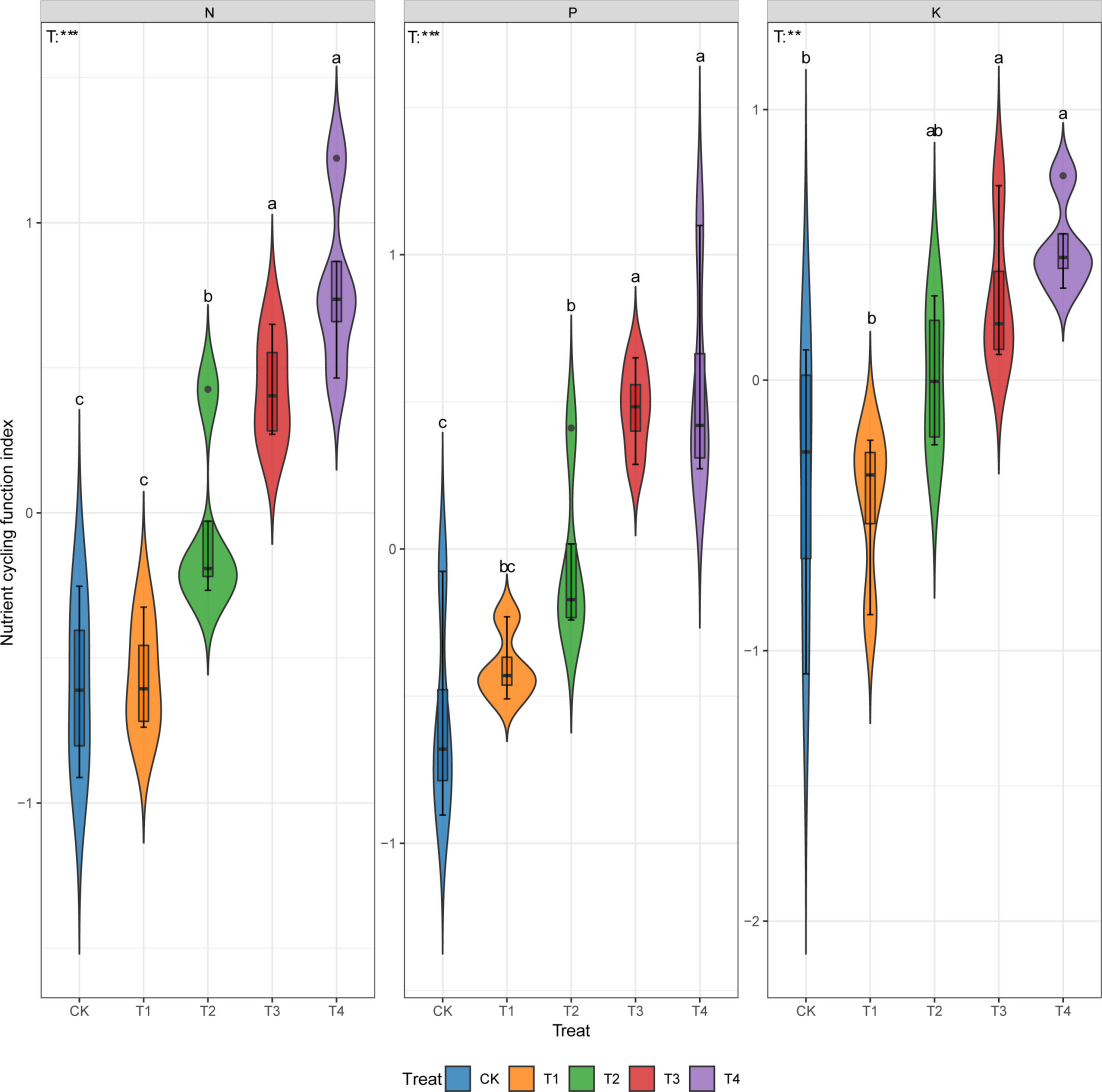
Ecosystem N, P and K cycling function indexes under different thinning intensities. CK, T1, T2, T3, T4 represent 0%, 15%, 30%, 45% and 60% thinning intensity, respectively. T: treat. Different letters indicate significant differences between different thinning intensities. ns, non-significant, ** p < 0.01, *** p < 0.001.

Compared with CK, thinning improved the ecosystem N, P, and K cycling function indexes by 5%~232%, 32%~195%, and 104%~233% respectively (**Figure 2**, p < 0.05).

### 3.3 Relationships between ecosystem nutrient cycling functions and stand characteristics, soil properties and ecosystem component nutrient characteristics

**Figure 3:**
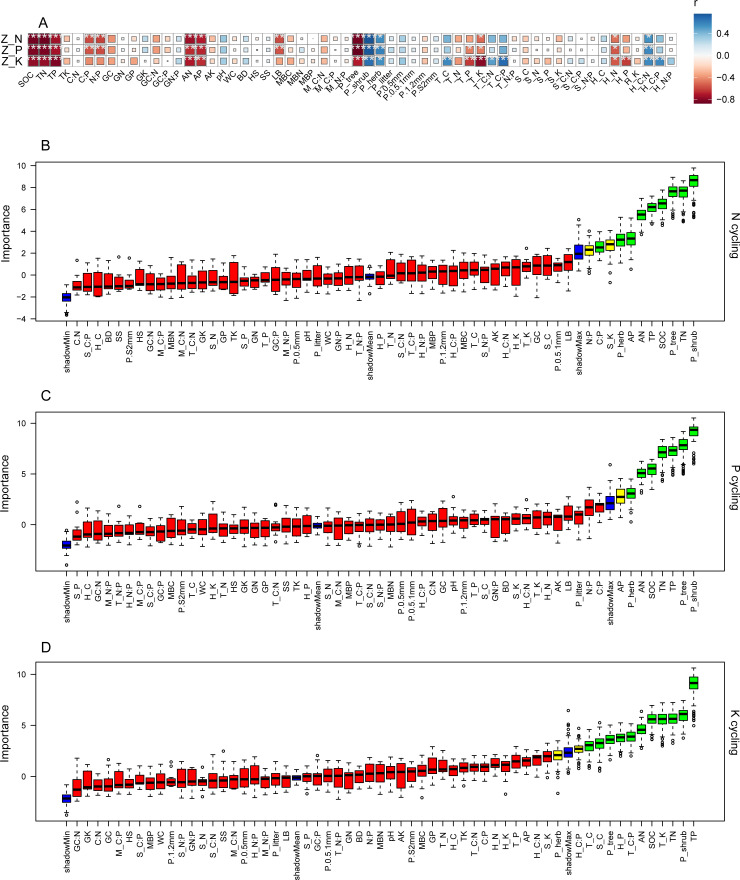
Correlation between ecosystem N, P and K cycling function indexes and influencing factors, and selection of important predictors for ecosystem N, P and K cycling functions. Z_N, Z_P, Z_K: N, P and K cycling function indexes; WC: water content; BD: soil bulk density; SS and HS: shrub and herb Shannon-Wiener indexes; LB: litter biomass; M_C:N, M_C:P, M_N:P: microbial biomass stoichiometric ratios; P_tree, P_shrub, P_herb, P_litter, P.0.5mm, P.0.5.1mm, P.1.2mm, P.S2mm: tree, shrub, herb, litter, <0.5mm, 0.5-1mm, 1-2mm and <2mm fine root productivity; T_C, T_N, T_P, T_K, S_C, S_N, S_P, S_K, H_C, H_N, H_P, H_K, T_C:N, T_C:P, T_N:P, S_C:N, S_C:P, S_N:P, H_C: N, H_C:P, H_N:P: C, N, P and K concentrations and stoichiometric ratios of trees, shrubs and herbs at the ecosystem level. **(A)**: red indicates a negative correlation and blue indicates a positive correlation; the darker the color and larger the size of the square is, the greater the absolute value of the correlation coefficient. **(B-D)** green and yellow columns represent selected important predictors, and red columns represent unselected factors. *p < 0.05, **p < 0.01, ***p < 0.001.

The ecosystem N, P, and K cycling function indexes were significantly negatively correlated with SOC, TN, TP, AN, AP, tree productivity, tree ecosystem level K, and herb ecosystem level N, while there were significant positive correlations between those nutrient cycling function indexes and the shrub and herb productivity, and the herb ecosystem level C:N (**Supplementary Tables S2–S4** and **Figure 3A**, p < 0.05). The ecosystem N and P cycling function indexes were significantly negatively related to soil C:P, N:P ratios and ground litter biomass (**Supplementary Tables S2–S4** and **Figure 3A**, p < 0.05). The tree ecosystem level P had negative correlations with the ecosystem P and K cycling function indexes (**Supplementary Tables S2–S4** and **Figure 3A**, p < 0.05). The ecosystem K cycling function index was positively correlated with the tree ecosystem level C, tree ecosystem level C:N, tree ecosystem level C:P and the herb ecosystem level C:P, but had the opposite effect on the herb ecosystem level P (**Supplementary Tables S2–S4** and **Figure 3A**, p < 0.05). The optimal predictors for ecosystem N, P, and K cycling function indexes, as identified by the Boruta method, aligned with with Pearson’s significantly correlated results (**Supplementary Tables S2–S4** and **Figures 3A–D**, p < 0.05).

**Figure 4:**
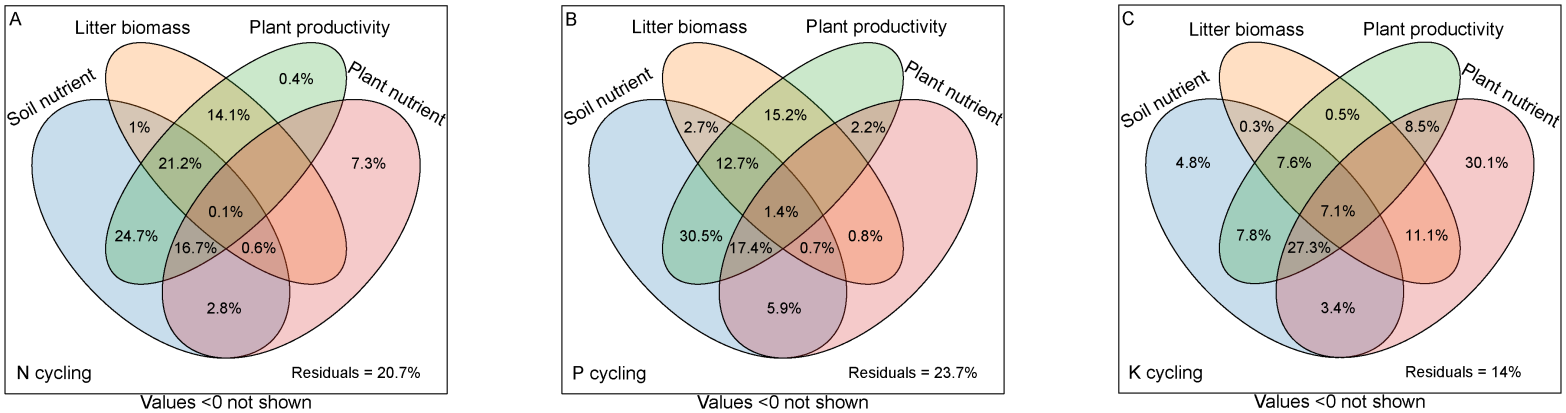
Variation partitioning analysis displaying the effects of soil nutrient, litter biomass, plant productivity and plant nutrient characteristics on ecosystem N, P and K cycling functions. **(A)** N cycling, **(B)** P cycling, **(C)** K cycling.

Based on the classification of selected predictors, the VPA analyses indicated that these soil nutrient, litter biomass, plant productivity and plant nutrient collectively explained the majority of variation associated with the ecosystem N, P, and K cycling functions (**Figure 4**). The soil nutrient, litter biomass, plant productivity and plant nutrient accounted for 67.1%, 37%, 77.2%, 27.5% of the variation of N cycling functions (**Figure 4A**), 71.3%, 33.5%, 79.4%, 28.4% of the variation of P cycling functions (**Figure 4B**), and 58.3%, 26.6%, 58.8%, 87.5% of the variation of K cycling functions (**Figure 4C**), respectively.

**Figure 5:**
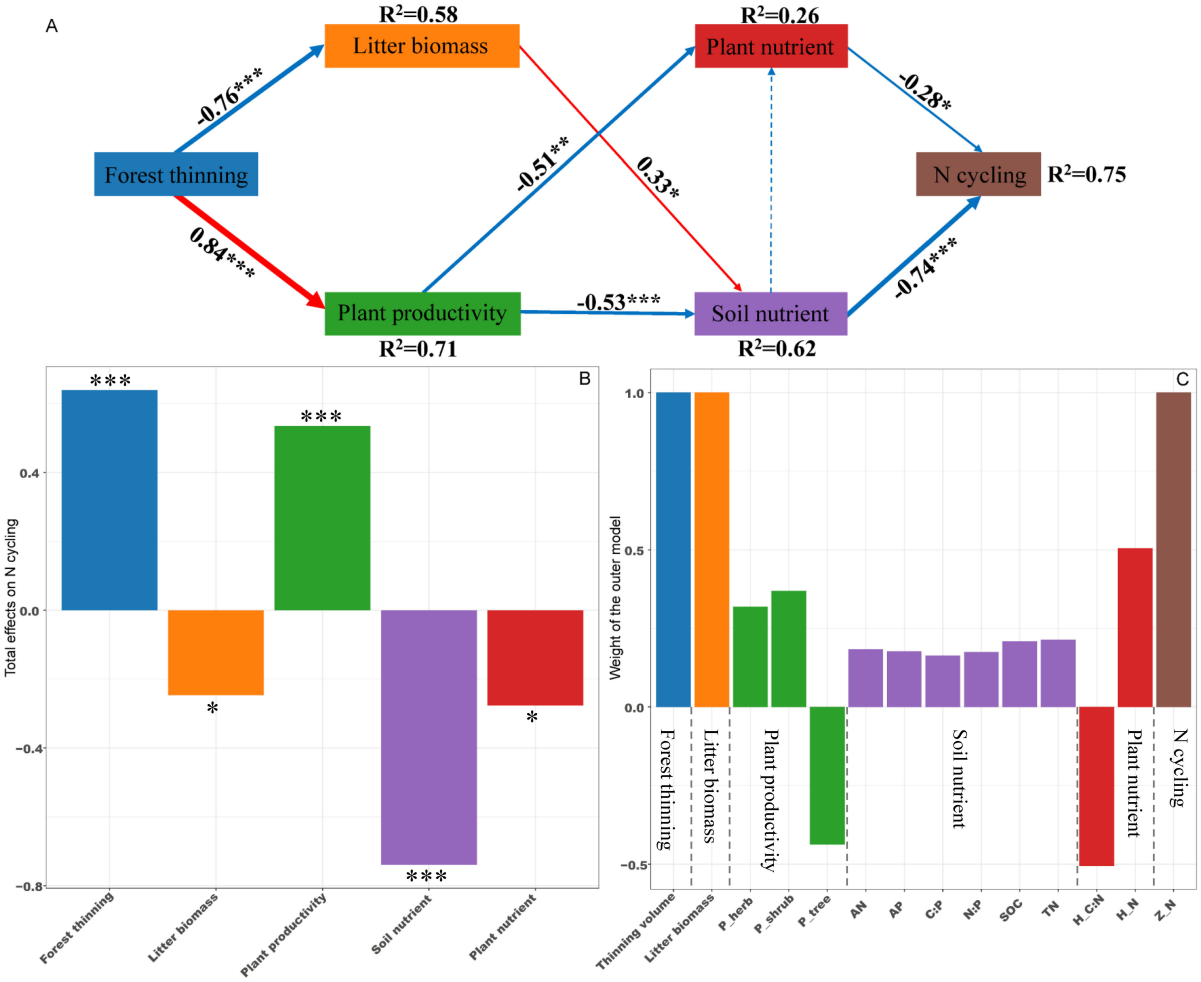
Partial least squares path analysis showing effects of thinning on ecosystem N cycling function. **(A)** Influence path of variables on N cycling, **(B)** total effects of variables on N cycling, **(C)** weight of the outer model. Red and blue arrows indicate positive and negative flows of causality. The width of the arrows indicates the strength of the path coefficient. Numbers on the arrow indicate significant standardized path coefficients. R^2^ indicates the variance of the dependent variable explained by the model. Z_N: N cycling function index; P_tree, P_shrub, and P_herb: tree, shrub and herb productivity; H_N and H_C:N: herb N concentration and C:N ratio at the ecosystem level. * p < 0.05, ** p < 0.01, *** p < 0.001.

**Figure 6:**
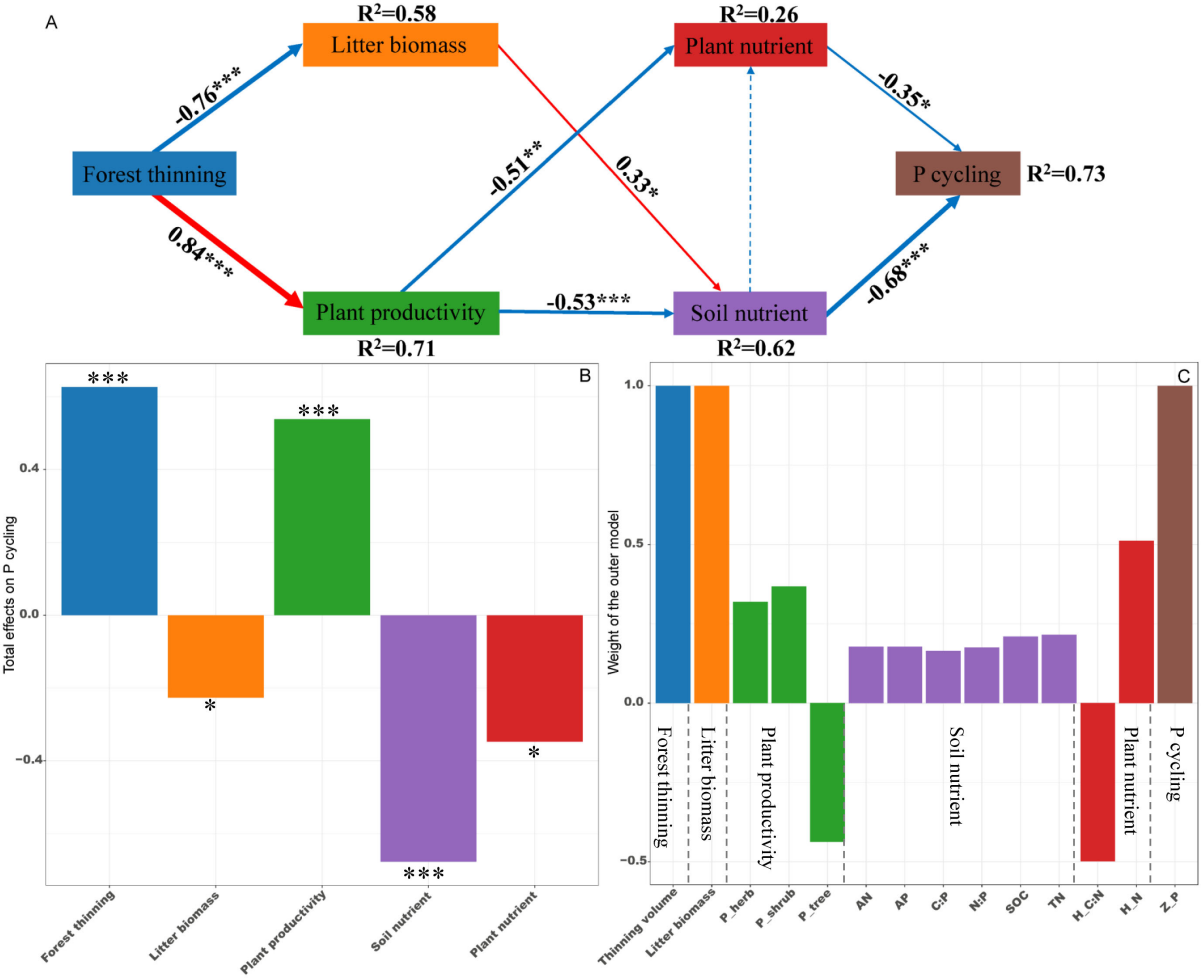
Partial least squares path analysis showing effects of thinning on ecosystem P cycling function. **(A)** Influence path of variables on P cycling, **(B)** total effects of variables on P cycling, **(C)** weight of the outer model. Red and blue arrows indicate positive and negative flows of causality. The width of the arrows indicates the strength of the path coefficient. Numbers on the arrow indicate significant standardized path coefficients. R^2^ indicates the variance of the dependent variable explained by the model. Z_P: P cycling function index; P_tree, P_shrub, and P_herb: tree, shrub and herb productivity; H_N and H_C:N: herb N concentration and C:N ratio at the ecosystem level. * p < 0.05, ** p < 0.01, *** p < 0.001.

**Figure 7:**
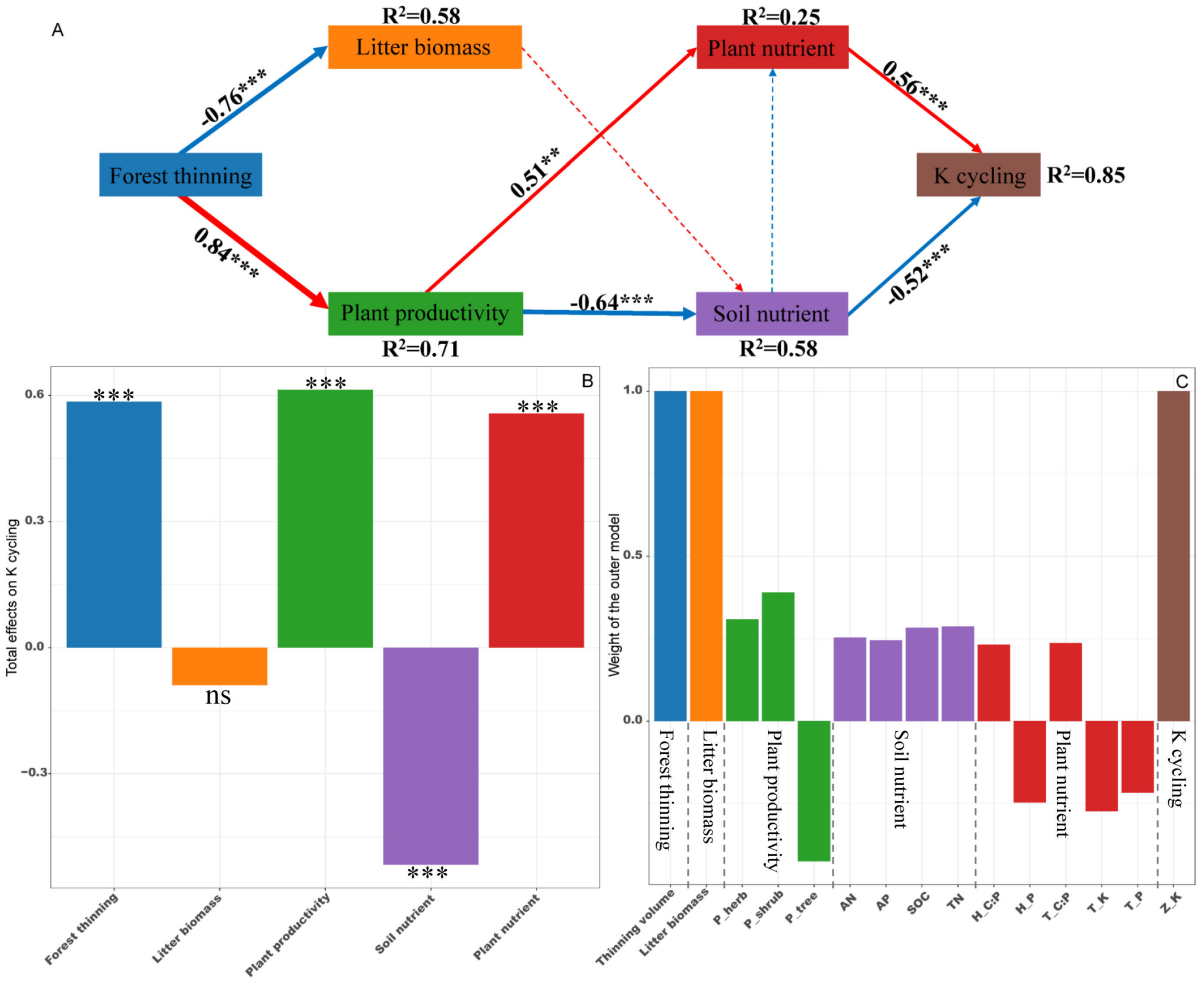
Partial least squares path analysis showing effects of thinning on ecosystem K cycling function. **(A)** Influence path of variables on K cycling, **(B)** total effects of variables on K cycling, **(C)** weight of the outer model. Red and blue arrows indicate positive and negative flows of causality. The width of the arrows indicates the strength of the path coefficient. Numbers on the arrow indicate significant standardized path coefficients. R2 indicates the variance of the dependent variable explained by the model. Z_K: K cycling function index; P_tree, P_shrub, and P_herb: tree, shrub and herb productivity; H_P, H_C:P, T_P, T_K and T_C:P: herb P concentration, herb C:P ratio, tree P concentration, tree K concentration and tree C:P ratio at the ecosystem level. ns: non-significant, ** p < 0.01, *** p < 0.001.

PLS-PM identified direct and indirect effects of forest thinning, plant productivity, litter biomass, soil nutrient and plant nutrient on the ecosystem N, P, and K cycling functions (**Figures 5**–**7**). For N, P, and K cycling functions, thinning positively affected the plant productivity (0.84 of the direct effects) and negatively affected litter biomass (-0.76 of the direct effects) (**Figures 5**-**7A**). For N, and P cycling functions, plant productivity further negatively regulated plant nutrient and soil nutrient (-0.51 and -0.53 of the direct effects), and litter biomass positively regulated soil nutrient (0.33 of the direct effects) (**Figures 5**, **6A**). Plant and soil nutrients jointly exerted a negative direct effect on the ecosystem N (-0.28 and -0.74) and P (-0.35 and -0.68) cycling function (**Figures 5**, **6A**). For K cycling function, plant productivity directly positively regulated plant nutrient (0.51) and negatively regulated soil nutrient (-0.64) (**Figure 7A**). And plant nutrient and soil nutrient have a direct positive effect (0.56) and a direct negative effect (-0.52) on the ecosystem K cycling function (**Figure 7A**). Overall, the total positive effects of forest thinning on ecosystem N, P, and K cycling functions were 0.639, 0.626, and 0.585 respectively (**Figures 5**-**7B**).

## 4 Discussion

### 4.1 Differences in ecosystem N, P, and K cycling characteristics

Nutrient cycling is a key ecosystem process closely related to forest ecosystem structure and function and is also the basis for supporting organic matter production in forest ecosystems (Vrignon-Brenas et al., 2019; Perron et al., 2021). Although ecosystem nutrient cycling is crucial to the healthy and stable development of communities, there have been few evaluations of nutrient cycling characteristics at the ecosystem level. In our study, the ecosystem N, P, and K cycling parameters were significantly different from each other, which was consistent with previous research results on the key cycling processes of different nutrient elements (absorption, accumulation, distribution, utilization efficiency, etc.) (Rodríguez-Soalleiro et al., 2018; Schlesinger, 2021). A possible explanation is that the cycling process of different nutrient elements will be corporately affected by the physical and chemical properties of the elements, biological effects, environmental conditions (Vitousek, 2018; Johnson and Turner, 2019; Sayer et al., 2020), exhibiting different nutrient cycling characteristics. Specifically, the ecosystem’s high N return, absorption, and utilization rates, along with low N surplus, indicate severe nitrogen limitation for plant growth in this area, which conformed with our previous results regarding nutrient stoichiometry and nutrient reabsorption in multi-plant organs (Pang et al., 2021b). Additionally, compared with N and K, the ecosystem’s higher P productivity, retention coefficient, and plant nutrient turnover time and lower P cycle coefficient, recycling coefficient, and plant nutrient accumulation coefficient implied that although P productivity is high in this study area, the cycling intensity is the lowest. On the one hand, ecosystem P is mainly derived from the weathering of soil phosphate minerals, which is a relatively slow process (Zhou et al., 2018; Koester et al., 2021). On the other hand, ecosystem P cycle also depends on the amount of P returned from plant organisms (Whitehead, 2000; Mengel and Kirkby, 2012), but in this study, plant P showed higher retention and higher turnover time, which also led to lower ecosystem P cycling intensity. As for the N cycle, ecosystem N sources are diversified. In addition to soil N nutrient mineralization and plant return, N can also be input through atmospheric nitrogen deposition and biological nitrogen fixation, so it has a higher cycling intensity relative to P (Wang et al., 2007; Nevison et al., 2022). Unlike N and P, which often form complex molecular structures, K mainly exists as soluble ions. These ions are more easily leached, resulting in a faster nutrient cycle (Qiao et al., 2023). Moreover, in order to maintain the K balance, plants in the forest ecosystem have a “pumping” mechanism, which further promotes the K circulation rate in the ecosystem (Sardans and Peñuelas, 2015; Schlesinger, 2021). Overall, ecosystem N, P, and K cycles exhibited heterogeneous patterns, supporting our hypothesis 1.

### 4.2 Ecosystem N, P and K cycling functions following thinning

To maintain the forest ecosystem sustainable development, in addition to focusing on forest productivity sustainability, the importance of nutrient sustainability cannot be ignored (Haberl et al., 2010; Rodríguez-Soalleiro et al., 2018). It is well-known that the whole-tree harvesting method permanently removes a large amount of wood and plant residues from the nutrient cycling system of plant growth, and this high nutrient output rate has a negative impact on the ecosystem nutrient budget (Smolander et al., 2010; Thiffault et al., 2011; Pang et al., 2021b). Therefore, understanding the effects of whole-tree harvesting on the forest ecosystem nutrient cycling characteristics and functions at the ecosystem level is of great significance for explaining the forest ecosystem nutrient balance mechanism after thinning and formulating scientific forest management strategies (Jordan, 1985; Pei et al., 2017). Contrary to our initial hypothesis 2, whole-tree harvesting can significantly enhance the ecosystem N, P, and K cycling functions. The results of this study were inconsistent with previous researchers who suggested that whole-tree harvesting aggravated nutrient loss and had a negative impact on nutrient cycling in forest ecosystems (Kaarakka et al., 2014). From the details, there was no difference in the nutrient concentration and returned biomass of above-ground litter and underground fine roots under different thinning intensities (**Supplementary Figures S8**, **S17**, **S19**; **Supplementary Table S3**), and the nutrient input from atmospheric precipitation was also consistent. So, the total nutrient return amounts and nutrient productivity did not respond to thinning treatments, and had no effects on the ecosystem nutrient cycling functions (**Figure 1**). However, N, P, and K cycle coefficients, recycling coefficients, surplus rates, and plant nutrient accumulation coefficients significantly increased with the increasing thinning intensity, while N, P, and K absorption amounts, retention coefficients, and plant nutrient turnover time significantly decreased (**Figure 1**). Collectively, these results proved that the N, P and K cycling intensity in the ecosystem increased following thinning, promoting the improvement of N, P and K cycling functions at the ecosystem level. Simultaneously, there are coupling effects and mutual influences in the multi-nutrient element cycling process (Zhang et al., 2019). In this study area, due to N limitation, the ecosystem N absorption coefficient and utilization coefficient increased significantly with the increased thinning intensity, and the N surplus amounts showed a significantly low value at T2, indicating that the ecosystem N utilization and consumption increased significantly, the N cycling speed was accelerated, and which may also have led to the improvement of the ecosystem P and K cycling functions. Furthermore, previous studies on the effects of whole-tree harvesting on nutrient cycling in forest ecosystems mostly focused on the nutrient budget of plant or soil pools (the single ecosystem component) (Yan et al., 2017; Pang et al., 2021b), and lacked understanding of the nutrient cycling function at the ecosystem level. In this study, the ecosystem function index was employed to systematically extract comprehensive information regarding ecosystem nutrient cycling characteristics, providing in a more representative assessment of the impact of whole-tree harvesting on nutrient cycling functions at the ecosystem level.

### 4.3 Control factors of N, P and K cycling functions following thinning

Nutrient cycling in forest ecosystems involves complex processes driven by various influencing factors (Johnson and Lindberg, 2013; Vitousek, 2018). Whole-tree harvesting drastically changes the biotic and abiotic factors in the forest ecosystem, thereby most likely significantly affecting the ecosystem nutrient cycling function (McDaniel et al., 2013; Sicard et al., 2016). In the present study, PLS-PM revealed that whole-tree harvesting mainly promoted ecosystem N and P cycling functions through two pathways: (1) whole-tree harvesting regulated litter biomass and affected soil nutrient characteristics, then regulating the ecosystem N and P cycling functions; (2) whole-tree harvesting adjusted ecosystem plant productivity, influenced both plant and soil nutrient characteristics, and then adjusting ecosystem N and P cycling functions. First of all, early studies pointed out that litter nutrient return is an important source of soil nutrients (Chao et al., 2019; Keller and Phillips, 2019), and litter biomass has a positive impact on soil nutrients (**Figures 5**, **6**). Our results showed that whole-tree harvesting significantly reduced litter biomass return and had a negative impact on soil nutrients (**Supplementary Figure S3**; **Supplementary Tables S2**, **S3**), in agreement with previous results (Clarke et al., 2021; Smith et al., 2022; Vos et al., 2023). Secondly, the ecosystem plant productivity represents the ability of plant systems to compete and consume nutrients (Liu, 2009). We found that although the productivity of the tree layer was significantly reduced by whole-tree harvesting, the productivity of the understory vegetation increased rapidly after thinning (**Supplementary Figure S6**). At the same time, the VPA analysis results showed that the ecosystem plant productivity was the main controlling factor of the ecosystem N and P cycling functions while the PLS-PM path coefficient showed that the whole-tree harvesting had a significant positive effect on the ecosystem plant productivity. All these results indicated that the productivity of understory vegetation after thinning played a more significant role in regulating the ecosystem N and P cycling functions and confirmed findings from previous studies that thinning promotes understory vegetation growth, with its productivity making an essential contribution to ecosystem nutrient reserves and nutrient cycling (Ma et al., 2007). Therefore, when whole-tree harvesting caused a large amount of nutrient loss, the rapidly increased understory vegetation productivity intensified nutrient consumption and competition, having negative effects on plant and soil nutrient characteristics (Elser et al., 1996; Bauhus et al., 2001). Ultimately, the degradation of plant and soil nutrient characteristics had direct negative impacts on the ecosystem N and P cycling functions, supporting the previous conclusion that intensity disturbance negatively affects the nutrient budget and nutrient cycling function (Kaarakka et al., 2014).

Compared with the ecosystem N and P cycling functions, PLS-PM results showed that whole-tree harvesting mainly affected the plant and soil nutrient characteristics by adjusting the ecosystem plant productivity, thereby enhancing the ecosystem K cycling function. Moreover, within the regulation pathway, it was noteworthy that the positive effect of ecosystem plant productivity on plant nutrient characteristics, as well as the positive effect of plant nutrient characteristics on K cycling function, differed from the effects of plant productivity on N and P cycling functions, where it exerts an indirect positive effect through its negative impact on plant nutrient characteristics. It is well known that removing trees through thinning can reduce competition pressure for nutrient resources in plant communities (Hertel et al., 2009). Relative to the important regulatory role of herb nutrients (plant nutrient characteristics) on the ecosystem N and P cycling functions, tree nutrients were included in the plant nutrient characteristics in the K cycling function PLS-PM model (**Figures 5**-**7C**). Therefore, in the K-cycling process, the direct positive effects of ecosystem plant productivity on plant nutrient characteristics (herbs and trees) may be due to the fact that the nutrient competition pressure released by thinning exceeded the nutrient consumption and competition pressure caused by increased understory vegetation productivity, thus inducing the positive effects of ecosystem plant productivity on plant nutrient characteristics. Additionally, the differential regulation patterns of N, P, and K cycling functions may depend on synergistic effects of multiple factors not monitored in this study, such as climate drivers, elemental heterogeneity or biocatalytic effects (Vitousek, 2018; Johnson and Turner, 2019; Sayer et al., 2020). And unexplained parts of the VPA and PLS-PM models require further investigation (**Figures 6**, **7**). Furthermore, the direct positive effect of plant nutrient characteristics on K cycling function was consistent with the positive correlation between plant nutrient stoichiometric ratios and K cycling function index (**Figures 3A**, **7A**), suggesting the importance of plant nutrient stoichiometric ratios in the ecosystem K cycling function regulation. Similar findings were also reported elsewhere in studies of forest or non-forest ecosystem nutrient cycling (Tripler et al., 2006; Chen B. et al., 2023). Overall, whole-tree harvesting showed inconsistent regulatory mechanisms for N, P and K cycling functions, supporting our hypothesis 3.

## 5 Conclusions

Our study proved that the ecosystem N, P, and K cycles had heterogeneous cycling patterns. Whole-tree harvesting significantly improved the ecosystem N, P and K cycling functions. We found two regulatory paths for N and P cycling functions: (a) whole-tree harvesting regulated litter biomass and affected soil nutrient characteristics, then regulating the ecosystem N and P cycling functions, (b) whole-tree harvesting adjusted plant productivity, influenced both plant and soil nutrient characteristics, and then thereby adjusting ecosystem N and P cycling functions. Contrastively, whole-tree harvesting mainly affected the plant and soil nutrient characteristics by adjusting the ecosystem plant productivity, and promoting the ecosystem K cycling function. N and P cycling functions were mainly regulated by understory plant productivity while tree and herb nutrient characteristics were key driving factors for K cycling functions. These results indicate that the ecosystem N, P, and K cycling functions are driven by different mechanisms. We believed that this work could advance our understanding of the effects of forest management on the forest ecosystem nutrient cycle.

## Data Availability

The original contributions presented in the study are included in the article/**Supplementary Material**. Further inquiries can be directed to the corresponding author.

## References

[B1] Bao S. (2000). Soil and agricultural chemistry analysis (Beijing: China agriculture press).

[B2] Bauhus J. Aubin I. Messier C. Connell M. (2001). Composition, structure, light attenuation and nutrient content of the understorey vegetation in a *Eucalyptus sieberi* regrowth stand 6 years after thinning and fertilisation. For. Ecol. Manage. 144, 275–286. doi: 10.1016/S0378-1127(00)00403-5

[B3] Brassard B. W. Chen H. Y. Cavard X. Laganière J. Reich P. B. Bergeron Y.. (2013). Tree species diversity increases fine root productivity through increased soil volume filling. J. Ecol. 101, 210–219. doi: 10.1111/1365-2745.12023

[B4] Chao L. Liu Y. Freschet G. T. Zhang W. Yu X. Zheng W.. (2019). Litter carbon and nutrient chemistry control the magnitude of soil priming effect. Funct. Ecol. 33, 876–888. doi: 10.1111/1365-2435.13278

[B5] Chen B. Fang J. Piao S. Ciais P. Black T. A. Wang F.. (2023). A meta-analysis highlights globally widespread potassium limitation in terrestrial ecosystems. New Phytol. 241, 154–165. doi: 10.1111/nph.19294 37804058

[B6] Chen C. Weng Y. Huang K. Chen X. Li H. Tang Y.. (2023). Decomposition of harvest residues and soil chemical properties in a *Eucalyptus urophylla× grandis* plantation under different residue management practices in southern China. For. Ecol. Manage. 529, 120756. doi: 10.1016/j.foreco.2022.120756

[B7] Chen X. Chen H. Y. Chen X. Wang J. Chen B. Wang D.. (2016). Soil labile organic carbon and carbon-cycle enzyme activities under different thinning intensities in Chinese fir plantations. Appl. Soil Ecol. 107, 162–169. doi: 10.1016/j.apsoil.2016.05.016

[B8] Clarke N. Kiær L. P. Kjønaas O. J. Bárcena T. G. Vesterdal L. Stupak I.. (2021). Effects of intensive biomass harvesting on forest soils in the Nordic countries and the UK: A meta-analysis. For. Ecol. Manage. 482, 118877. doi: 10.1016/j.foreco.2020.118877

[B9] De Vos B. Van Meirvenne M. Quataert P. Deckers J. Muys B. (2005). Predictive quality of pedotransfer functions for estimating bulk density of forest soils. Soil Soil Science. Soc. America J. 69, 500–510. doi: 10.2136/sssaj2005.0500

[B10] Elser J. J. Dobberfuhl D. R. MacKay N. A. Schampel J. H. (1996). Organism size, life history, and N: P stoichiometry: toward a unified view of cellular and ecosystem processes. BioScience 46, 674–684. doi: 10.2307/1312897

[B11] Garrett L. G. Smaill S. J. Beets P. N. Kimberley M. O. Clinton P. W. (2021). Impacts of forest harvest removal and fertiliser additions on end of rotation biomass, carbon and nutrient stocks of *Pinus radiata* . For. Ecol. Manage. 493, 119161. doi: 10.1016/j.foreco.2021.119161

[B12] Gérard F. Mayer K. Hodson M. Ranger J. (2008). Modelling the biogeochemical cycle of silicon in soils: application to a temperate forest ecosystem. Geochim. Cosmochim. Acta 72, 741–758. doi: 10.1016/j.gca.2007.11.010

[B13] Haberl H. Beringer T. Bhattacharya S. C. Erb K.-H. Hoogwijk M. (2010). The global technical potential of bio-energy in 2050 considering sustainability constraints. Curr. Opin. Environ. Sustain. 2, 394–403. doi: 10.1016/j.cosust.2010.10.007 24069093 PMC3778854

[B14] Hector A. Bagchi R. (2007). Biodiversity and ecosystem multifunctionality. Nature 448, 188–190. doi: 10.1038/nature05947 17625564

[B15] Hertel D. Harteveld M. A. Leuschner C. (2009). Conversion of a tropical forest into agroforest alters the fine root-related carbon flux to the soil. Soil Biol. Biochem. 41, 481–490. doi: 10.1016/j.soilbio.2008.11.020

[B16] Hou S. L. Hättenschwiler S. Yang J. J. Sistla S. Wei H. W. Zhang Z. W.. (2021). Increasing rates of long-term nitrogen deposition consistently increased litter decomposition in a semi-arid grassland. New Phytol. 229, 296–307. doi: 10.1111/nph.16854 32762047

[B17] Jia R. Zhou J. Chu J. Shahbaz M. Yang Y. Jones D. L.. (2022). Insights into the associations between soil quality and ecosystem multifunctionality driven by fertilization management: A case study from the North China Plain. J. Clean. Prod. 362, 132265. doi: 10.1016/j.jclepro.2022.132265

[B18] Johnson D. W. Lindberg S. E. (2013). Atmospheric deposition and forest nutrient cycling: a synthesis of the integrated forest study (Springer Science & Business Media).

[B19] Johnson D. W. Turner J. (2019). Tamm Review: Nutrient cycling in forests: A historical look and newer developments. For. Ecol. Manage. 444, 344–373. doi: 10.1016/j.foreco.2019.04.052

[B20] Jordan C. F. (1985). Nutrient cycling in tropical forest ecosystems. Principles and their application in management and conservation (John Wiley & Sons).

[B21] Kaarakka L. Tamminen P. Saarsalmi A. Kukkola M. Helmisaari H.-S. Burton A. J. (2014). Effects of repeated whole-tree harvesting on soil properties and tree growth in a Norway spruce (*Picea abies (L.) Karst.*) stand. For. Ecol. Manage. 313, 180–187. doi: 10.1016/j.foreco.2013.11.009

[B22] Kang H. Xue Y. Yan C. Lu S. Yang H. Zhu J.. (2022). Contrasting patterns of microbial nutrient limitations between rhizosphere and bulk soil during stump sprout restoration in a clear-cut oak forest. For. Ecol. Manage. 515, 120241. doi: 10.1016/j.foreco.2022.120241

[B23] Keller A. B. Phillips R. P. (2019). Leaf litter decay rates differ between mycorrhizal groups in temperate, but not tropical, forests. New Phytol. 222, 556–564. doi: 10.1111/nph.15524 30299541

[B24] Kim S. Li G. Han S. H. Kim H.-J. Kim C. Lee S.-T.. (2018). Thinning affects microbial biomass without changing enzyme activity in the soil of *Pinus densiflora* Sieb. et Zucc. forests after 7 years. Ann. For. Sci. 75, 1–10. doi: 10.1007/s13595-018-0690-1

[B25] Koester M. Stock S. C. Nájera F. Abdallah K. Gorbushina A. Prietzel J.. (2021). From rock eating to vegetarian ecosystems—Disentangling processes of phosphorus acquisition across biomes. Geoderma 388, 114827. doi: 10.1016/j.geoderma.2020.114827

[B26] Legout A. Hansson K. van der Heijden G. Laclau J.-P. Mareschal L. Nys C.. (2020). Chemical fertility of forest ecosystems. Part 2: Towards redefining the concept by untangling the role of the different components of biogeochemical cycling. For. Ecol. Manage. 461, 117844. doi: 10.1016/j.foreco.2019.117844

[B27] Li M. (1997). Nutrient dynamics of a Futian mangrove forest in Shenzhen, South China. Estuar. Coast. Shelf Sci. 45, 463–472. doi: 10.1006/ecss.1996.0201

[B28] Li W. (2017). Comparative analysis of main stands’s effects on the water quality in the Houditang forest region, Qinling Mountains (Northwest Agriculture and Forestry University).

[B29] Li X. Li Y. Zhang J. Peng S. Chen Y. Cao Y. (2020). The effects of forest thinning on understory diversity in China: A meta-analysis. Land Degrad. Dev. 31, 1225–1240. doi: 10.1002/ldr.3540

[B30] Liu Z. (2009). Material Accumulation and Cycling in Forest Ecosystem (Beijing: China Forestry Press).

[B31] Ma J. Kang F. Cheng X. Han H. (2018). Moderate thinning increases soil organic carbon in *Larix principis-rupprechtii (Pinaceae)* plantations. Geoderma 329, 118–128. doi: 10.1016/j.geoderma.2018.05.021

[B32] Ma X. Heal K. V. Liu A. Jarvis P. G. (2007). Nutrient cycling and distribution in different-aged plantations of Chinese fir in southern China. For. Ecol. Manage. 243, 61–74. doi: 10.1016/j.foreco.2007.02.018

[B33] McDaniel M. Kaye J. Kaye M. (2013). Increased temperature and precipitation had limited effects on soil extracellular enzyme activities in a post-harvest forest. Soil Biol. Biochem. 56, 90–98. doi: 10.1016/j.soilbio.2012.02.026

[B34] Mengel K. Kirkby E. A. (2012). Principles of plant nutrition (Springer Science & Business Media).

[B35] Moi D. A. Lansac-Tôha F. M. Romero G. Q. Sobral-Souza T. Cardinale B. J. Kratina P.. (2022). Human pressure drives biodiversity–multifunctionality relationships in large Neotropical wetlands. Nat. Ecol. Evol. 6, 1279–1289. doi: 10.1038/s41559-022-01827-7 35927315

[B36] Nevison C. Hess P. Goodale C. Zhu Q. Vira J. (2022). Nitrification, denitrification, and competition for soil N: Evaluation of two earth system models against observations. Ecol. Appl. 32, e2528. doi: 10.1002/eap.2528 35019177

[B37] Pang Y. Tian J. Liu L. Han L. Wang D. (2021a). Coupling of different plant functional group, soil, and litter nutrients in a natural secondary mixed forest in the Qinling Mountains, China. Environ. Sci. pollut. Res. 28, 66272–66286. doi: 10.1007/s11356-021-15632-5 34333746

[B38] Pang Y. Tian J. Lv X. Wang R. Wang D. Zhang F. (2022). Contrasting dynamics and factor controls in leaf compared with different-diameter fine root litter decomposition in secondary forests in the Qinling Mountains after 5 years of whole-tree harvesting. Sci. Total Environ. 838, 156194. doi: 10.1016/j.scitotenv.2022.156194 35618114

[B39] Pang Y. Tian J. Wang D. (2021b). Response of multi-ecological component stoichiometry and tree nutrient resorption to medium-term whole-tree harvesting in secondary forests in the Qinling Mountains, China. For. Ecol. Manage. 498, 119573. doi: 10.1016/j.foreco.2021.119573

[B40] Pei Z. Leppert K. N. Eichenberg D. Bruelheide H. Niklaus P. A. Buscot F.. (2017). Leaf litter diversity alters microbial activity, microbial abundances, and nutrient cycling in a subtropical forest ecosystem. Biogeochemistry 134, 163–181. doi: 10.1007/s10533-017-0353-6

[B41] Perron T. Mareschal L. Laclau J.-P. Deffontaines L. Deleporte P. Masson A.. (2021). Dynamics of biomass and nutrient accumulation in rubber (*Hevea brasiliensis*) plantations established on two soil types: Implications for nutrient management over the immature phase. Ind. Crops Prod. 159, 113084. doi: 10.1016/j.indcrop.2020.113084

[B42] Qi D. Feng F. Lu C. Fu Y. (2022). C: N: P stoichiometry of different soil components after the transition of temperate primary coniferous and broad-leaved mixed forests to secondary forests. Soil Tillage Res. 216, 105260. doi: 10.1016/j.still.2021.105260

[B43] Qiao P. Wang S. Li J. Zhao Q. Wei Y. Lei M.. (2023). Process, influencing factors, and simulation of the lateral transport of heavy metals in surface runoff in a mining area driven by rainfall: A review. Sci. Total Environ. 857, 159119. doi: 10.1016/j.scitotenv.2022.159119 36183764

[B44] Qiu X. Wang H. Peng D. Liu X. Yang F. Li Z.. (2020). Thinning drives C:N:P stoichiometry and nutrient resorption in *Larix principis-rupprechtii* plantations in North China. For. Ecol. Manage. 462, 117984. doi: 10.1016/j.foreco.2020.117984

[B45] R Development Core Team (2017). R: A Language and Environment for Statstical Computing. Version 3.3.2. R (Foundation for Statistical Computing). Available at: http://www.Rproject.org/.

[B46] Ren C. Zhang W. Zhong Z. Han X. Yang G. Feng Y.. (2018). Differential responses of soil microbial biomass, diversity, and compositions to altitudinal gradients depend on plant and soil characteristics. Sci. Total Environ. 610, 750–758. doi: 10.1016/j.scitotenv.2017.08.110 28822942

[B47] Rodríguez-Soalleiro R. Eimil-Fraga C. Gómez-García E. García-Villabrille J. D. Rojo-Alboreca A. Muñoz F.. (2018). Exploring the factors affecting carbon and nutrient concentrations in tree biomass components in natural forests, forest plantations and short rotation forestry. For. Ecosyst. 5, 1–18. doi: 10.1186/s40663-018-0154-y

[B48] Sardans J. Peñuelas J. (2015). Potassium: a neglected nutrient in global change. Global Ecol. Biogeogr. 24, 261–275. doi: 10.1111/geb.12259

[B49] Sayer E. J. Rodtassana C. Sheldrake M. Bréchet L. M. Ashford O. S. Lopez-Sangil L.. (2020). Revisiting nutrient cycling by litterfall—insights from 15 years of litter manipulation in old-growth lowland tropical forest. Adv. Ecol. Res. 62, 173–223. doi: 10.1016/bs.aecr.2020.01.002

[B50] Schlesinger W. H. (2021). Some thoughts on the biogeochemical cycling of potassium in terrestrial ecosystems. Biogeochemistry 154, 427–432. doi: 10.1007/s10533-020-00704-4

[B51] Sicard P. Augustaitis A. Belyazid S. Calfapietra C. de Marco A. Fenn M.. (2016). Global topics and novel approaches in the study of air pollution, climate change and forest ecosystems. Environ. pollut. 213, 977–987. doi: 10.1016/j.envpol.2016.01.075 26873061

[B52] Smith C. T. Briggs R. D. Stupak I. Preece C. Rezai-Stevens A. Barusco B.. (2022). Effects of whole-tree and stem-only clearcutting on forest floor and soil carbon and nutrients in a balsam fir (*Abies balsamea* (L.) Mill.) and red spruce (*Picea rubens* Sarg.) dominated ecosystem. For. Ecol. Manage. 519, 120325. doi: 10.1016/j.foreco.2022.120325

[B53] Smolander A. Kitunen V. Tamminen P. Kukkola M. (2010). Removal of logging residue in Norway spruce thinning stands: long-term changes in organic layer properties. Soil Biol. Biochem. 42, 1222–1228. doi: 10.1016/j.soilbio.2010.04.015

[B54] Tamm C. O. (2012). Nitrogen in terrestrial ecosystems: questions of productivity, vegetational changes, and ecosystem stability (Springer Science & Business Media).

[B55] Thiffault E. Hannam K. D. Paré D. Titus B. D. Hazlett P. W. Maynard D. G.. (2011). Effects of forest biomass harvesting on soil productivity in boreal and temperate forests—A review. Environ. Rev. 19, 278–309. doi: 10.1139/a11-009

[B56] Tripler C. E. Kaushal S. S. Likens G. E. Todd Walter M. (2006). Patterns in potassium dynamics in forest ecosystems. Ecol. Lett. 9, 451–466. doi: 10.1111/j.1461-0248.2006.00891.x 16623731

[B57] Turner J. Lambert M. (2015). Analysis of nutrient use efficiency (NUE) in *Eucalyptus pilularis* forests. Aust. J. Bot. 62, 558–569. doi: 10.1071/BT14162

[B58] Vitousek P. M. (2018). Nutrient cycling and limitation (Princeton University Press).

[B59] Vos M. A. den Ouden J. Hoosbeek M. Valtera M. de Vries W. Sterck F. (2023). The sustainability of timber and biomass harvest in perspective of forest nutrient uptake and nutrient stocks. For. Ecol. Manage. 530, 120791. doi: 10.1016/j.foreco.2023.120791

[B60] Vrignon-Brenas S. Gay F. Ricard S. Snoeck D. Perron T. Mareschal L.. (2019). Nutrient management of immature rubber plantations. A review. Agron. Sustain. Dev. 39, 11. doi: 10.1007/s13593-019-0554-6

[B61] Wang Y. P. Houlton B. Field C. (2007). A model of biogeochemical cycles of carbon, nitrogen, and phosphorus including symbiotic nitrogen fixation and phosphatase production. Global Biogeochem. Cycles 21, 1. doi: 10.1029/2006GB002797

[B62] Whitehead D. C. (2000). Nutrient elements in grassland: soil-plant-animal relationships (Cabi).

[B63] Yan T. Zhu J. Song H. Yang K. (2019). Resorption-related nitrogen changes in the leaves and roots of *Larix kaempferi* seedlings under nutrient-sufficient and nutrient-starvation conditions. J. Plant Ecol. 12, 615–623. doi: 10.1093/jpe/rty056

[B64] Yan T. Zhu J. Yang K. Yu L. Zhang J. (2017). Nutrient removal under different harvesting scenarios for larch plantations in northeast China: Implications for nutrient conservation and management. For. Ecol. Manage. 400, 150–158. doi: 10.1016/j.foreco.2017.06.004

[B65] Yang Y. Chai Y. Xie H. Zhang L. Zhang Z. Yang X.. (2023). Responses of soil microbial diversity, network complexity and multifunctionality to three land-use changes. Sci. Total Environ. 859, 160255. doi: 10.1016/j.scitotenv.2022.160255 36402341

[B66] Yu F. Li C. Yuan Z. Luo Y. Yin Q. Wang Q.. (2023). How do mountain ecosystem services respond to changes in vegetation and climate? An evidence from the Qinling Mountains, China. Ecol. Indic. 154, 110922. doi: 10.1016/j.ecolind.2023.110922

[B67] Zhang J. Zhao N. Liu C. Yang H. Li M. Yu G.. (2018a). C:N:P stoichiometry in China’s forests: From organs to ecosystems. Funct. Ecol. 32, 50–60. doi: 10.1111/1365-2435.12979

[B68] Zhang S. (2005). Effects of forest ecosystem on runoff and water quality in medium-altitude mountainous region of southern slopes, Qinling Mountains (Northwest Agriculture and Forestry University).

[B69] Zhang S. Lei R. Lv Y. Ma Y. (2000). Water-balance of forest eco-system in Huoditang area of Qinling Mountain. Bull. Soil Water Conserv. 20, 18–22. doi: 10.13961/j.cnki.stbctb.2000.06.006

[B70] Zhang W. Liu W. Xu M. Deng J. Han X. Yang G.. (2019). Response of forest growth to C:N:P stoichiometry in plants and soils during Robinia pseudoacacia afforestation on the Loess Plateau, China. Geoderma 337, 280–289. doi: 10.1016/j.geoderma.2018.09.042

[B71] Zhang X. Guan D. Li W. Sun D. Jin C. Yuan F.. (2018b). The effects of forest thinning on soil carbon stocks and dynamics: A meta-analysis. For. Ecol. Manage. 429, 36–43. doi: 10.1016/j.foreco.2018.06.027

[B72] Zhao X. (2015). Research the effects of thinning intensities on water quality of *QUERCUS ALIENA VAR. ACUTESERRATA* forest on the south slope of Qinling Mountain (Northwest Agriculture and Forestry University).

[B73] Zhou J. Bing H. Wu Y. Sun H. Wang J. (2018). Weathering of primary mineral phosphate in the early stages of ecosystem development in the Hailuogou Glacier foreland chronosequence. Eur. J. Soil Sci. 69, 450–461. doi: 10.1111/ejss.12536

