# Supplementary material for: Whole-tree harvesting improves the ecosystem N, P and K cycling functions in secondary forests in the Qinling Mountains, China
Source: Front Plant Sci. 2024 Dec 20;15:1394112. doi: 10.3389/fpls.2024.1394112 (PMC11699539; doi:10.3389/fpls.2024.1394112)
Supplement: Supplementary file 1 [file Table1.docx]

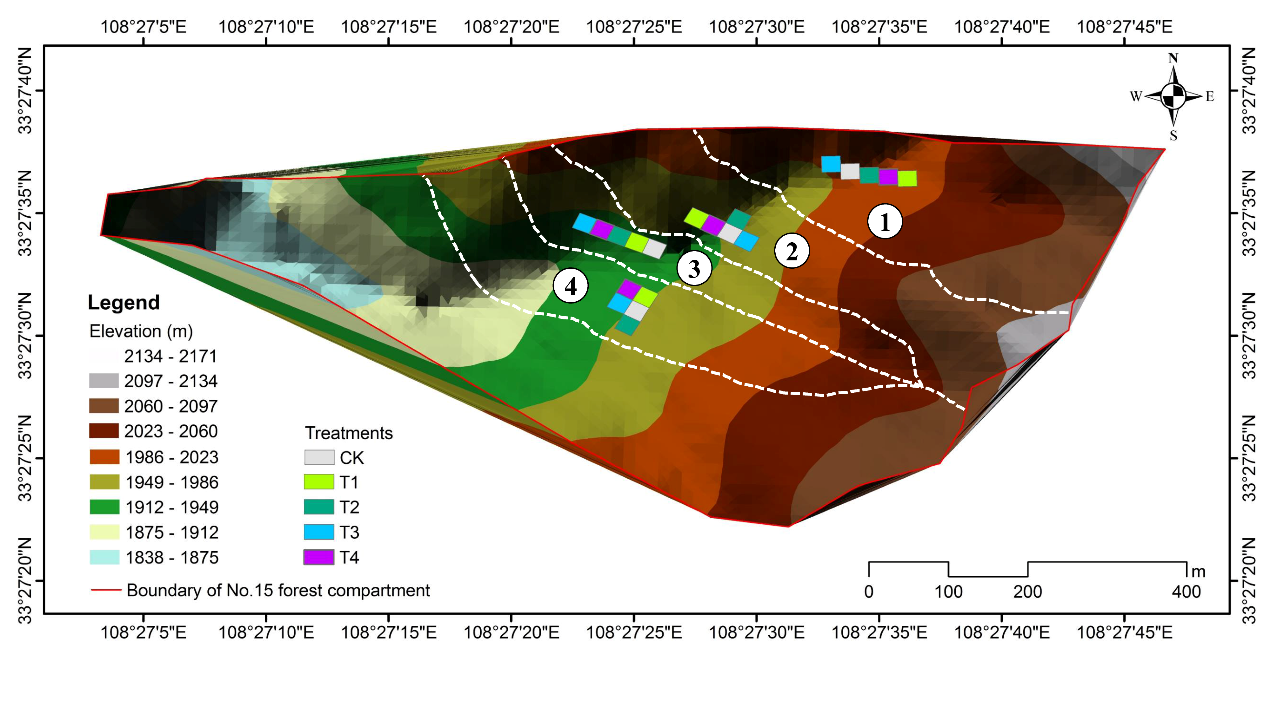


**Figure S1 The experimental design layout of sample plots.** CK, T1, T2, T3, T4 represent 0%, 15%, 30%, 45% and 60% thinning intensity, respectively. ①, ②, ③, ④ are the block numbers.


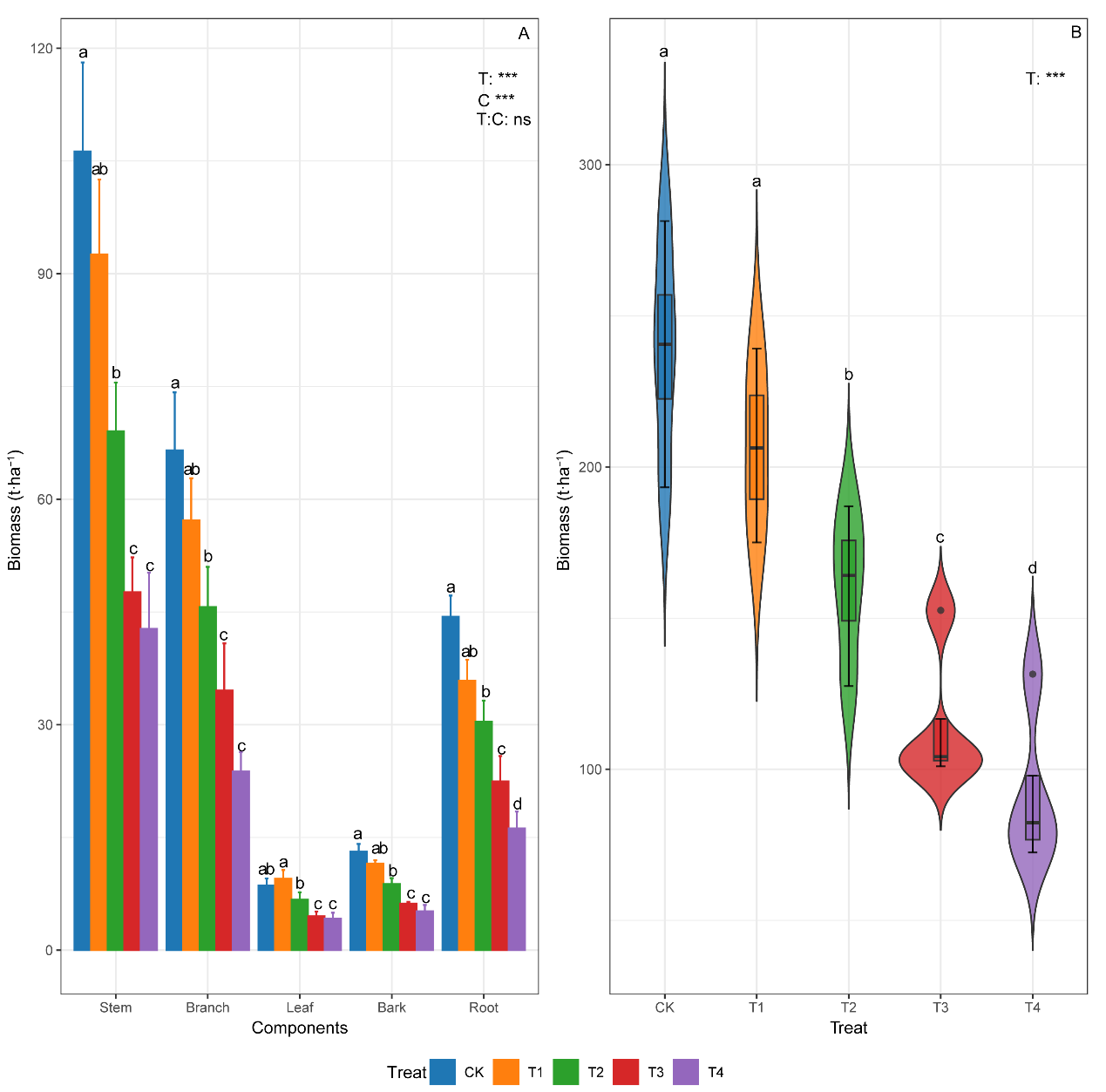


**Figure S2 Biomass of tree organs (A) and total (B) under different thinning intensities.** CK, T1, T2, T3, T4 represent 0%, 15%, 30%, 45% and 60% thinning intensity, respectively. T: treat, C: component, T:C: interaction. Different letters indicate significant differences between different thinning intensities. ns: non-significant, *** p < 0.001.


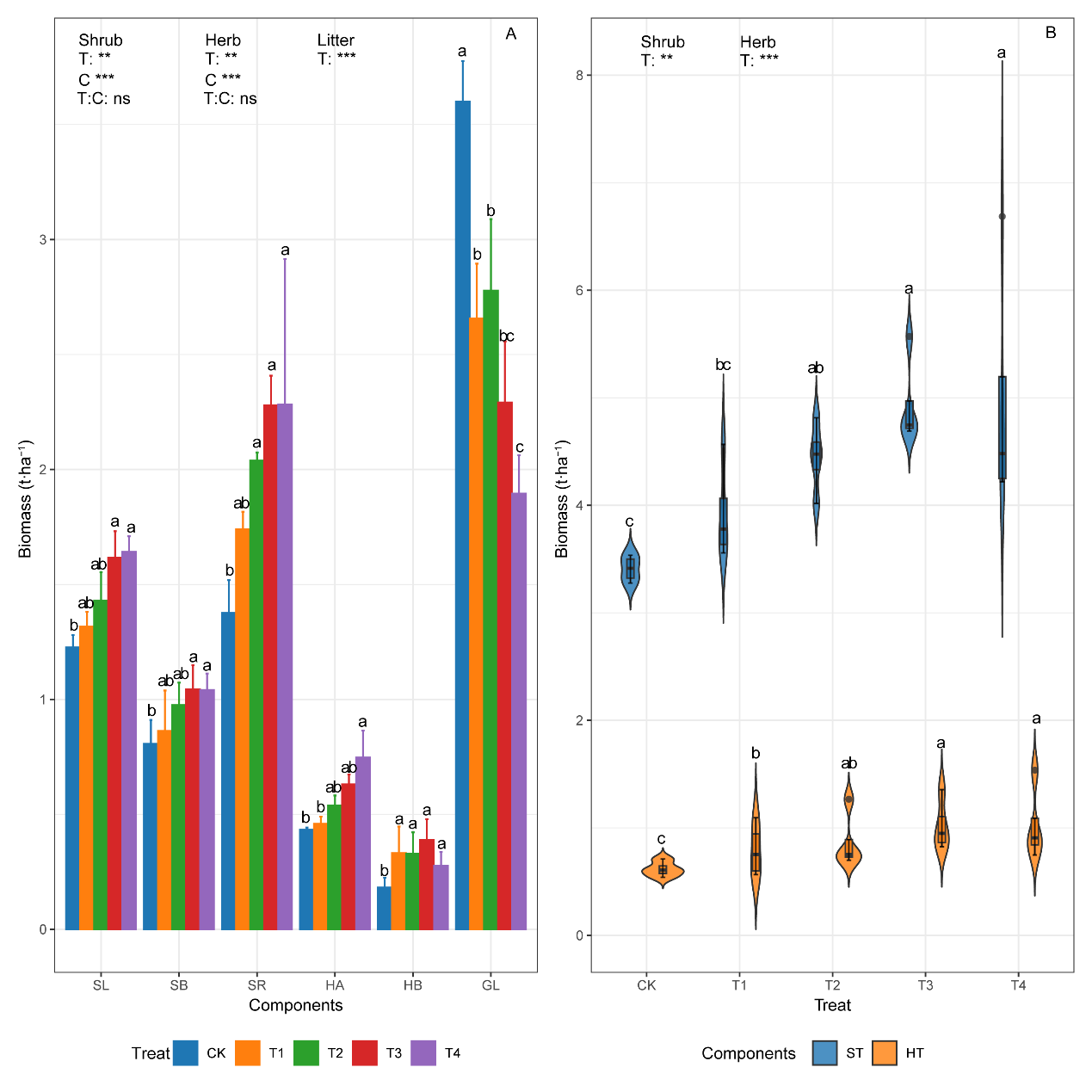


**Figure S3 Biomass of organs (A) and total (B) of shrub and herb, and litter biomass under different thinning intensities.** CK, T1, T2, T3, T4 represent 0%, 15%, 30%, 45% and 60% thinning intensity, respectively. T: treat, C: component, T:C: interaction. ST, HT: shrub and herb total attributes, SL, SB, SR: shrub leaf, branch and root, HA, HB: herb aboveground and underground, GL: ground litter. Different letters indicate significant differences between different thinning intensities. ns: non-significant, ** p < 0.01, *** p < 0.001.


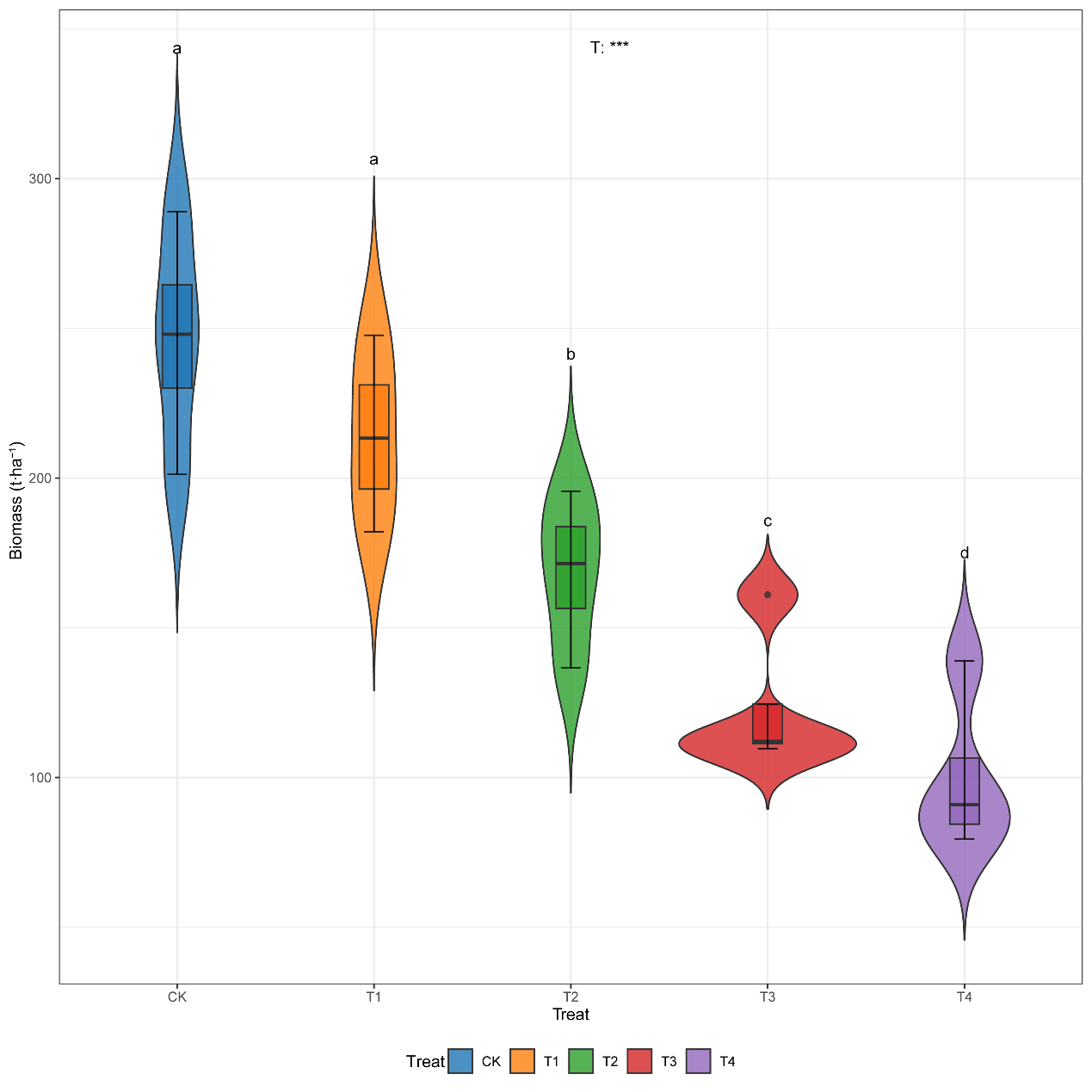


**Figure S4 Biomass of ecosystem under different thinning intensities.** CK, T1, T2, T3, T4 represent 0%, 15%, 30%, 45% and 60% thinning intensity, respectively. T: treat. Different letters indicate significant differences between different thinning intensities. *** p < 0.001.


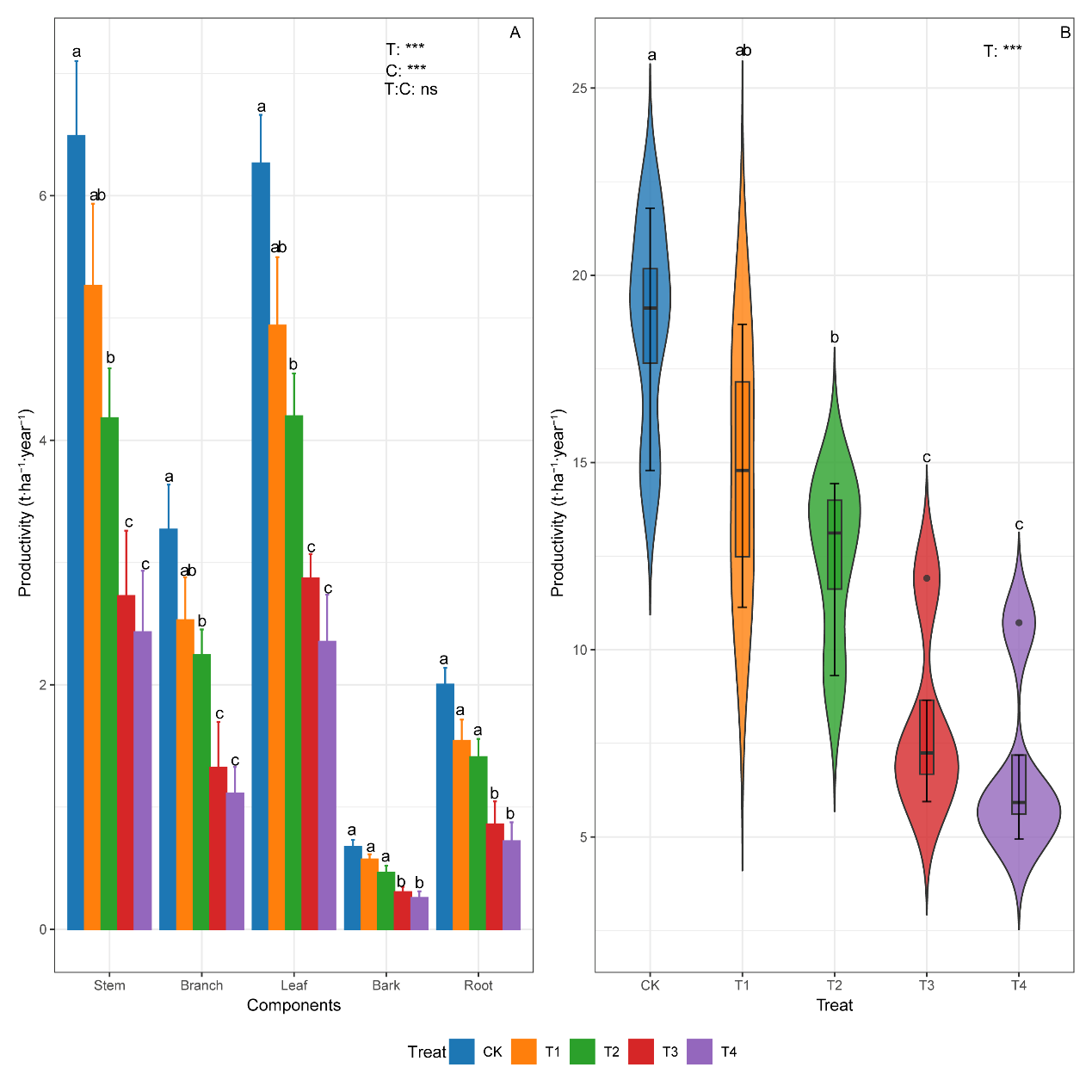


**Figure S5 Productivity of tree organs (A) and total (B) under different thinning intensities.** CK, T1, T2, T3, T4 represent 0%, 15%, 30%, 45% and 60% thinning intensity, respectively. T: treat, C: component, T:C: interaction. Different letters indicate significant differences between different thinning intensities. ns: non-significant, *** p < 0.001.


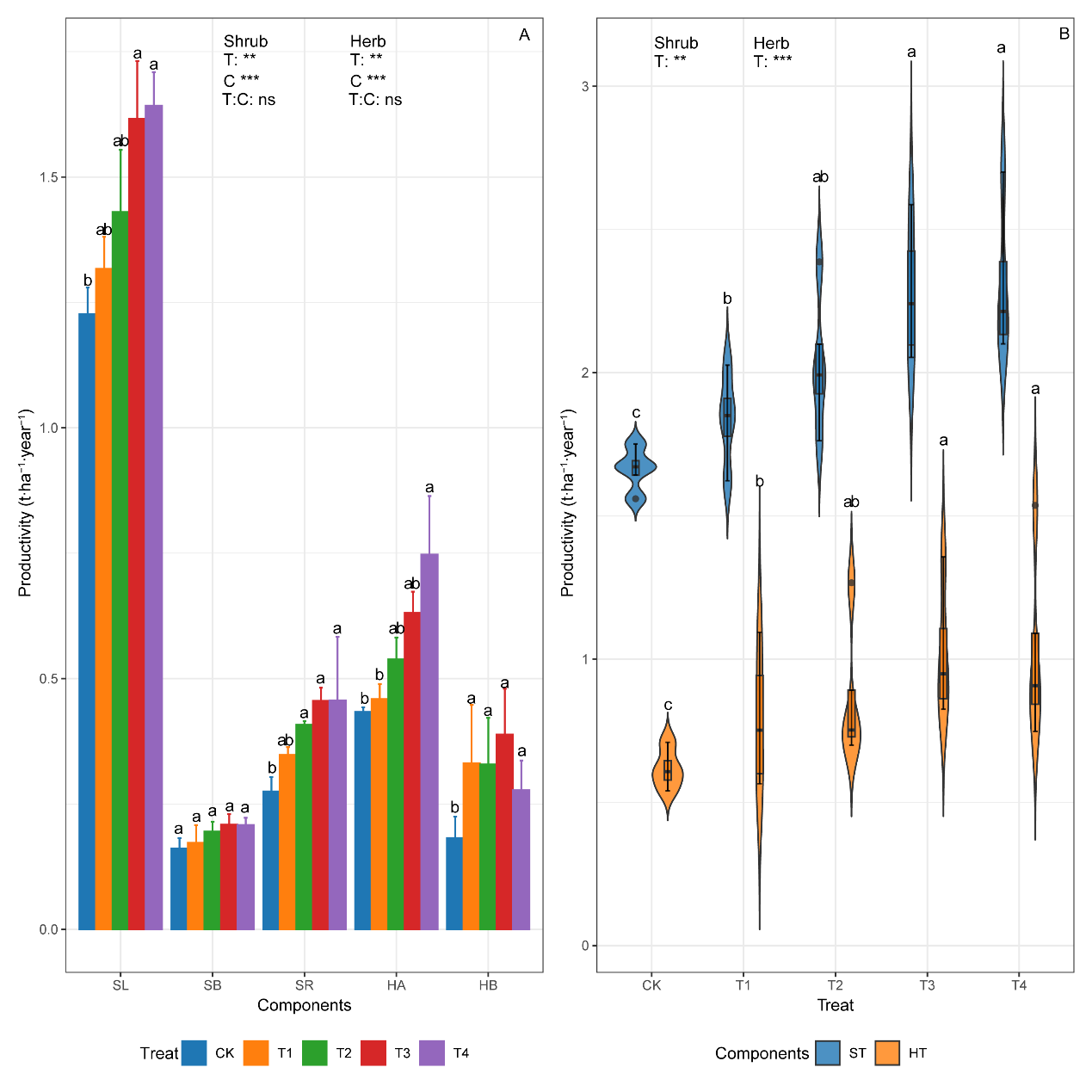


**Figure S6 Productivity of organs (A) and total (B) of shrub and herb under different thinning intensities.** CK, T1, T2, T3, T4 represent 0%, 15%, 30%, 45% and 60% thinning intensity, respectively. T: treat, C: component, T:C: interaction. ST, HT: shrub and herb total attributes, SL, SB, SR: shrub leaf, branch and root, HA, HB: herb aboveground and underground. Different letters indicate significant differences between different thinning intensities. ns: non-significant, ** p < 0.01, *** p < 0.001.


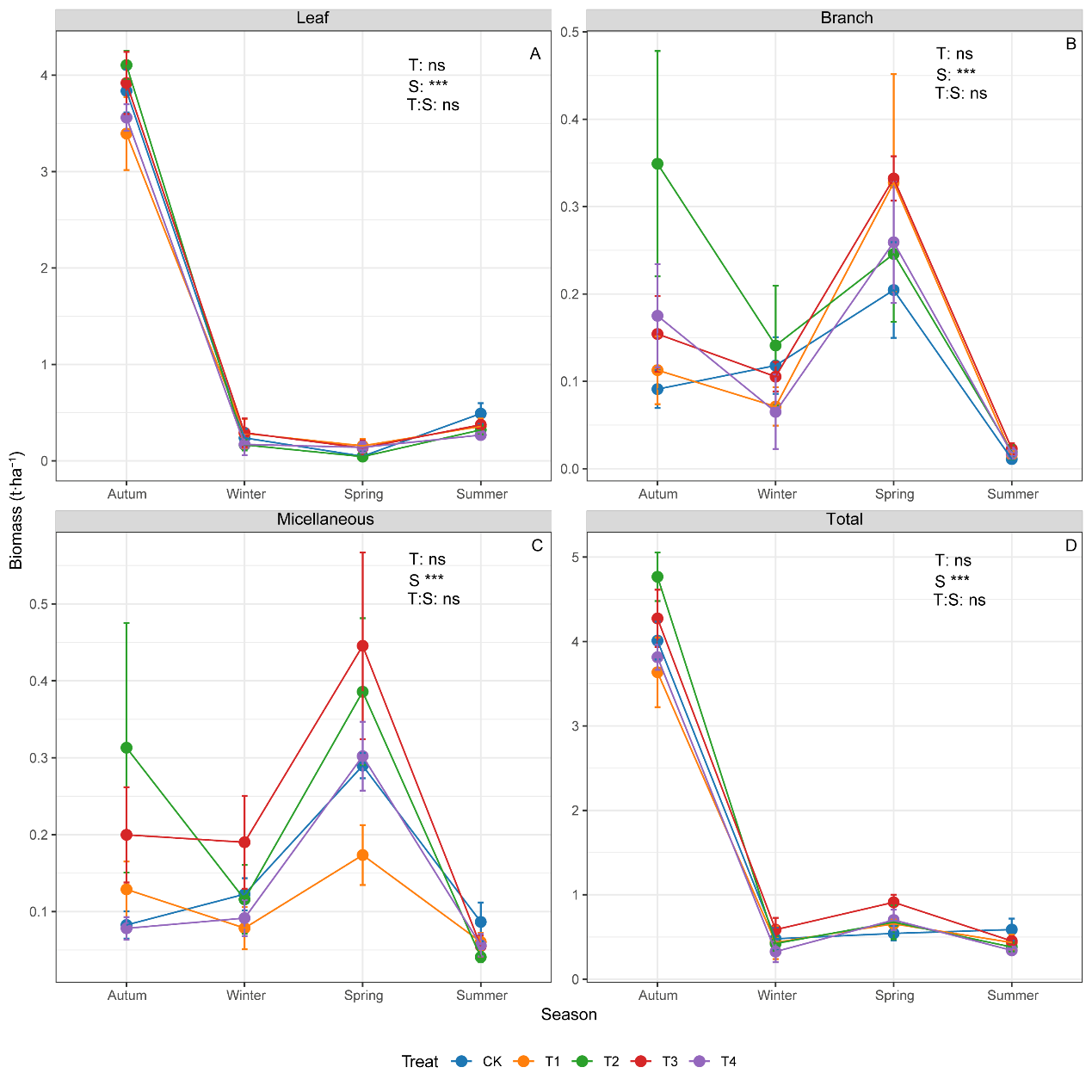


**Figure S7 Seasonal variation of litter components (A, B and C) and total (D) biomass (i.e. productivity) in one year.** T: treat, S: season, T:S: interaction. ns: non-significant, *** p < 0.001.


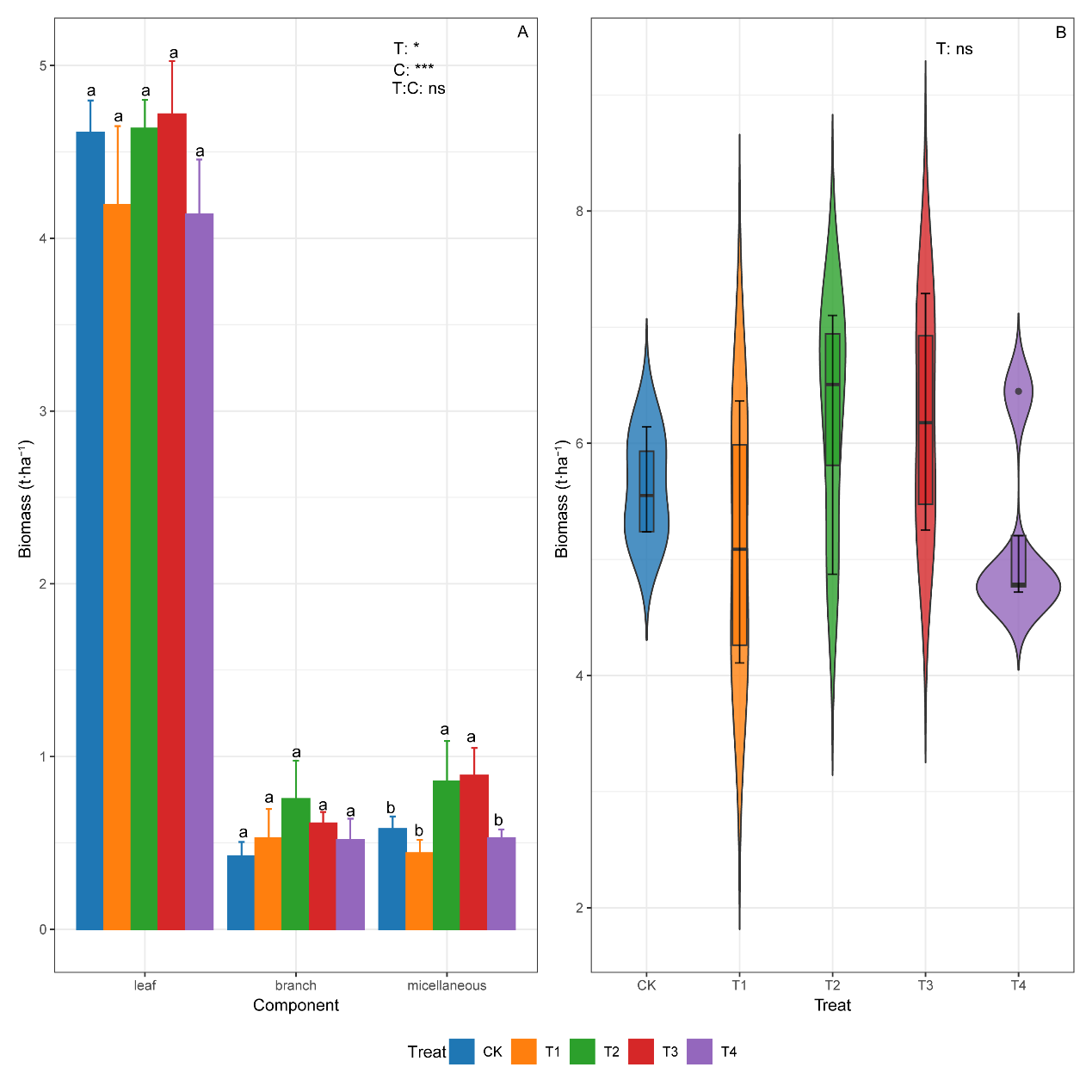


**Figure S8 Biomass (i.e. productivity) of litter components (A) and total (B) under different thinning intensities in one year.** CK, T1, T2, T3, T4 represent 0%, 15%, 30%, 45% and 60% thinning intensity, respectively. T: treat, C: component, T:C: interaction. Different letters indicate significant differences between different thinning intensities. ns: non-significant, * p < 0.05, *** p < 0.001.


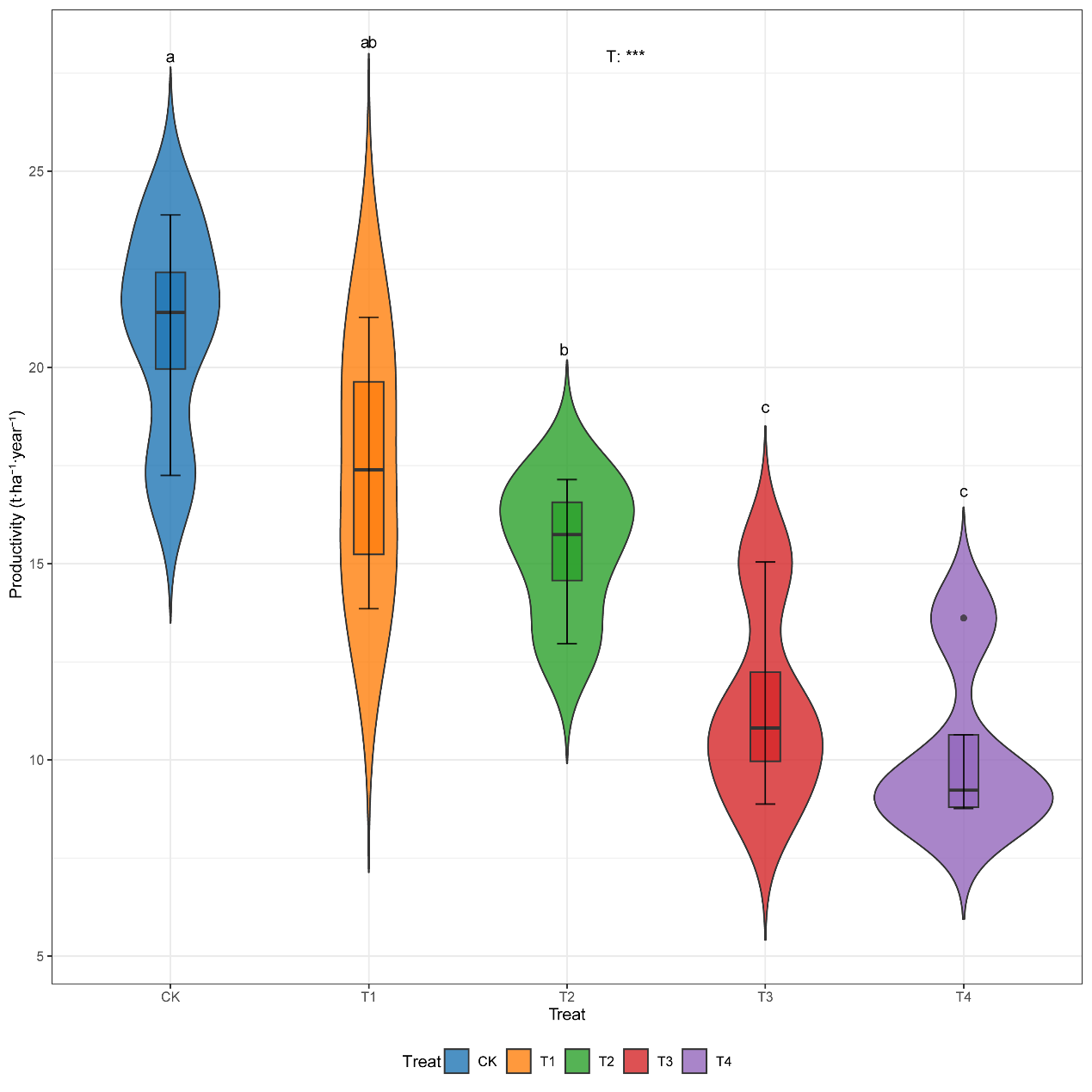


**Figure S9 Productivity of ecosystem under different thinning intensities.** T: treat. CK, T1, T2, T3, T4 represent 0%, 15%, 30%, 45% and 60% thinning intensity, respectively. Different letters indicate significant differences between different thinning intensities. *** p < 0.001.


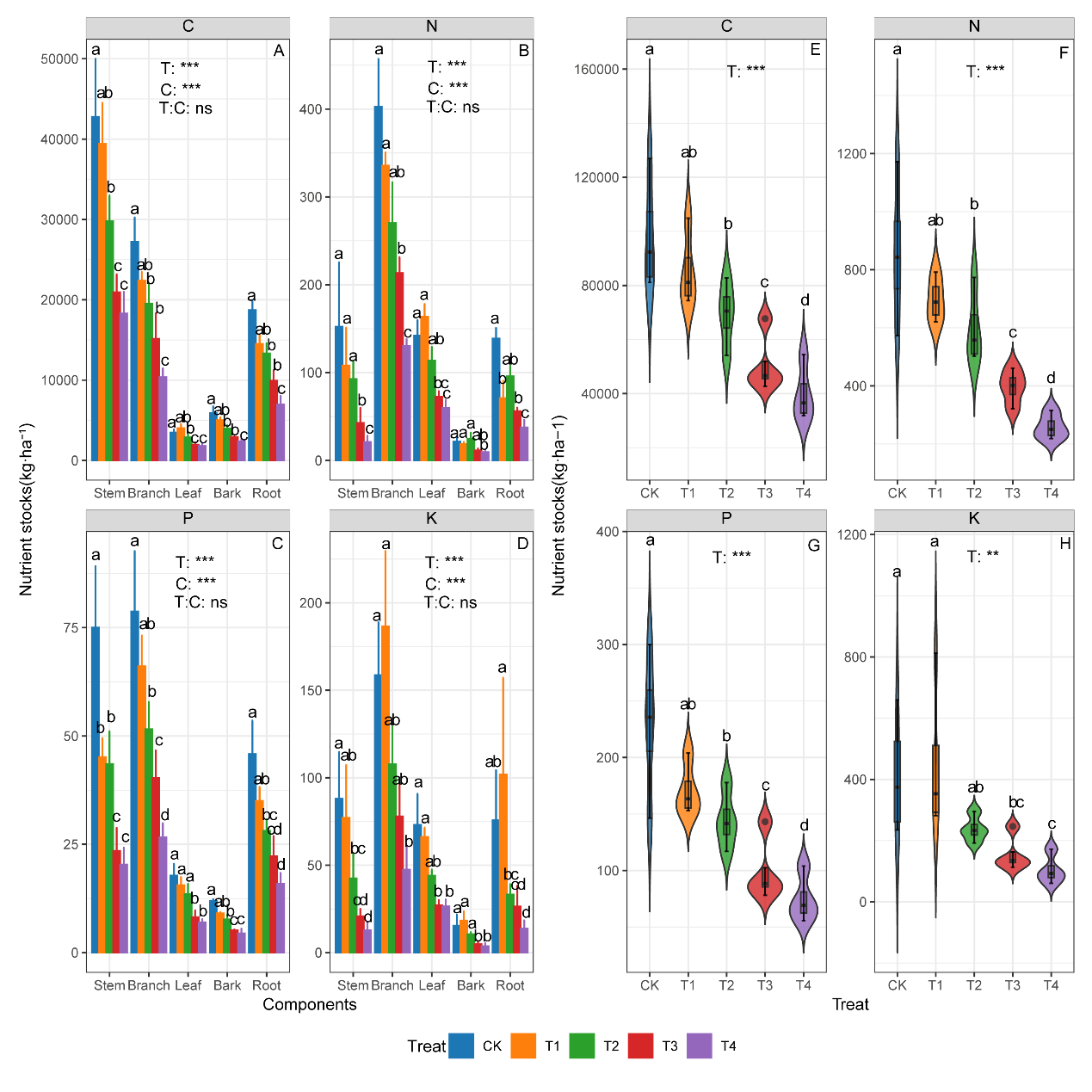


**Figure S10 C, N, P and K stocks of tree organs (A-D) and total (E-H)under different thinning intensities.** CK, T1, T2, T3, T4 represent 0%, 15%, 30%, 45% and 60% thinning intensity, respectively. T: treat, C: component, T:C: interaction. Different letters indicate significant differences between different thinning intensities. ns: non-significant, ** p < 0.01, *** p < 0.001.


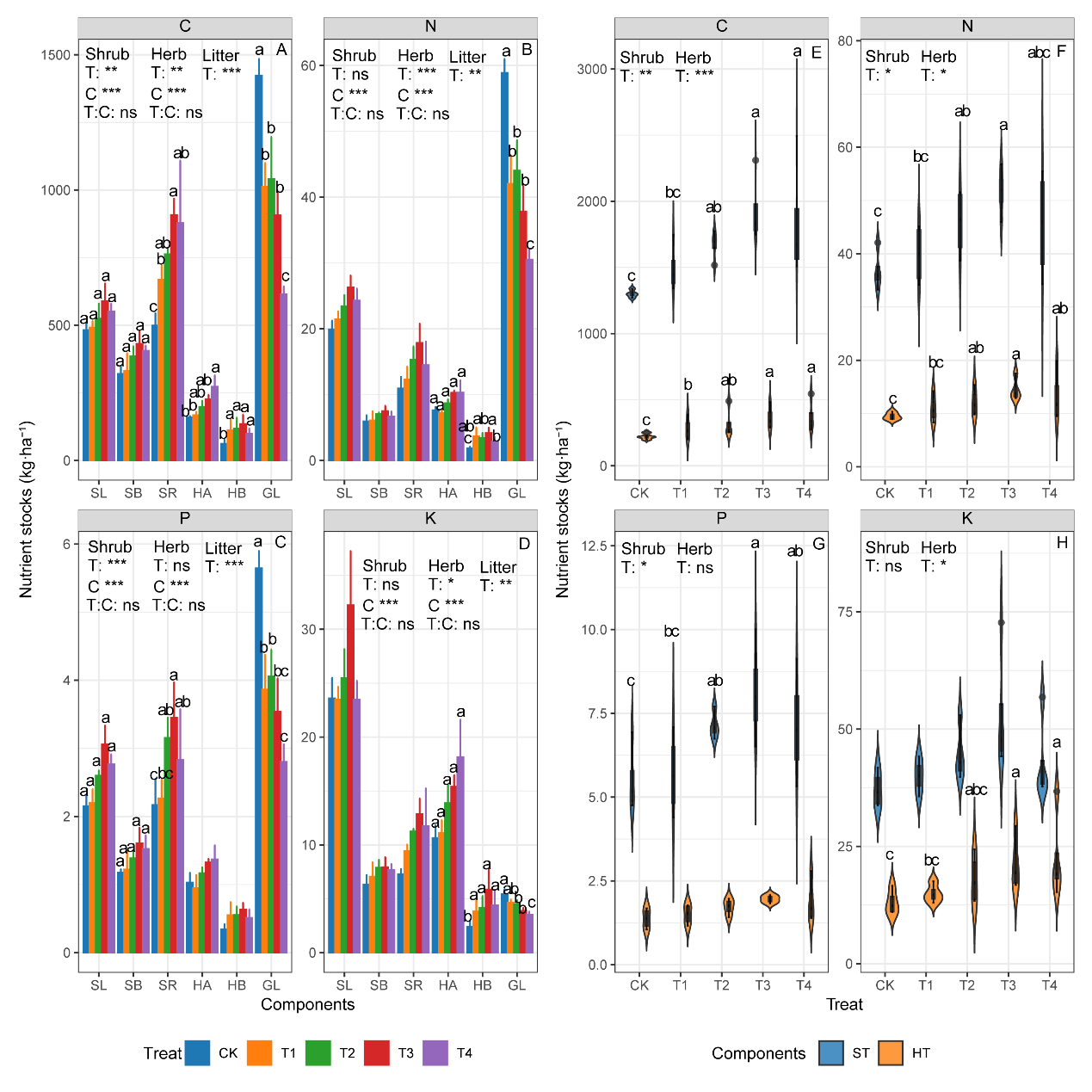


**Figure S11 C, N, P and K stocks of shrub and herb organs and litter (A-D) and total (E-H) under different thinning intensities.** CK, T1, T2, T3, T4 represent 0%, 15%, 30%, 45% and 60% thinning intensity, respectively. T: treat, C: component, T:C: interaction. ST, HT: shrub and herb total attributes, SL, SB, SR: shrub leaf, branch and root, HA, HB: herb aboveground and underground, GL: ground litter. Different letters indicate significant differences between different thinning intensities. ns: non-significant, * p < 0.05, ** p < 0.01, *** p < 0.001.


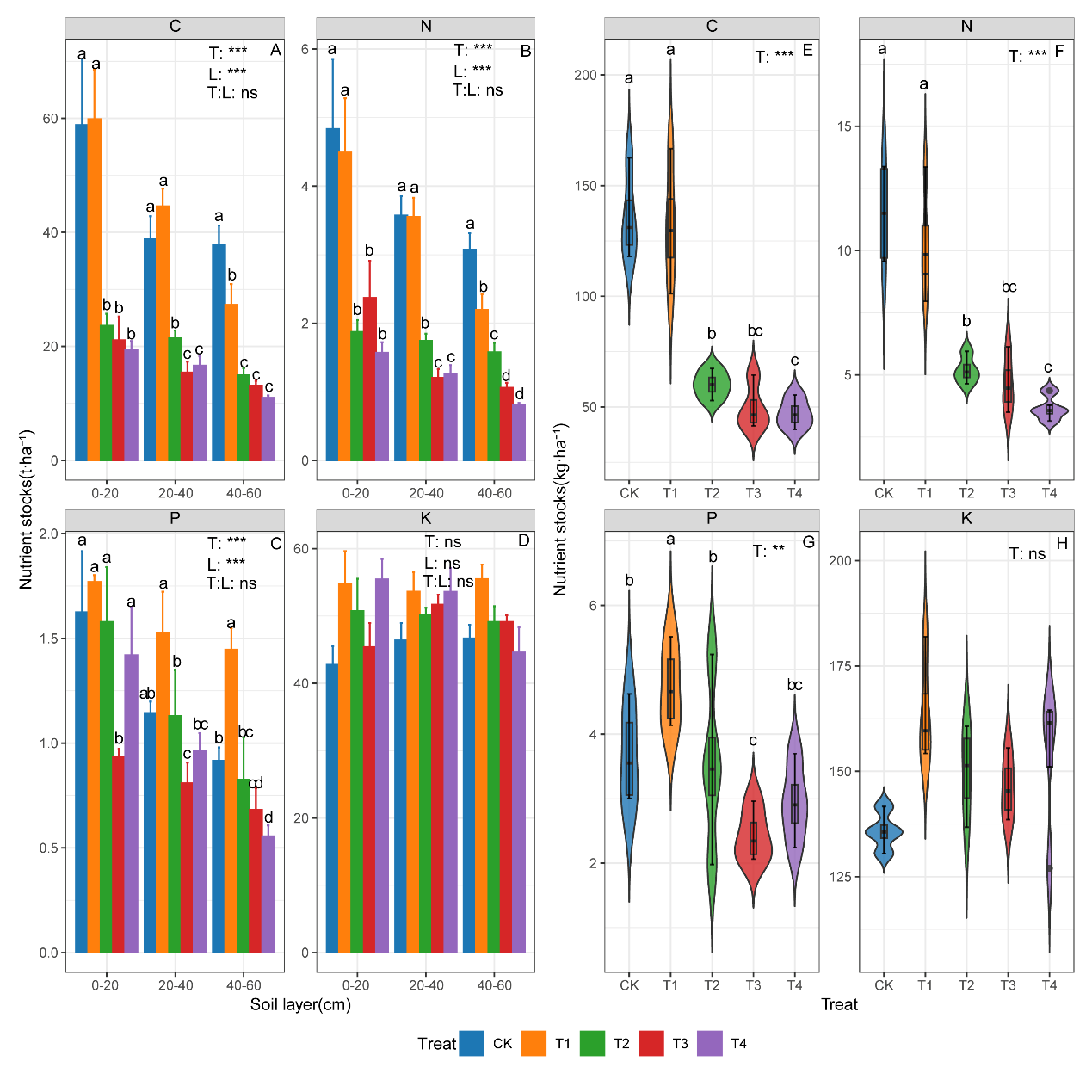


**Figure S12 C, N, P and K stocks of different soil layers (A-D) and total (E-H) under different thinning intensities.** CK, T1, T2, T3, T4 represent 0%, 15%, 30%, 45% and 60% thinning intensity, respectively. T: treat, L: soil layer, T:L: interaction. Different letters indicate significant differences between different thinning intensities. ns: non-significant, ** p < 0.01, *** p < 0.001.


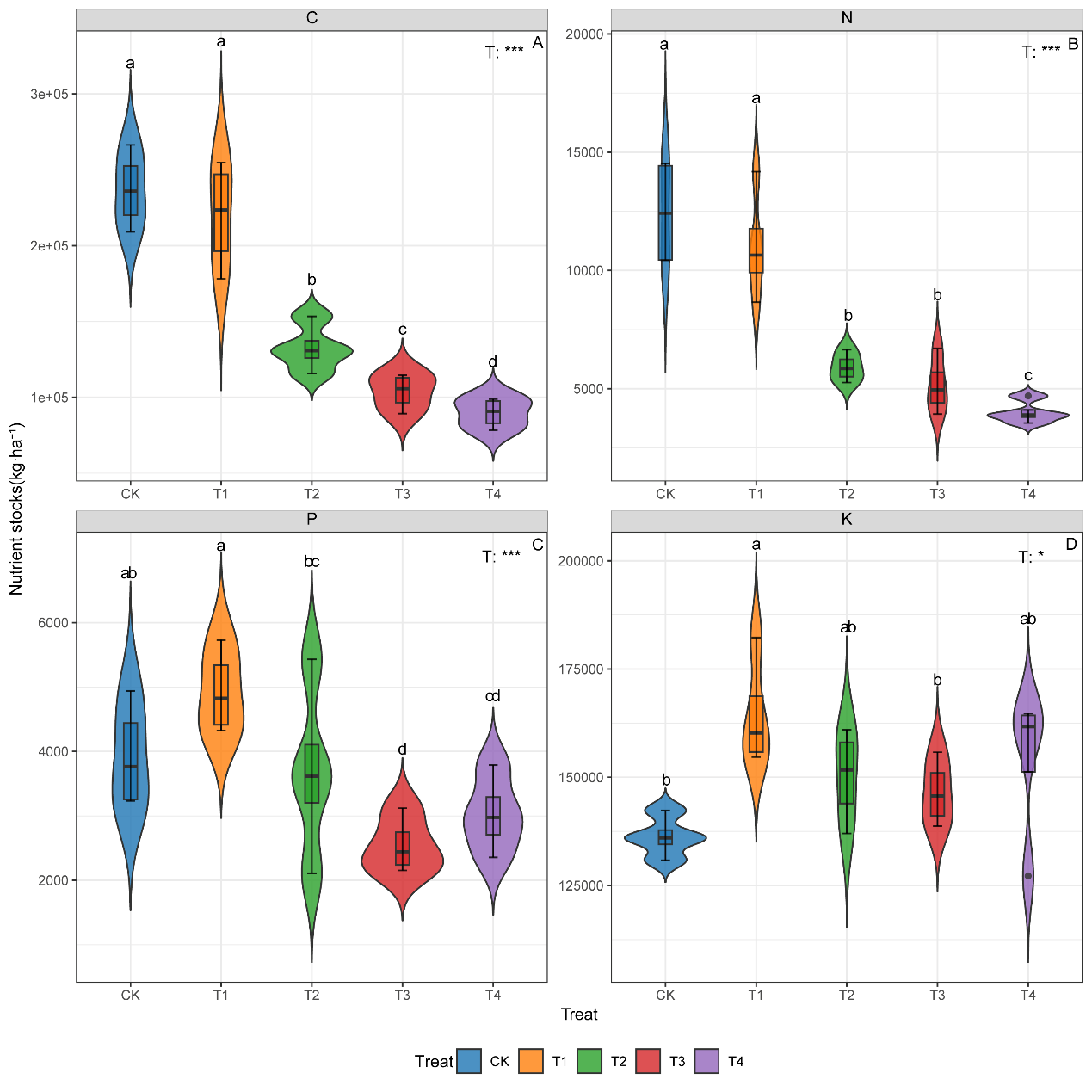


**Figure S13 C, N, P and K stocks of ecosystem under different thinning intensities.** CK, T1, T2, T3, T4 represent 0%, 15%, 30%, 45% and 60% thinning intensity, respectively. T: treat. Different letters indicate significant differences between different thinning intensities. * p < 0.05, *** p < 0.001.


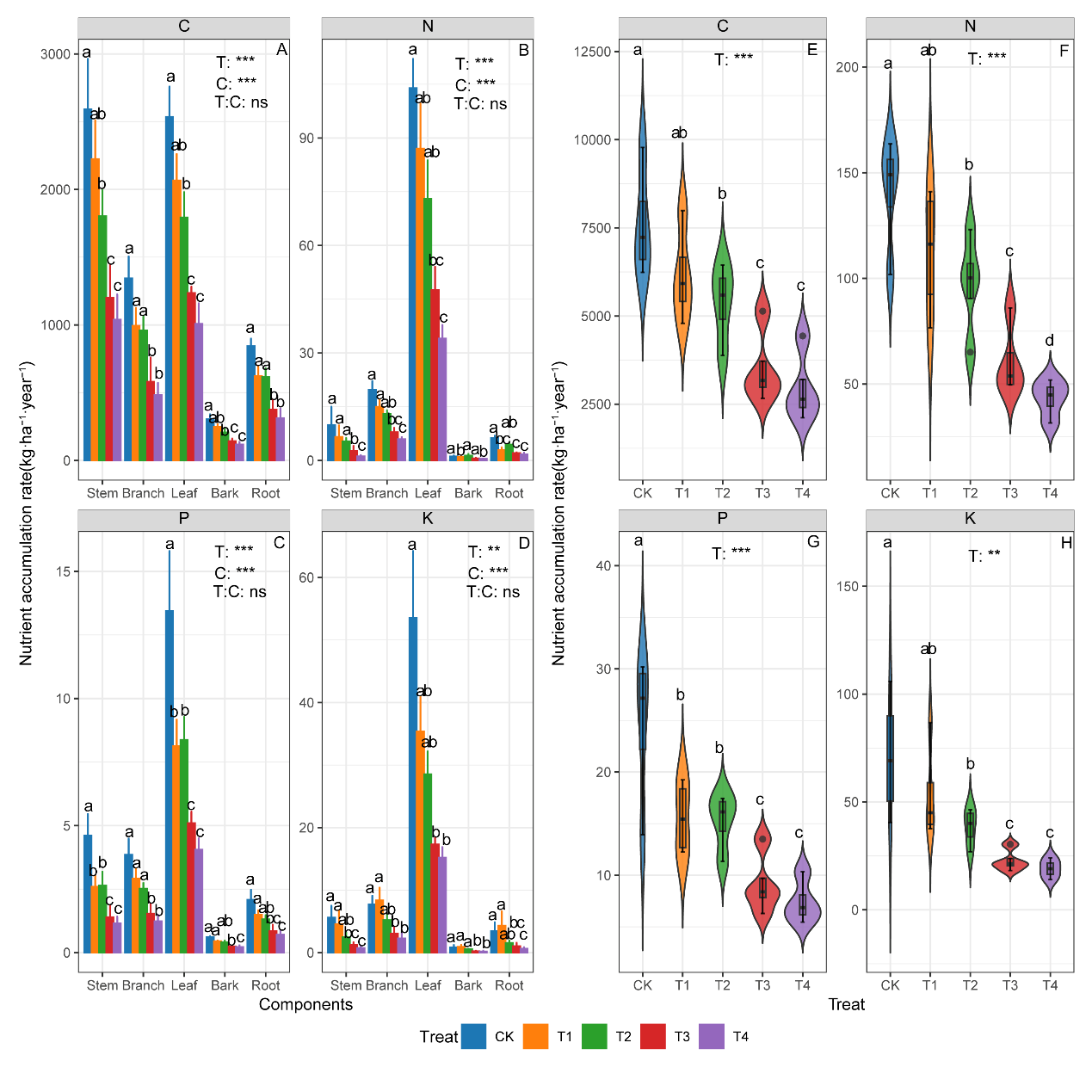


**Figure S14 C, N, P and K accumulation rates of tree organs (A-D) and total (E-H) under different thinning intensities.** CK, T1, T2, T3, T4 represent 0%, 15%, 30%, 45% and 60% thinning intensity, respectively. T: treat, C: component, T:C: interaction. Different letters indicate significant differences between different thinning intensities. ns: non-significant, ** p < 0.01, *** p < 0.001.


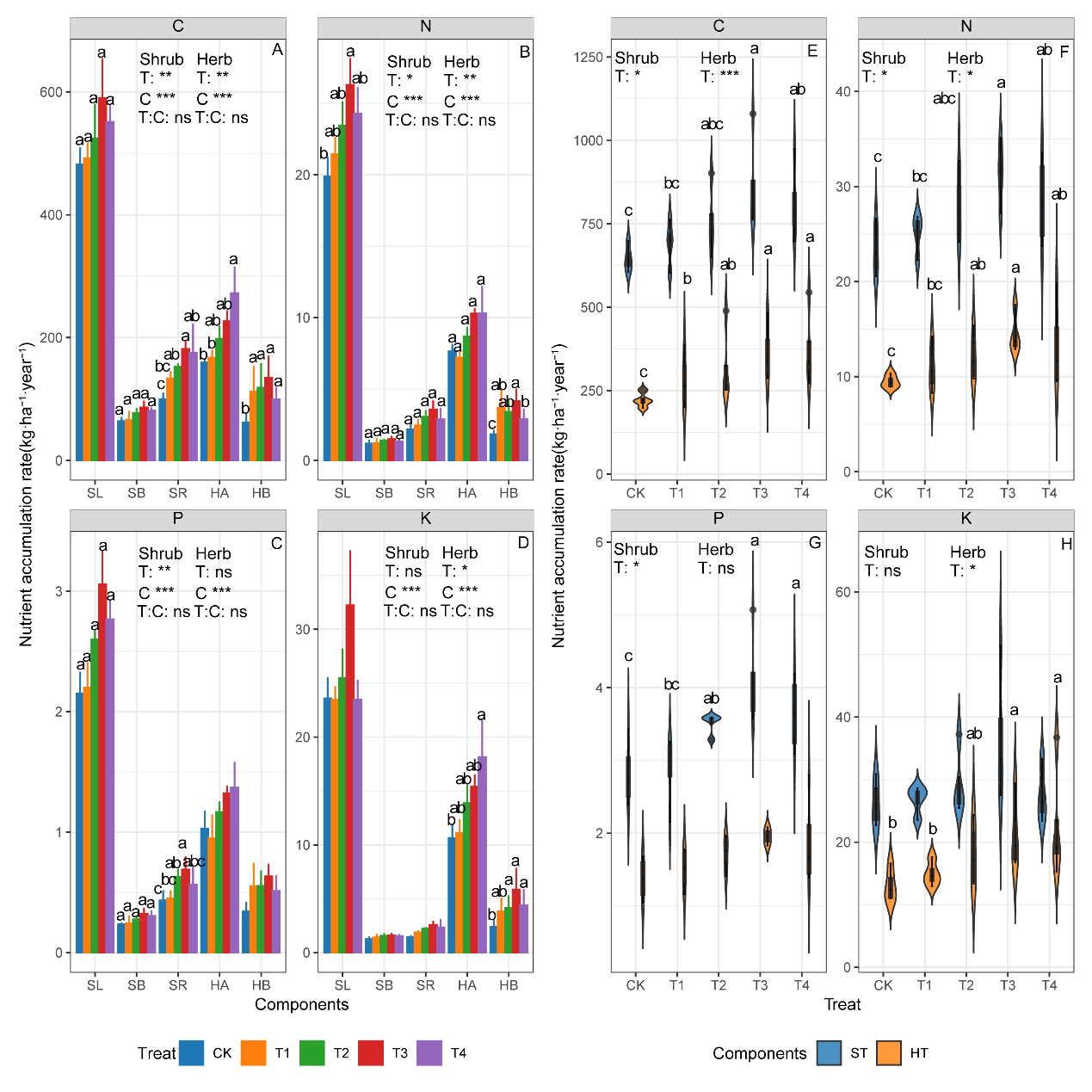


**Figure S15 C, N, P and K accumulation rates of shrub and herb organs (A-D) and total (E-H) under different thinning intensities.** CK, T1, T2, T3, T4 represent 0%, 15%, 30%, 45% and 60% thinning intensity, respectively. T: treat, C: component, T:C: interaction. ST, HT: shrub and herb total attributes, SL, SB, SR: shrub leaf, branch and root, HA, HB: herb aboveground and underground. Different letters indicate significant differences between different thinning intensities. ns: non-significant, * p < 0.05, ** p < 0.01, *** p < 0.001.


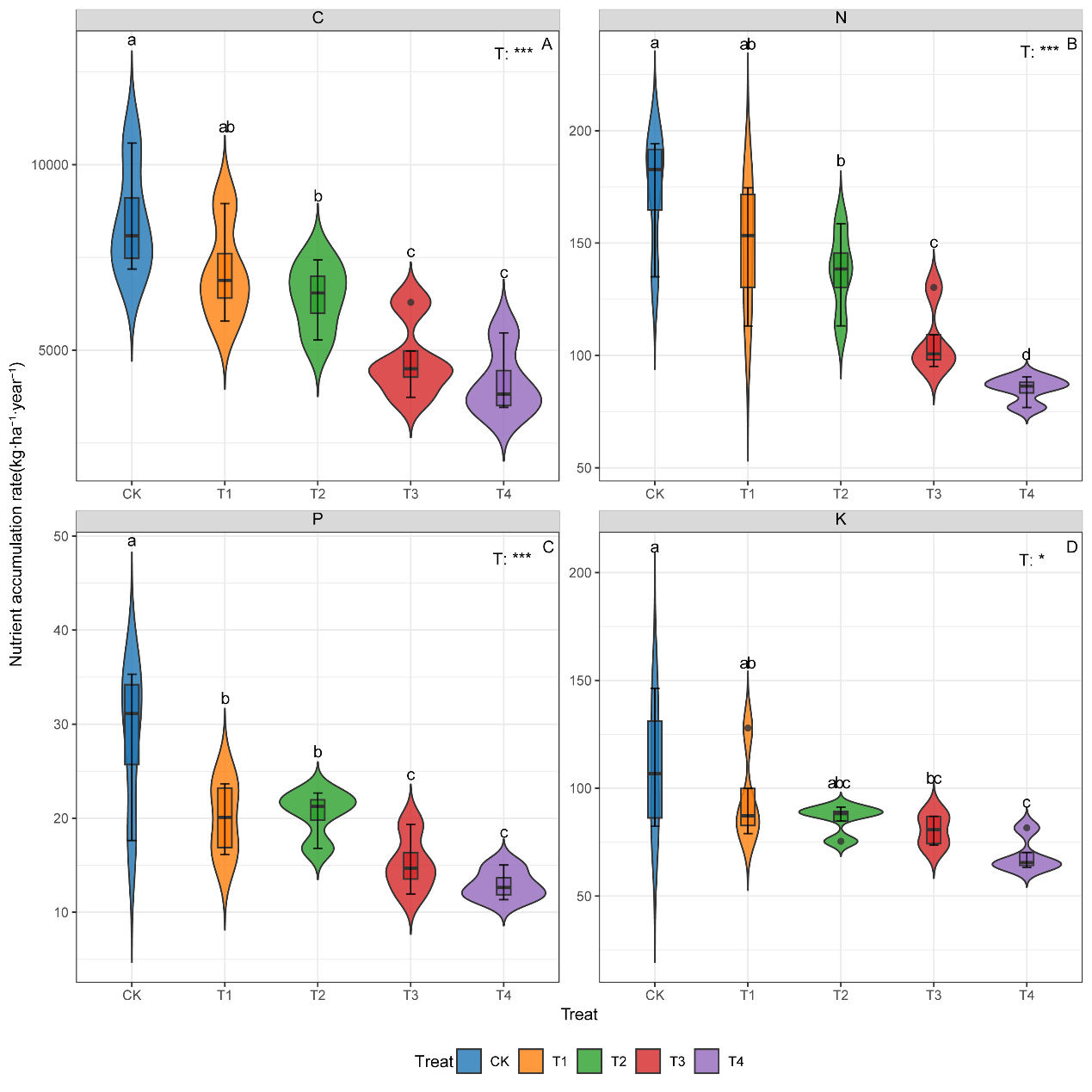


**Figure S16 C, N, P and K accumulation rates of ecosystem under different thinning intensities.** CK, T1, T2, T3, T4 represent 0%, 15%, 30%, 45% and 60% thinning intensity, respectively. T: treat. Different letters indicate significant differences between different thinning intensities. * p < 0.05, *** p < 0.001.


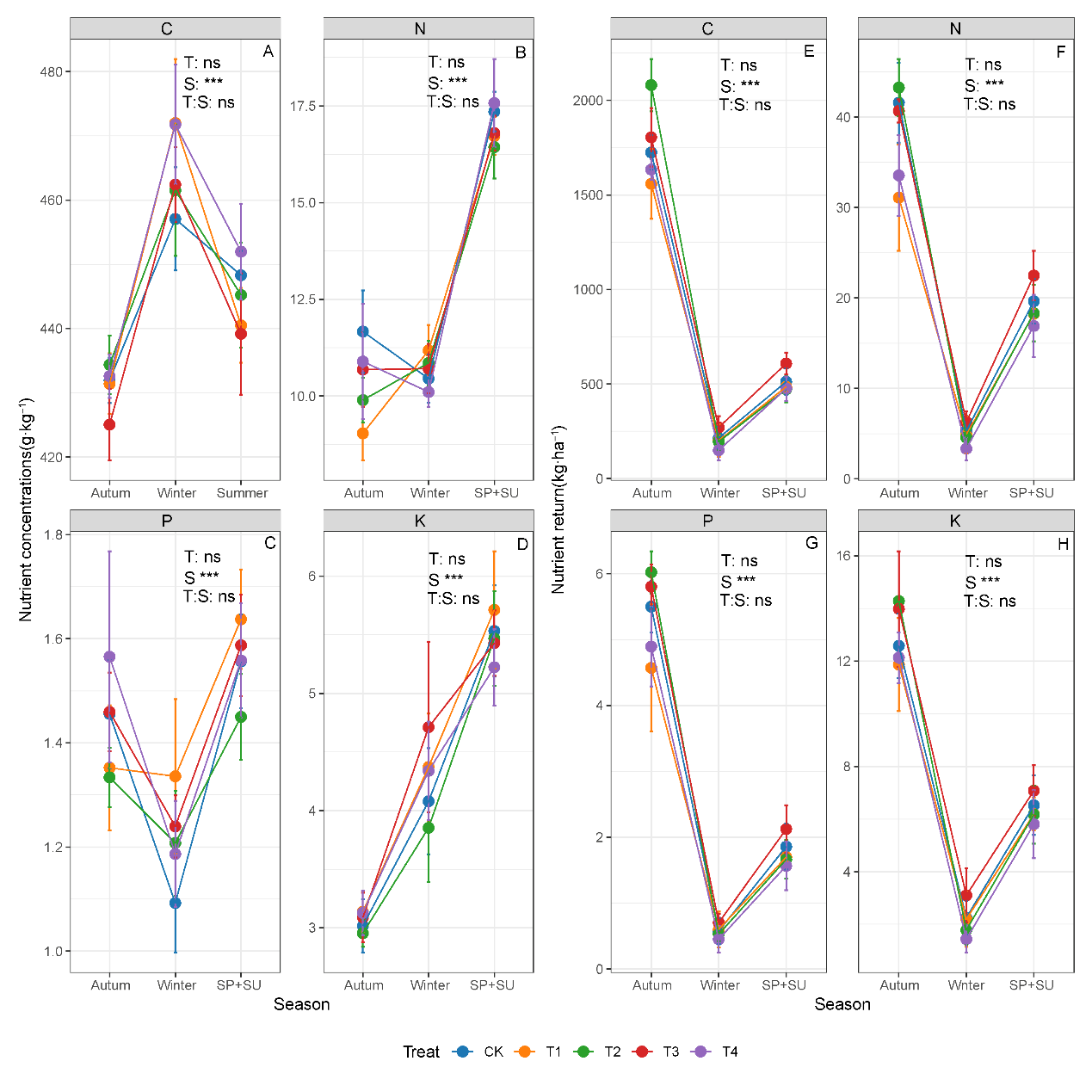


**Figure S17 Seasonal variation of C, N, P and K concentrations and return amounts in tree litter under different thinning intensities.** T: treat, S: season, T:S: interaction, SP+SU: spring and summer (There was too little litter in the two seasons, so they were mixed together). ns: non-significant, *** p < 0.001.


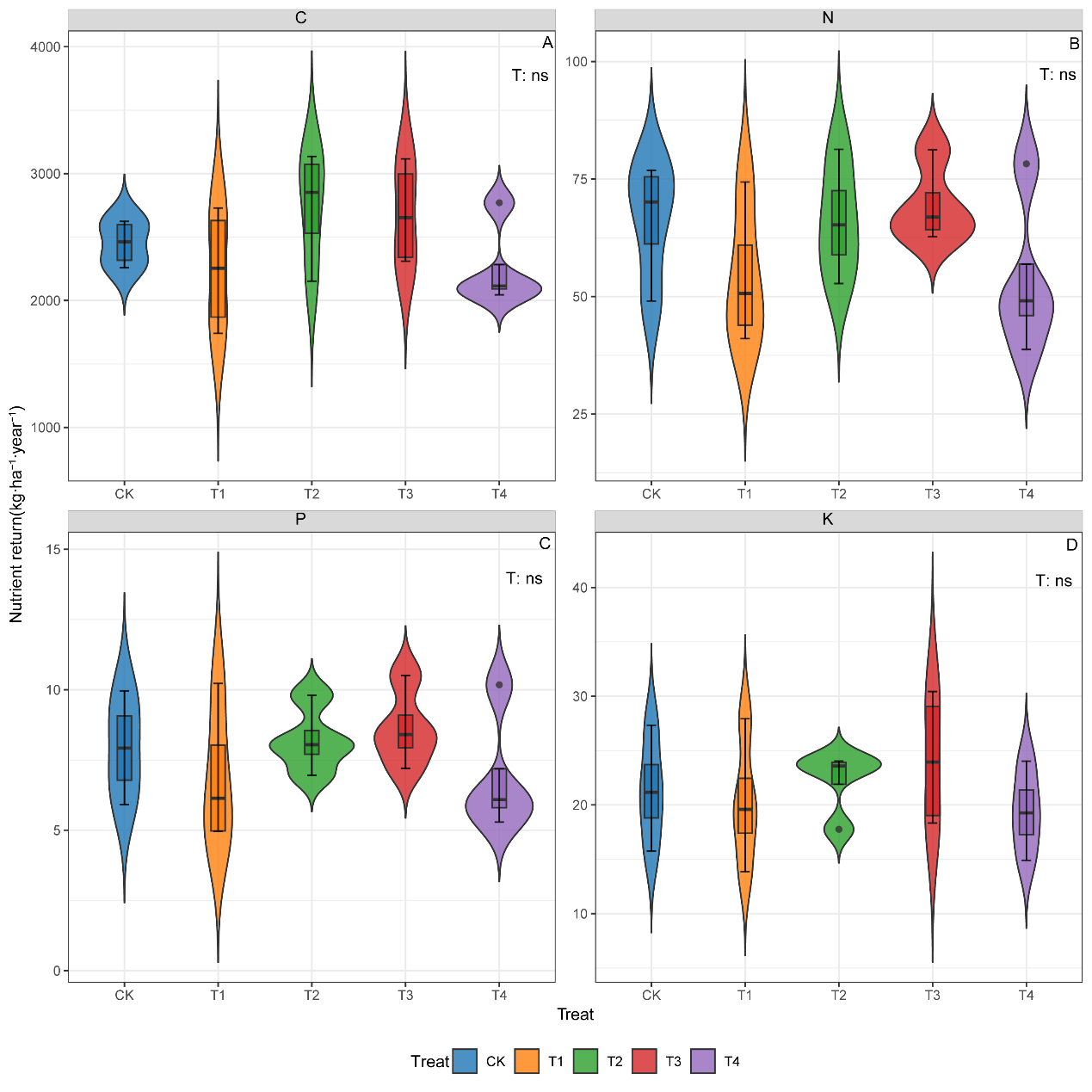


**Figure S18 C, N, P and K return amounts in** **aboveground litter under different thinning intensities.** CK, T1, T2, T3, T4 represent 0%, 15%, 30%, 45% and 60% thinning intensity, respectively. T: treat. Different letters indicate significant differences between different thinning intensities. ns: non-significant.


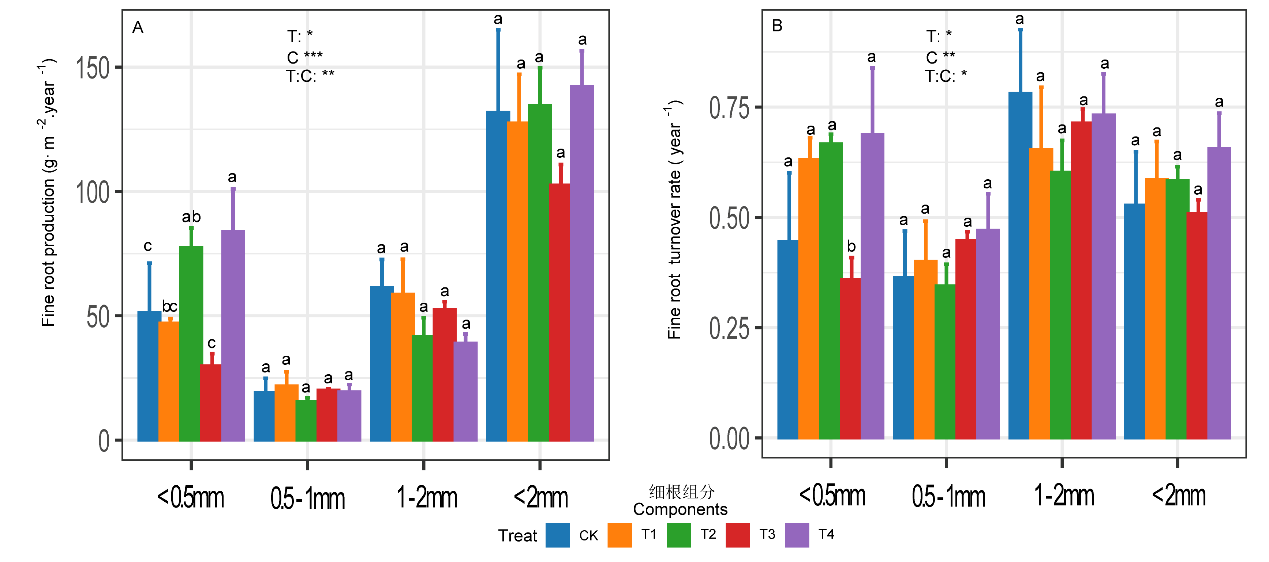


**Figure S19 Fine root productivity (A) and turnover rate (B) of different diameters under different thinning intensities.** CK, T1, T2, T3, T4 represent 0%, 15%, 30%, 45% and 60% thinning intensity, respectively. T: treat, C: fine root component, T:C: interaction. Different letters indicate significant differences between different thinning intensities. * p < 0.05, ** p < 0.01, *** p < 0.001.


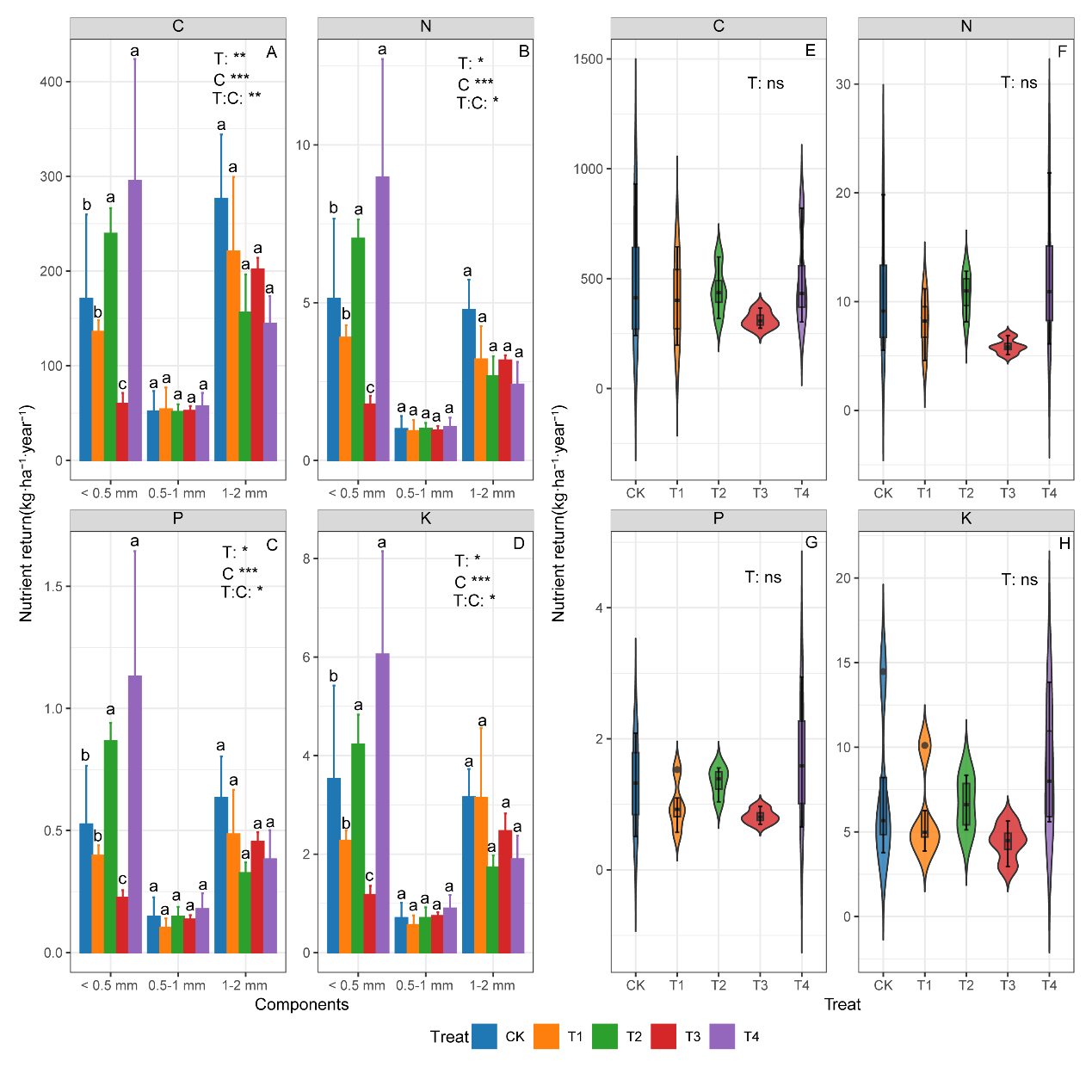


**Figure S20 C, N, P and K return amounts in fine root with different diameters (A-D) and total (E-H) under different thinning intensities.** CK, T1, T2, T3, T4 represent 0%, 15%, 30%, 45% and 60% thinning intensity, respectively. T: treat, C: fine root component, T:C: interaction. Different letters indicate significant differences between different thinning intensities. ns: non-significant, * p < 0.05, ** p < 0.01, *** p < 0.001.


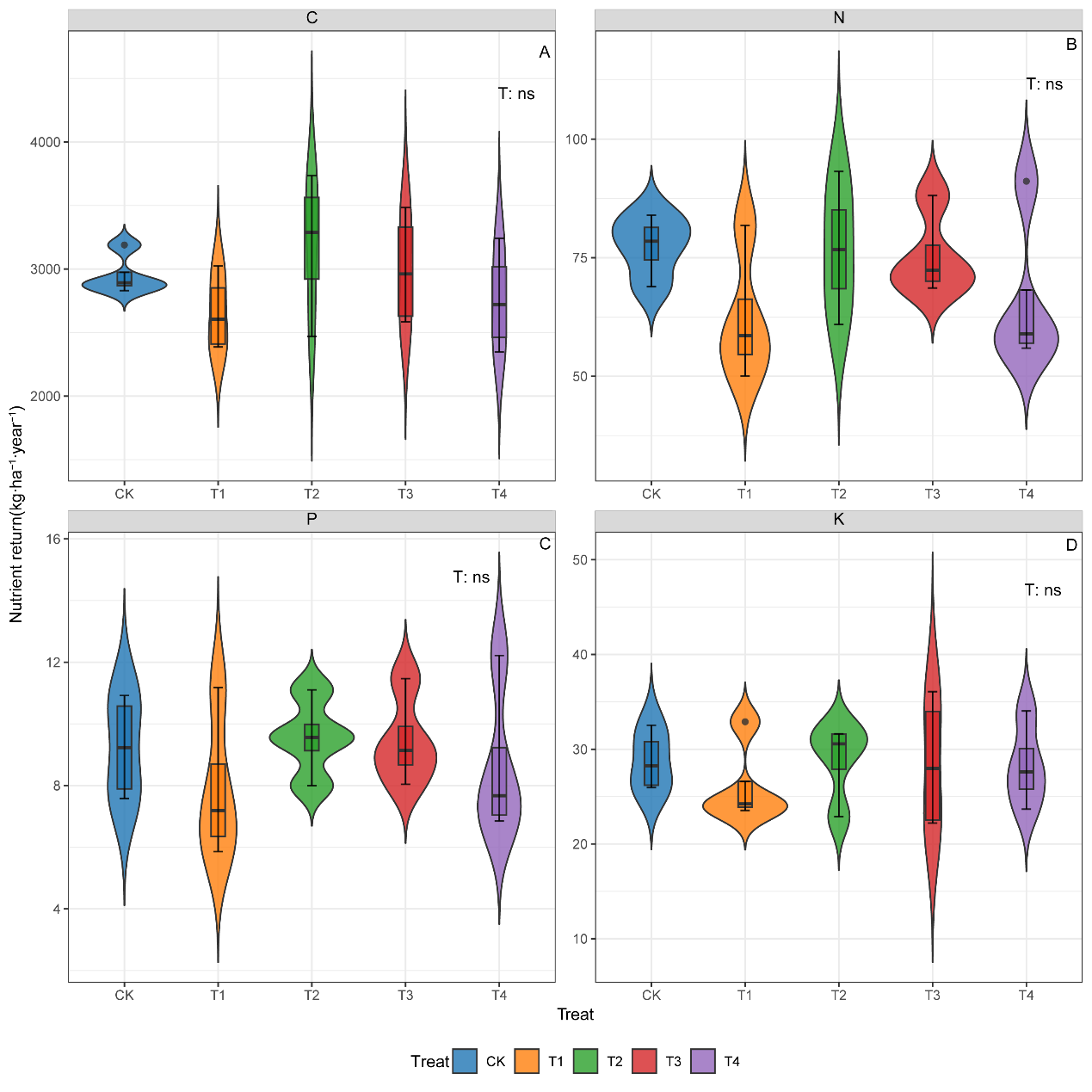


**Figure S21 C, N, P and K return amounts of ecosystem plant under different thinning intensities.** CK, T1, T2, T3, T4 represent 0%, 15%, 30%, 45% and 60% thinning intensity, respectively. T: treat. Different letters indicate significant differences between different thinning intensities. ns: non-significant.

**TABLE S1│****Allometric equations used for biomass calculation.**

| **Species** | **Organ** | **Allometric biomass equation** | **R** | **Source** |
| --- | --- | --- | --- | --- |
| *Quercus aliena var. acutiserrata* | Stem | lnW_S_=0.99253ln(D^2^H)-3.78818 | 0.99763 |  |
| *Quercus aliena var. acutiserrata* | Bark | lnW_BA_=0.75632(D^2^H)-3.9245 | 0.99708 |  |
| *Quercus aliena var. acutiserrata* | Branches | lnW_B_=3.49934lnD-6.50726 | 0.96524 | Chen and Peng, 1996 |
| *Quercus aliena var. acutiserrata* | Leaf | lnW_L_=2.29344InD-4.88581 | 0.97832 |  |
| *Quercus aliena var. acutiserrata* | Root | lnW_R_=2.76435InD-4.20817 | 0.99106 |  |
| *Quercus variabilis* | Stem | lnW_S_=0.9679ln(D^2^H)-3.7447 | 0.99558 |  |
| *Quercus variabilis* | Bark | InW_BA_=0.7156ln(D^2^H)-3.2565 | 0.99037 |  |
| *Quercus variabilis* | Branches | InW_B_=1.0013ln(D^2^H)-4.8449 | 0.99159 | Cheng et al., 2007 |
| *Quercu variabilis* | Leaf | InW_L_=0.6050ln(D^2^H)-3.3569 | 0.98652 |  |
| *Quercus variabilis* | Root | lnW_R_=0.8144ln(D^2^H)-2.9066 | 0.98792 |  |
| *Pinus armandii* | Stem | lnW_S_=1.02363In(D^2^H)-4.49970 | 0.99802 |  |
| *Pinus armandii* | Bark | InW_BA_=0.88417In(D^2^H)-5.38472 | 0.99698 |  |
| *Pinus armandii* | Branches | InW_B_=2.57551InD-4.08452 | 0.98656 | Chen and Peng, 1996 |
| *Pinus armandii* | Leaf | InW_L_=2.75687InD-5.75891 | 0.98004 |  |
| *Pinus armandii* | Root | lnW_R_=0.97120In(D^2^H)-5.26301 | 0.97927 |  |
| *Betula albosinensis* | Stem | lnW_S_=0.91035In(D^2^H)-3.79326 | 0.99721 |  |
| *Betula albosinensis* | Bark | InW_BA_=0.81021In(D^2^H)-4.27750 | 0.99674 |  |
| *Betula albosinensis* | Branches | InW_B_=3.35934lnD-5.93511 | 0.98584 | Chen and Peng, 1996 |
| *Betula albosinensis* | Leaf | InW_L_=2.39007lnD-5.56930 | 0.98709 |  |
| *Betula albosinensis* | Root | InW_R_=2.68879InD-4.33607 | 0.99292 |  |
| *Picea asperata* | Stem | lnW_S_=0.9434ln(D^2^H)-3.9744 | 0.9858 |  |
| *Picea asperata* | Bark | lnW_BA_=0.893ln(D^2^H)-5.5587 | 0.9855 |  |
| *Picea asperata* | Branches | lnW_B_=0.9257ln(D^2^H)-4.635 | 0.955 | Cheng et al., 2007 |
| *Picea asperata* | Leaf | lnW_L_=0.9753ln(D^2^H)-5.9391 | 0.9848 |  |
| *Picea asperata* | Root | lnW_R_=0.9457ln(D^2^H)-5.2791 | 0.9919 |  |
| *Populus davidiana* | Stem | lnW_S_=0.9631ln(D^2^H)-3.8023 | 0.9919 |  |
| *Populus davidiana* | Bark | lnW_BA_=0.9682ln(D^2^H)-5.833 | 0.9561 |  |
| *Populus davidiana* | Branches | lnW_B_=1.0903ln(D^2^H)-5.907 | 0.943 | Cheng et al., 2007 |
| *Populus davidiana* | Leaf | lnW_L_=0.6104ln(D^2^H)-3.9108 | 0.791 |  |
| *Populus davidiana* | Root | lnW_R_=0.7692ln(D^2^H)-3.2756 | 0.9815 |  |
| *Pinus tabuliformis* | Stem | lnWs=1.04086In(D^2^H)-4.63143 | 0.995 |  |
| *Pinus tabuliformis* | Bark | InW_BA_=0.77396In(D^2^H)-4.69348 | 0.991 |  |
| *Pinus tabuliformis* | Branches | InW_B_=2.57733InD-4.08026 | 0.992 | Chen and Peng, 1996 |
| *Pinus tabuliformis* | Leaf | InW_L_=2.57495InD-5.11712 | 0.987 |  |
| *Pinus tabuliformis* | Root | lnW_R_=2.28692InD-4.14198 | 0.987 |  |
| *Larix principis-rupprechtii* | Stem | lnWs=0.99794In(D^2^H)-4.29251 | 0.993 |  |
| *Larix principis-rupprechtii* | Bark | InW_BA_=0.80398In(D^2^H)-4.53535 | 0.988 |  |
| *Larix principis-rupprechtii* | Branches | InW_B_=2.04597InD-2.55078 | 0.977 | Chen and Peng, 1996 |
| *Larix principis-rupprechtii* | Leaf | InW_L_=1.90488InD-3.44704 | 0.974 |  |
| *Larix principis-rupprechtii* | Root | InW_R_=2.18625InD-3.46236 | 0.987 |  |
| Other broad leaf species | Stem | lnW_S_=0.9803ln(D^2^H)-3.8852 | 0.9844 |  |
| Other broad leaf species | Bark | lnW_BA_=0.7713ln(D^2^H)-4.448 | 0.914 |  |
| Other broad leaf species | Branches | lnW_B_=1.0851ln(D^2^H)-5.6360 | 0.8869 | Cheng et al., 2007 |
| Other broad leaf species | Leaf | lnW_L=_0.7377ln(D^2^H)-4.5320 | 0.8705 |  |
| Other broad leaf species | Root | lnW_R_=0.7758ln(D^2^H)-3.1575 | 0.8282 |  |

W: the dry biomass (kg) of a tree component (e.g. stem, bark, branch, leaf and root), D: diameter at breast height for tree (cm), H: height of tree (m), R: Correlation coefficient.

**References:**

Chen, C., Peng, H., 1996. Existing stock and productivity of main forest types in Huoditang forest area of Qinling Mountains. Journal of Northwest Forestry College 11, 92-102, https://doi.org/CNKI:SUN:XBLX.0.1996-S1-012.

Cheng, T., Ma, Q., Feng, Z., Luo, X., 2007. Research on forest biomass in Xiaolong Mountains, Gansu Province. Journal of Beijing Forestry University 29, 31-36, https://doi.org/10.3321/j.issn:1000-1522.2007.01.006.

**TABLE S2│Basic stand characteristics and soil properties.**

| Characteristic | CK | T1 | T2 | T3 | T4 | p |
| --- | --- | --- | --- | --- | --- | --- |
| Thinning intensity | 0% | 15% | 30% | 40% | 60% |  |
| Tree |  |  |  |  |  |  |
| DBH (cm) | 16.77±1.07 | 18.41±1.33 | 17.74±1.13 | 17.06±1.16 | 17.31±0.74 | ns |
| Tree height (m) | 14.03±0.67 | 14.72±0.97 | 13.8±0.61 | 13.15±0.63 | 12.59±0.35 | ns |
| Understory plants |  |  |  |  |  |  |
| Shannon-Wiener–herb | 1.96±0.05b | 2.13±0.08ab | 2.28±0.13ab | 2.22±0.13ab | 2.39±0.15a | * |
| Shannon-Wiener–shrub | 1.84±0.09b | 1.91±0.17b | 2.25±0.03a | 2.01±0.05b | 2.19±0.13ab | * |
| Soil |  |  |  |  |  |  |
| Water content (%) | 35.5±0.43 | 29.49±0.95 | 29.87±2.1 | 33.85±1.94 | 30.11±1.85 | ns |
| Bulk density (g cm^−3^) | 1.28±0.01 | 1.41±0.03 | 1.39±0.05 | 1.32±0.04 | 1.41±0.05 | ns |
| AN (mg kg^−1^) | 20.52±1.52a | 17.66±1.87a | 8.41±0.35b | 7.2±0.25c | 6.42±0.17d | ** |
| AP (mg kg^−1^) | 2.9±0.13a | 2.84±0.28a | 2.07±0.21b | 1.31±0.13c | 1.35±0.14c | *** |
| AK(mg kg^−1^) | 149.77±14.55a | 166.17±15.73a | 110.47±17.76bc | 124.2±16.31b c | 96.29±12.49c | *** |
| pH | 6.01±0.1c | 6.09±0.05c | 6.19±0.09bc | 6.3±0.05ab | 6.45±0.04a | ** |
| Microorganism |  |  |  |  |  |  |
| MBC(mg kg^−1)^ | 684.52±147.6 | 598.65±104.1 | 880.2±173.7 | 486.49±24.64 | 492.07±110.97 | ns |
| MBN(mg kg^−1^) | 171.83±43.44 | 187.62±41.16 | 187.44±21.43 | 165.47±14.44 | 173.32±30.55 | ns |
| MBP(mg kg^−1^) | 50.58±12.43 | 42±8.73 | 37.21±10.96 | 47.77±9.9 | 46.69±11.89 | ns |
| MCN | 4.21±0.8 | 3.29±0.27 | 4.89±1.15 | 3.2±0.19 | 2.9±0.45 | ns |
| MCP | 13.99±2.42 | 16.67±4.85 | 32.11±14.48 | 13.92±1.7 | 13.14±4.26 | ns |
| MNP | 3.94±1.39 | 5.2±1.72 | 6.32±1.56 | 3.81±0.62 | 4.32±1.25 | ns |

CK, T1, T2, T3, T4 represent 0%, 15%, 30%, 45% and 60% thinning intensity, respectively; DBH: diameter at breast height; AN: available nitrogen; AP: available phosphorus; AK: available potassium; MBC: microbial biomass carbon; MBN: microbial biomass nitrogen; MBP: microbial biomass phosphorus; MCN:MBC: MBN ratio; MCP: MBC: MBP ratio; MNP: MBN: MBP ratio. Different lowercase letters indicate significant differences (p < 0.05) between different thinning intensity. ***: p < 0.001, **: p < 0.01, *: p < 0.05, ns: non-significant (p > 0.05).

**TABLE S3│C, N, P, K and stoichiometric characteristics of plant, litter and soil components.**

| Characteristic | CK | T1 | T2 | T3 | T4 | T-p |
| --- | --- | --- | --- | --- | --- | --- |
| Thinning intensity | 0% | 15% | 30% | 40% | 60% |  |
| Tree |  |  |  |  |  |  |
| Leaf |  |  |  |  |  |  |
| C | 404.1±19.3 | 420.89±8.17 | 424.98±10.77 | 433.05±13.25 | 430.85±13.04 | ns |
| N | 16.58±0.79 | 17.44±0.94 | 17.29±2.1 | 16.4±1.25 | 15.12±2.1 | ns |
| P | 2.14±0.38 | 1.64±0.03 | 2±0.13 | 1.78±0.11 | 1.78±0.2 | ns |
| K | 8.51±1.59 | 7.2±0.92 | 6.79±0.67 | 6.06±0.37 | 6.89±1.22 | ns |
| C:N | 24.71±2.31 | 24.41±1.77 | 25.51±2.61 | 26.94±2.45 | 30.43±4.76 | ns |
| C:P | 211.42±44.59 | 256.89±7.65 | 215.32±12.93 | 246.94±20.51 | 250.67±27.48 | ns |
| N:P | 8.32±1.12 | 10.65±0.66 | 8.64±0.74 | 9.42±1.21 | 8.46±0.71 | ns |
| Branch |  |  |  |  |  |  |
| C | 410.84±16.47 | 399.4±30.8 | 426.61±6.19 | 433.06±12.61 | 435.99±7.59 | ns |
| N | 6.07±0.41 | 6±0.48 | 5.88±0.48 | 6.55±0.79 | 5.72±0.79 | ns |
| P | 1.16±0.09 | 1.16±0.05 | 1.13±0.03 | 1.18±0.05 | 1.12±0.05 | ns |
| K | 2.37±0.27 | 3.22±0.56 | 2.3±0.28 | 2.21±0.12 | 1.9±0.32 | ns |
| C:N | 68.99±7.05 | 66.68±1.52 | 73.95±6.06 | 69.94±11.05 | 81.15±11.91 | ns |
| C:P | 363.25±38.37 | 348.49±35.33 | 379.02±16.95 | 367.87±19.12 | 392.52±22.07 | ns |
| N:P | 5.32±0.49 | 5.23±0.52 | 5.21±0.39 | 5.49±0.49 | 5.12±0.7 | ns |
| Stem |  |  |  |  |  |  |
| C | 396.79±29.83 | 424.41±18.01 | 429.94±6.49 | 438.34±5.65 | 432.95±13.75 | ns |
| N | 1.51±0.82 | 1.2±0.48 | 1.37±0.35 | 0.85±0.25 | 0.57±0.22 | ns |
| P | 0.7±0.09 | 0.49±0.04 | 0.64±0.12 | 0.48±0.06 | 0.47±0.02 | ns |
| K | 0.91±0.3 | 0.9±0.34 | 0.64±0.23 | 0.43±0.04 | 0.28±0.06 | ns |
| C:N | 600.23±271.2 | 516.94±143.4 | 385.89±101.51 | 633.99±138.2 | 1404.75±674 | ns |
| C:P | 596.24±85.24 | 886.07±101.5 | 739.63±127.34 | 954.65±99.84 | 918.84±38.14 | ns |
| N:P | 2.25±1.32 | 2.26±0.7 | 2.17±0.47 | 1.67±0.27 | 1.22±0.46 | ns |
| Bark |  |  |  |  |  |  |
| C | 448.6±28.48 | 433.76±17.18 | 445.74±10.39 | 469.67±17.72 | 470.75±24.18 | ns |
| N | 1.64±0.27 | 1.65±0.21 | 2.81±0.69 | 1.83±0.26 | 2±0.51 | ns |
| P | 0.93±0.05 | 0.79±0.03 | 0.86±0.05 | 0.83±0.03 | 0.84±0.06 | ns |
| K | 1.21±0.47 | 1.61±0.43 | 1.21±0.14 | 0.8±0.29 | 0.62±0.22 | ns |
| C:N | 298.81±51.28 | 279.58±41.57 | 196.29±51.03 | 274.8±46.12 | 282.34±62.36 | ns |
| C:P | 493.38±57.8 | 552.19±38.19 | 522.9±29.65 | 569.1±4.7 | 575.42±58.93 | ns |
| N:P | 1.78±0.29 | 2.07±0.24 | 3.22±0.73 | 2.24±0.35 | 2.53±0.75 | ns |
| Root |  |  |  |  |  |  |
| C | 422.76±12.87 | 405.31±18.19 | 438.01±6 | 445.74±15.05 | 428.11±14.34 | ns |
| N | 3.11±0.1 | 2.06±0.6 | 3.14±0.15 | 2.65±0.35 | 2.26±0.2 | ns |
| P | 1.03±0.14 | 0.98±0.05 | 0.93±0.05 | 0.97±0.06 | 0.98±0.06 | ns |
| K | 1.66±0.6 | 2.75±1.41 | 1.08±0.14 | 1.06±0.32 | 0.79±0.2 | ns |
| C:N | 136.5±8.08 | 259.03±83.24 | 140.34±4.94 | 178.94±26.99 | 193.76±19.1 | ns |
| C:P | 432.81±51.62 | 419.31±31.67 | 474.27±25.14 | 463.58±34.15 | 441.56±16.61 | ns |
| N:P | 3.17±0.35 | 2.1±0.61 | 3.4±0.25 | 2.8±0.47 | 2.37±0.3 | ns |
| Organ-p | *** | | | | | |
| Shrub |  |  |  |  |  |  |
| Leaf |  |  |  |  |  |  |
| C | 392.45±4.87a | 373.31±5.6ab | 365.04±6.4b | 363.53±19.8bc | 335.48±6.72c | * |
| N | 16.22±0.84 | 16.31±0.62 | 16.54±1.08 | 16.31±0.47 | 14.88±1.34 | ns |
| P | 1.76±0.15 | 1.66±0.09 | 1.85±0.14 | 1.89±0.08 | 1.69±0.12 | ns |
| K | 19.17±0.89 | 17.85±0.25 | 17.81±0.85 | 19.72±1.95 | 14.29±0.73 | ns |
| C:N | 24.36±1.14 | 23±1.03 | 22.34±1.43 | 22.46±1.93 | 23.15±2.23 | ns |
| C:P | 228.22±22.08 | 226.67±11.87 | 201.35±19.1 | 192.03±3.32 | 201.26±15.89 | ns |
| N:P | 9.34±0.66 | 9.92±0.74 | 9±0.53 | 8.7±0.6 | 8.75±0.21 | ns |
| Branch |  |  |  |  |  |  |
| C | 405.68±22.66 | 387.3±17.07 | 395.15±5.77 | 411.71±7.83 | 391.85±14.48 | ns |
| N | 7.28±0.34 | 6.98±0.48 | 7.49±0.86 | 7.21±0.43 | 6.42±0.48 | ns |
| P | 1.53±0.18 | 1.39±0.1 | 1.44±0.08 | 1.52±0.08 | 1.45±0.11 | ns |
| K | 7.88±0.47 | 8.27±0.28 | 8.11±0.29 | 7.58±0.4 | 7.43±0.5 | ns |
| C:N | 56.35±5.28 | 55.91±3.03 | 54.63±5.56 | 57.65±3.31 | 62.44±6.53 | ns |
| C:P | 270.49±15.34 | 280.54±13 | 276.87±15.78 | 272.61±16.9 | 275.49±26.29 | ns |
| N:P | 4.98±0.66 | 5.03±0.15 | 5.16±0.34 | 4.77±0.37 | 4.43±0.25 | ns |
| Root |  |  |  |  |  |  |
| C | 364.14±14.9 | 382.68±13.38 | 374±9.19 | 397.68±4.67 | 388.71±5.07 | ns |
| N | 7.85±0.41 | 7.08±1.08 | 7.47±0.87 | 7.83±1.15 | 6.87±1.4 | ns |
| P | 1.55±0.13 | 1.3±0.13 | 1.54±0.12 | 1.49±0.17 | 1.26±0.14 | ns |
| K | 5.34±0.23 | 5.43±0.25 | 5.53±0.18 | 5.6±0.43 | 5.09±0.31 | ns |
| C:N | 46.89±3.58 | 58.62±10.19 | 52.46±7.05 | 53.69±6.7 | 64.84±14.33 | ns |
| C:P | 239.86±24.15 | 304.18±32.11 | 246.16±17.09 | 278.65±35.08 | 318.05±30.82 | ns |
| N:P | 5.09±0.19 | 5.38±0.4 | 4.83±0.39 | 5.38±0.75 | 5.45±0.96 | ns |
| Organ-p | *** | | | | | |
| Herb |  |  |  |  |  |  |
| Aboveground |  |  |  |  |  |  |
| C | 367.88±4.58 | 364.46±10.19 | 366.1±11.69 | 359.38±4.01 | 365.25±2.61 | ns |
| N | 17.61±0.99 | 15.75±0.48 | 16.25±0.86 | 16.51±1.05 | 13.75±1.12 | ns |
| P | 2.36±0.29 | 2.09±0.42 | 2.24±0.29 | 2.13±0.18 | 1.85±0.13 | ns |
| K | 24.47±2.46 | 24.24±2.15 | 25.85±2.77 | 24.59±1.67 | 23.97±0.7 | ns |
| C:N | 21.12±1.33 | 23.16±0.47 | 22.81±1.75 | 22.09±1.72 | 27.22±2.66 | ns |
| C:P | 163.32±21.11 | 192.96±31.89 | 175.45±30.25 | 172.42±15.23 | 200.16±13.5 | ns |
| N:P | 7.74±0.86 | 8.29±1.26 | 7.61±0.97 | 7.84±0.58 | 7.66±1.13 | ns |
| Belowground |  |  |  |  |  |  |
| C | 341.93±3.37 | 331.29±15.01 | 349.62±14.56 | 343.03±10.8 | 363.01±15.02 | ns |
| N | 10.9±1.22 | 11.25±0.33 | 10.76±1.26 | 11.01±0.65 | 10.28±0.65 | ns |
| P | 1.9±0.2 | 1.7±0.08 | 1.75±0.14 | 1.73±0.22 | 1.82±0.14 | ns |
| K | 13.3±1.64 | 12.07±0.54 | 12.93±1.19 | 14.45±2.78 | 15±2.03 | ns |
| C:N | 32.52±3.46 | 29.51±1.52 | 34.01±4.64 | 31.54±2.35 | 35.73±2.54 | ns |
| C:P | 185.61±16.94 | 197.67±16.72 | 204.63±23.58 | 209.71±30.47 | 203.76±19.46 | ns |
| N:P | 5.97±1 | 6.67±0.3 | 6.09±0.25 | 6.55±0.51 | 5.69±0.29 | ns |
| Organ-p | *** | | | | | |
| Fine root |  |  |  |  |  |  |
| <0.5mm |  |  |  |  |  |  |
| C | 454.37±2.28 | 445.85±7.99 | 447.05±11.56 | 441.7±7.55 | 441.75±4.21 | ns |
| N | 14.03±0.54 | 12.83±0.86 | 13.23±0.44 | 13.48±0.83 | 13.71±1.18 | ns |
| P | 1.51±0.17 | 1.32±0.14 | 1.63±0.1 | 1.78±0.27 | 1.67±0.22 | ns |
| K | 9.12±1.36 | 7.63±0.9 | 7.84±0.53 | 8.94±0.85 | 9.92±0.68 | ns |
| C:N | 32.52±1.27 | 35.29±2.72 | 33.93±1.53 | 33.26±2.72 | 32.99±2.93 | ns |
| C:P | 310.99±30.61 | 349.44±39.57 | 276.41±18.02 | 268.28±46.05 | 282.29±43.94 | ns |
| N:P | 9.55±0.88 | 9.83±0.4 | 8.17±0.48 | 7.9±0.71 | 8.43±0.58 | ns |
| 0.5-1mm |  |  |  |  |  |  |
| C | 458.62±2.35 | 468.18±5.68 | 463.21±2.44 | 465.82±5.73 | 463.23±7.5 | ns |
| N | 8.91±0.45 | 8.56±0.46 | 8.98±0.5 | 8.39±0.28 | 8.23±0.41 | ns |
| P | 1.24±0.24 | 0.99±0.1 | 1.26±0.15 | 1.21±0.06 | 1.32±0.2 | ns |
| K | 6.5±0.68 | 5.83±1.13 | 6.02±0.92 | 6.65±0.34 | 6.95±0.55 | ns |
| C:N | 51.87±2.43 | 55.07±2.49 | 52.12±3.37 | 55.71±2.1 | 56.8±3.35 | ns |
| C:P | 416.46±78.78 | 484.32±40.89 | 382.66±44.81 | 388.61±20.46 | 374.84±51.65 | ns |
| N:P | 7.89±1.18 | 8.76±0.46 | 7.39±0.95 | 6.99±0.38 | 6.59±0.8 | ns |
| 1-2mm |  |  |  |  |  |  |
| C | 458.46±10.05 | 465.09±6.79 | 457.64±6.59 | 471.02±11.17 | 462.12±2.46 | ns |
| N | 8.23±0.59 | 7.01±0.26 | 8.05±0.43 | 7.43±0.19 | 7.23±0.8 | ns |
| P | 1.05±0.14 | 1.07±0.14 | 1.07±0.16 | 1.06±0.05 | 1.15±0.2 | ns |
| K | 5.55±0.44 | 6.63±1 | 5.7±0.91 | 5.75±0.78 | 5.98±0.7 | ns |
| C:N | 56.9±5.71 | 66.59±2.28 | 57.54±4.18 | 63.52±1.79 | 66.41±7.6 | ns |
| C:P | 461.87±67.76 | 464.07±73.54 | 455.5±67.32 | 447.85±24.29 | 436.26±70.72 | ns |
| N:P | 8.08±0.82 | 6.95±1.02 | 7.9±1.03 | 7.06±0.37 | 6.53±0.66 | ns |
| Diameter-p | *** | | | | | |
| Litter |  |  |  |  |  |  |
| Tree litter |  |  |  |  |  |  |
| C | 430.43±5.19 | 427.9±6.56 | 436.24±6.96 | 421.95±6.97 | 428.84±2.74 | ns |
| N | 10.36±0.85 | 8.45±0.92 | 9.08±0.43 | 9.7±0.79 | 8.71±0.88 | ns |
| P | 1.39±0.15 | 1.23±0.14 | 1.27±0.06 | 1.37±0.06 | 1.27±0.11 | ns |
| K | 3.13±0.23 | 3.25±0.24 | 3.03±0.17 | 3.21±0.27 | 3.19±0.28 | ns |
| C:N | 42.48±3.88 | 52.51±5.89 | 48.35±2.39 | 44.56±4.3 | 51.17±6.44 | ns |
| C:P | 320.28±34.39 | 360.58±35.69 | 346.45±20.78 | 311.05±18.69 | 345.18±31.45 | ns |
| N:P | 7.61±0.81 | 6.91±0.34 | 7.17±0.23 | 7.06±0.35 | 6.83±0.33 | ns |
| Ground litter |  |  |  |  |  |  |
| C | 395.8±8.43 | 381.59±4.07 | 369.4±20.54 | 400.62±17.28 | 328.93±18.56 | ns |
| N | 16.48±1 | 15.78±0.55 | 15.9±0.28 | 16.68±0.72 | 16.41±1.35 | ns |
| P | 1.58±0.12 | 1.45±0.11 | 1.47±0.07 | 1.53±0.05 | 1.48±0.07 | ns |
| K | 1.52±0.13 | 1.77±0.12 | 1.65±0.14 | 1.74±0.12 | 1.87±0.06 | ns |
| C:N | 24.26±1.41 | 24.27±0.92 | 23.31±1.64 | 24.07±0.88 | 20.28±1.27 | ns |
| C:P | 253.72±16.64 | 268.09±18.52 | 252.21±17.52 | 262.91±17.28 | 224±17.47 | ns |
| N:P | 10.45±0.18 | 11.06±0.7 | 10.87±0.59 | 10.92±0.59 | 11.09±0.77 | ns |
| Soil |  |  |  |  |  |  |
| 0-20cm |  |  |  |  |  |  |
| C | 26.64±5.08a | 23.83±3.18a | 9.22±0.47b | 9.54±1.59b | 7.37±0.55b | *** |
| N | 2.18±0.45a | 1.79±0.3a | 0.73±0.04bbc | 1.07±0.23b | 0.6±0.05c | *** |
| P | 0.74±0.13a | 0.71±0.04ab | 0.64±0.13abc | 0.43±0.02c | 0.54±0.08bc | * |
| K | 19.44±0.83 | 21.74±1.3 | 19.66±0.54 | 20.64±0.78 | 21.16±0.94 | ns |
| C:N | 12.35±0.42 | 13.51±0.54 | 12.61±0.38 | 10.28±2.32 | 12.59±1.5 | ns |
| C:P | 35.92±1.88a | 33.91±4.95a | 16.56±3.4cd | 22.64±4.43bc | 14.15±1.12d | *** |
| N:P | 2.93±0.23a | 2.54±0.45ab | 1.3±0.24bc | 2.55±0.59a | 1.18±0.2c | ** |
| 20-40cm |  |  |  |  |  |  |
| C | 14.47±1.05a | 15.82±1.05a | 7.65±0.47b | 5.53±0.67c | 5.76±0.45c | *** |
| N | 1.35±0.14a | 1.26±0.1a | 0.62±0.04b | 0.43±0.05c | 0.44±0.03c | *** |
| P | 0.43±0.03ab | 0.54±0.06a | 0.41±0.09bc | 0.29±0.03d | 0.33±0.02c | *** |
| K | 17.34±0.42 | 18.97±0.46 | 17.9±0.61 | 18.52±0.34 | 18.57±0.88 | ns |
| C:N | 11.2±1.54 | 12.59±0.17 | 12.3±0.1 | 12.77±0.51 | 13.13±0.33 | ns |
| C:P | 34.21±3.82a | 30.13±3.09a | 21.36±4.1b | 20.02±3.69b | 17.42±0.97b | ** |
| N:P | 3.14±0.27a | 2.4±0.26ab | 1.73±0.33bc | 1.56±0.25c | 1.33±0.08c | ** |
| 40-60cm |  |  |  |  |  |  |
| C | 13.34±0.78a | 8.75±1.15b | 5.08±0.52c | 4.48±0.37c | 3.77±0.2c | *** |
| N | 1.08±0.05a | 0.7±0.07b | 0.53±0.03c | 0.36±0.03d | 0.28±0.01e | *** |
| P | 0.32±0.02b | 0.47±0.04a | 0.27±0.06bc | 0.23±0.03c | 0.19±0.02c | *** |
| K | 16.54±0.7 | 17.78±0.48 | 16.52±0.47 | 16.69±0.49 | 15.1±0.65 | ns |
| C:N | 12.3±0.23 | 12.29±0.51 | 9.77±1.31 | 12.38±0.23 | 13.53±0.81 | ns |
| C:P | 41.83±3.82a | 18.99±2.58b | 22.87±6.8b | 20.07±2.08b | 20.54±2.67b | * |
| N:P | 3.39±0.26a | 1.53±0.18bc | 2.21±0.44b | 1.61±0.14bc | 1.5±0.1c | *** |
| Layer-p | *** | | | | | |

C, N, P and K: litter carbon, nitrogen, phosphorus and potassium content; CK, T1, T2, T3, T4 represent 0%, 15%, 30%, 45% and 60% thinning intensity, respectively; ***: p < 0.001, **: p < 0.01, *: p < 0.05, ns: non-significant (p > 0.05).

**TABLE S4│C, N, P, K and stoichiometric characteristics at the ecosystem level of tree, shrub and herb functional groups.**

| Characteristic | CK | T1 | T2 | T3 | T4 | p |
| --- | --- | --- | --- | --- | --- | --- |
| Thinning intensity | 0% | 15% | 30% | 40% | 60% |  |
| Tree |  |  |  |  |  |  |
| C | 409.21±18.7 | 413.69±21.03 | 431.65±4.08 | 440.74±6.09 | 434.97±10.5 | ns |
| N | 3.61±0.51 | 3.41±0.23 | 3.73±0.3 | 3.48±0.23 | 2.94±0.42 | ns |
| P | 0.95±0.1 | 0.83±0.05 | 0.9±0.05 | 0.85±0.03 | 0.81±0.02 | ns |
| K | 1.74±0.39 | 2.19±0.56 | 1.49±0.1 | 1.33±0.1 | 1.1±0.1 | ns |
| C:N | 121.59±19.45 | 123.91±13.35 | 118±10.04 | 128.04±7.55 | 156.68±21.01 | ns |
| C:P | 444.51±53.25 | 506.04±50.52 | 483.94±32.02 | 518.93±16.02 | 538.35±13.71 | ns |
| N:P | 3.83±0.5 | 4.1±0.19 | 4.13±0.14 | 4.11±0.32 | 3.64±0.5 | ns |
| Shrub |  |  |  |  |  |  |
| C | 381.81±5.32 | 380.54±1.25 | 376.23±2.54 | 389.52±9.5 | 368.94±5.02 | ns |
| N | 10.78±0.47 | 10.16±0.4 | 10.35±0.88 | 10.53±0.69 | 9.33±1.1 | ns |
| P | 1.61±0.13 | 1.44±0.09 | 1.62±0.1 | 1.64±0.1 | 1.45±0.12 | ns |
| K | 10.93±0.67 | 10.23±0.23 | 10.02±0.35 | 10.63±0.84 | 8.68±0.19 | ns |
| C:N | 35.6±1.5 | 37.63±1.34 | 37.12±3.1 | 37.62±3.24 | 41.25±4.8 | ns |
| C:P | 241.25±17.45 | 267.23±16.11 | 235.13±12.79 | 240.01±11.83 | 260.68±21.86 | ns |
| N:P | 6.75±0.26 | 7.1±0.29 | 6.4±0.33 | 6.52±0.64 | 6.43±0.44 | ns |
| Herb |  |  |  |  |  |  |
| C | 360.56±4.43 | 354.6±6.39 | 361.52±11.74 | 354.22±5.19 | 364.68±3.73 | ns |
| N | 15.57±1.13 | 14.09±0.68 | 14.25±1.05 | 14.44±0.86 | 12.86±0.75 | ns |
| P | 2.24±0.26 | 2.01±0.32 | 2.07±0.23 | 1.99±0.2 | 1.85±0.12 | ns |
| K | 21.4±2.33 | 19.89±2.16 | 21.04±2.02 | 20.93±1.72 | 21.62±0.84 | ns |
| C:N | 23.53±1.76 | 25.28±0.87 | 25.94±2.61 | 24.85±1.79 | 28.66±1.81 | ns |
| C:P | 167.64±19.98 | 187.74±23.64 | 183.82±28.62 | 184.67±21.55 | 200.22±14.26 | ns |
| N:P | 7.16±0.79 | 7.36±0.72 | 7.01±0.5 | 7.38±0.45 | 7.14±0.88 | ns |

C, N, P and K: litter carbon, nitrogen, phosphorus and potassium content; CK, T1, T2, T3, T4 represent 0%, 15%, 30%, 45% and 60% thinning intensity, respectively; ***: p < 0.001, **: p < 0.01, *: p < 0.05, ns: non-significant (p > 0.05).

**SI Materials and Methods**

**Data calculation**

**(1) Biomass calculation**

Tree layer biomass: use the allometric growth equation to calculate tree layer biomass ( t·ha^-1^)

Shrub and herb biomass: the biomass obtained using the whole-harvest method is converted into shrub and herb biomass per unit area ( t·ha^-1^).

Litter biomass: convert the obtained ground litter biomass into litter biomass per unit area (t·ha^-1^).

Ecosystem biomass: calculated as the sum of the biomass of trees, shrubs, herbs, and litter (t·ha^-1^).

**(2) Productivity calculation**

Productivity describes the increase in biomass of an organism per unit time and unit area, and can be calculated by using the ratio of the increase in biomass within an interval to the time interval. This study used the biomass growth within 5 years after whole-tree harvesting to calculate productivity:

Tree and shrub layer productivity:

$$P_{wood}=\Delta B_{wood}/a$$

Where *P_wood_* is the productivity of the woody part of the tree or shrub (t·ha^-1^·year^-1^), *ΔB_wood_* is the biomass increment of the woody part of the tree or shrub (t·ha^-1^), *a* is 5 years; tree woody part including: stems, branches, barks, roots; shrub woody part including: branches and roots.

Leaf productivity is calculated separately for needle leaves and broad leaves, and then the sum is the total productivity:

Needle leaves:

$$P_{leaf}=\Delta B_{leaf}/a$$

Broad leaves:

$$P_{leaf}=Leaf biomass of the year$$

Where *P_leaf_* is the productivity of the tree or shrub leaves (t·ha^-1^·year^-1^), *ΔB_leaf_* is the biomass increment of the tree or shrub leaves (t·ha^-1^), *a* is 5 years.

Herb layer productivity: converted from the sum of annual aboveground and underground biomass (t·ha^-1^·year^-1^)

Ecosystem productivity: calculated as the sum of tree, shrub, and herb productivity (t·ha^-1^·year^-1^).

**(3) Nutrient stock calculation**

Plant layer (trees, shrubs, herbs): the C, N, P and K stocks of the plant layer are calculated by adding the product of the biomass of each organ in the plant layer and the C, N, P and K concentration of the corresponding organ (unit: t·ha^-1^).

Litter layer: the C, N, P and K stocks of the litter layer are the product of the ground litter biomass and the C, N, P and K concentration (t·ha^-1^).

Soil layer:

$$S_{n}=C_{n}\times BD_{n}\times L_{n}\times{10}^{-1}$$

$$S_{T}=\sum_{n=1}^{n} S_{n}$$

Where *S_n_* is the soil N, P or K stocks in the *n*-th layer (t ha-1), *C_n_*, *BD_n_* and *L_n_* represent the soil N, P or K concentration (g·kg^−1^), soil bulk density (g·cm^−3^) and soil depth (cm) in the *n*-th layer; 10^-1^ is the unit conversion factor; *S_T_* is the total soil layer N, P or K stocks (t·ha^-1^).

Ecosystem nutrient stock: ecosystem N, P and K stocks are the sum of N, P and K stocks in the plant layer, litter layer, and soil layer (t·ha^-1^).

**(4) Nutrient accumulation rate calculation**

Plant layer nutrient accumulation rate: the N, P and K accumulation rates in the tree, shrub, and herb layers are calculated by adding the product of the productivity of each organ in the plant layer and the N, P and K concentration of the corresponding organ (kg·ha^-1^·year^-1^).

Ecosystem nutrient accumulation rate: the N, P and K accumulation rate of the ecosystem is the sum of the N, P and K accumulation rates of trees, shrubs, and herbs (kg·ha-1·year-1).

**(5) Nutrient return calculation**

The ecosystem nutrient return amount is divided into two parts: the aboveground part and the underground part for calculation, and only the nutrient return of fine roots is considered for the underground part due to their rapid nutrient return characteristics (Norby and Jackson, 2000; Kong *et al.*, 2014; McCormack *et al.*, 2015).

Aboveground part: first calculate the product of the tree seasonal litter return biomass and the N, P and K concentration of the litter in that season, then calculate the product of the litter part on shrubs and herb (only leaf litter is considered here) and the N, P and K concentration, and finally perform the sum calculation (kg·ha^-1^·year^-1^).

Underground part: The N, P and K return amount of the underground part is the sum of the N, P and K return amounts of fine roots of each diameter class. The formula is as follows:

$$FR_{n}=C_{n}\times P_{n}\times T_{n}, n=1, 2, 3$$

$$FR_{T}=\sum_{1}^{n} FR_{n}$$

Where *FR_n_* is the N, P or K return amount of *n-th* diameter fine roots (kg·ha^-1^·year^-1^); *n*=1, 2, and 3 represent <0.5mm, 0.5-1mm and 1-2mm fine roots respectively; *C_n_*, *P_n_* and *T_n_* represent the N, P or K concentration of *n-th* diameter fine roots (g·kg^−1^), annual fine root productivity (t·ha^-1^) and annual fine root turnover rate (year^-1^) respectively; *FR_T_* is the total N, P and K return amounts of <2mm fine roots (kg·ha^-1^·year^-1^).

The dry weight of live fine roots and dead fine roots in the soil drill was added to calculate the live fine root biomass (g·m^-2^) and dead fine root biomass (g·m^-2^) of each sampling season in each plot. Fine root productivity (g·m^-2^·year^-1^) was calculated using the decision matrix method as below table. Fine root turnover rate (year^-1^) is defined as the ratio of annual fine root productivity (g·m^-2^·year^-1^) to the average fine root biomass in one year (g·m^-2^).

**TABLE │**Calculation of fine root productivity by decision matrix method

| If | Fine root production |
| --- | --- |
| ΔL+ΔD≧0 and ΔD≧0 | ΔL+ΔD |
| ΔL≧0 and ΔD≦0 | ΔL |
| ΔL≦0 and ΔD≦0 | 0 |

Note: L: live fine root mass; D: dead fine root mass; Δrepresents the changes in living fine root biomass or dead fine root mass.

Ecosystem plant nutrient return amount: the sum of the N, P and K return amounts of the plant aboveground part and underground part (kg·ha^-1^·year^-1^)

**(6) Calculation of nutrient characteristics at the ecosystem level for tree, shrub and herb functional groups**

Given that the analysis of the impact of nutrients at the plant organ level on the ecosystem nutrient cycling function is too complex and scattered, this study calculated the nutrient characteristics at the ecosystem level of the tree, shrub, and herb functional groups to characterize the nutrient situation of the plant nutrient pool. Plant organs are multifunctional and vary in nutrient content, and a biomass weighting method is used here to calculate functional group nutrient characteristics at the ecosystem level.

Tree layer:

$$E_{TPFG}=E_{TL}\times\frac{B_{TL}}{B_{TPFG}}+E_{TB}\times\frac{B_{TB}}{B_{TPFG}}+E_{TS}\times\frac{B_{TS}}{B_{TPFG}}+E_{TBA}\times\frac{B_{TBA}}{B_{TPFG}}+E_{TR}\times\frac{B_{TR}}{B_{TPFG}}$$

Where *E_TPFG_* is the C, N, P, K, C:N, C:P, N:P characteristics of tree layer at ecosystem level, *E_TL_*, *E_TB_*, *E_TS_*, *E_TBA_*, and *E_TR_* are C, N, P, K, C:N, C:P, N:P values of tree leaves, branches, stems, barks and roots respectively, the nutrient content unit is g·kg^-1^; *B_TL_*, *B_TB_*, *B_TS_*, *B_TBA_*, and *B_TR_* are biomass values of tree leaves, branches, stems, barks and roots, *B_TPFG_* is the total biomass value of the tree layer (t·ha^-1^).

Shrub layer:

$$E_{SPFG}=E_{SL}\times\frac{B_{SL}}{B_{SPFG}}+E_{SB}\times\frac{B_{SB}}{B_{SPFG}}+E_{SR}\times\frac{B_{SR}}{B_{SPFG}}$$

Where *E_SPFG_* is the C, N, P, K, C:N, C:P, N:P characteristics of shrub layer at ecosystem level, *E_SL_*, *E_SB_* and *E_SR_* are C, N, P, K, C:N, C:P, N:P values of shrub leaves, branches, and roots respectively, the nutrient content unit is g·kg^-1^; *B_SL_*, *B_SB_* and *B_SR_* are biomass values of shrub leaves, branches and roots, *B_SPFG_* is the total biomass value of the shrub layer (t·ha^-1^).

Herb layer:

$$E_{HPFG}=E_{HA}\times\frac{B_{HA}}{B_{HPFG}}+E_{HB}\times\frac{B_{HB}}{B_{HPFG}}$$

Where *E_HPFG_* is the C, N, P, K, C:N, C:P, N:P characteristics of herb layer at ecosystem level, *E_HA_* and *E_HB_* are C, N, P, K, C:N, C:P, N:P values of herb aboveground and belowground parts respectively, the nutrient content unit is g·kg^-1^; *B_HA_* and *B_HB_* are biomass values of herb aboveground and belowground parts, *B_HPFG_* is the total biomass value of the herb layer (t·ha^-1^).

**References:**

Kong, D., Ma, C., Zhang, Q., et al., 2014. Leading dimensions in absorptive root trait variation across 96 subtropical forest species. New Phytologist 203, 863-872, https://doi.org/10.1016/j.scitotenv.2022.160255.

McCormack, M.L., Dickie, I.A., Eissenstat, D.M., et al., 2015. Redefining fine roots improves understanding of below‐ground contributions to terrestrial biosphere processes. New Phytologist 207, 505-518, https://doi.org/10.1111/nph.13363.

Norby, R.J., Jackson, R.B., 2000. Root dynamics and global change: seeking an ecosystem perspective. New Phytologist 147, 3-12, https://doi.org/10.1046/j.1469-8137.2000.00676.x.
